# Clinical care patterns in the 10-years prior to primary total hip replacement: a matched population-based case-control analysis in England

**DOI:** 10.1186/s12916-026-05011-7

**Published:** 2026-06-24

**Authors:** Deepak Menon, Smitha Mathew, George Peat, Clara Hellberg, Aleksandra Turkiewicz, Andrea Dell’Isola, Martin Englund, Geraint Thomas, Dahai Yu

**Affiliations:** 1 Health Management Center, The First Affiliated Hospital of Zhengzhou University, Zhengzhou, 450052 China; 2 Primary Care Centre Versus Arthritis, School of Medicine, Keele University, Staffordshire, UK; 3 The Robert Jones and Agnes Hunt Orthopaedic Hospital NHS Foundation Trust, Oswestry, Shropshire, UK; 4 Centre for Applied Health & Social Care Research (CARe), School of Health and Social Care, Sheffield Hallam University, Sheffield, UK; 5 Clinical Epidemiology Unit, Department of Clinical Sciences Lund, Lund University, Orthopedics, Sweden

**Keywords:** Hip Osteoarthritis, Total Hip Replacement, Epidemiology, Case-Control Studies, Analgesics, Opioids, Primary Health Care, Electronic Health Records

## Abstract

**Background:**

The quality of evidence supporting recommendations for conservative hip osteoarthritis management limits the treatment options available to patients prior to surgery. The care individuals receive prior to their eventual total hip replacement (THR) is likely to be variable and complex. These long-term care patterns remain undefined.

**Methods:**

A population-based retrospective case-control study using linked primary and secondary care data (CPRD Aurum/HES APC) in England was conducted to identify the longitudinal prevalence of surgical, pharmacological and non-pharmacological care strategies in the 10 years preceding THR between 2007 and 2021. 480,598 individuals (240,299 THR recipients matched 1:1 to non-recipients) were identified. Prevalence rate ratios (PRRs) modelled chronological changes in care, referencing the 6-month period immediately prior to surgery (or index date for controls). Conditional logistic regression estimated odds ratios (ORs) for THR adjusted for confounders.

**Results:**

The prevalence of all pharmacological prescriptions increased over the 10-year observation period prior to THR, with an escalation in rates for opioids, oral NSAIDs and paracetamol starting at 24 months before surgery. All medication prescriptions were consistently higher in females across all time periods. The prevalence of sick leave and referrals for physiotherapy, hip imaging and pain clinic (non-pharmacological care) also increased. Hip injection prevalence increased from 0.03% at 12 months (6 to 12 month period) to 15.1% at 6 months (0 to 6 month period) pre-THR. Prescriptions for all analgesic sub-types, antidepressants, sick leave, and referrals for physiotherapy, pain clinic and hip imaging had increased odds of THR throughout the observation period. Strong opioid use in the final 6-month period represented the strongest association with surgery (OR 5.0 [95% CI 4.7, 5.2]). Younger patients consistently demonstrated a lower prevalence of medication prescriptions, and higher prevalence of sick leave issuance, relative to older groups.

**Conclusions:**

Escalating care is observed from 24 months prior to surgery among individuals who progress to THR. Prevalence of paracetamol and opioid prescriptions are high. Important age-related differences in care provision prior to THR relating to medication prescriptions and non-pharmacological interventions are present. This is accompanied by long-term elevated rates of sick leave in the working population.

**Supplementary Information:**

The online version contains supplementary material available at https://doi.org/10.1186/s12916-026-05011-7.

## Background

Hip osteoarthritis represents a growing source of disability worldwide. Recent estimates report a high age-standardised prevalence rate for individuals living globally with hip osteoarthritis (417.7 per 100,000 in 2020), with the associated age-standardised years lived with disability (YLDs) rate estimated to have increased by 6.0% from 1990 to 2020. Temporal trends project that 62.6 million individuals will be living with hip osteoarthritis globally by 2050, a 78.6% increase from 2020 [1].

Total hip replacement (THR) is a clinically and cost-effective treatment for end-stage hip osteoarthritis. Its effectiveness has been well established through evaluation of future revision risk and patient-reported outcome measures (PROMs) [2]. As the prevalence of symptomatic hip osteoarthritis increases, the demand for hip replacement surgery will grow. Data from the National Joint Registry (NJR) reports a 33.1% increase in primary THR procedures from 2014 (87,832 cases) to 2024 (116,901 cases) across England, Wales and Northern Ireland [3]. If resources and healthcare infrastructure fail to meet this expected increase in demand, time to surgery will inevitably increase. Prolonged waiting times are not only associated with significant deterioration in health-related quality of life (HRQoL), mental health and joint function [4], but also with a reduction in the long-term HRQoL and functional gains associated with the eventual THR [5, 6]. There is therefore a need to optimise the care of patients living with hip osteoarthritis prior to total joint arthroplasty.

The National Institute for Health and Care Excellence (NICE) has published recommendations for the management of hip osteoarthritis prior to referral for consideration of THR in the UK. Initial non-pharmacological treatment options include therapeutic exercise and weight reduction strategies, which may be self-directed or supervised. The use of pharmacological treatment to support non-pharmacological management is recommended with topical or oral non-steroidal anti-inflammatory drugs (NSAIDs). Individuals with unremitting symptoms affecting their activities of daily living, despite exhausting all pharmacological and non-pharmacological options, may then be considered for hip arthroplasty [7].

However, the quality of evidence supporting these recommendations is variable. For example, the evidence available comparing paracetamol to placebo, oral NSAIDs and glucosamine in osteoarthritis management was graded as generally being low quality to very low quality. Similarly, the evidence comparing strong opioids to placebo, NSAIDs and transdermal opioids was graded as generally being low to very low quality [7]. Based on this evidence, the routine prescription of paracetamol and strong or weak opioids are not recommended. Consequently, the treatment options available to patients for symptomatic management of hip osteoarthritis are limited.

Of note, there is also conflicting opinion regarding the use of intra-articular corticosteroid injections in hip osteoarthritis. Intra-articular hip injection of local anaesthetic and steroid may provide short-term improvement in pain and function [8], though their efficacy as a diagnostic adjunct for individuals with unclear symptoms remains uncertain [9]. Recent Osteoarthritis Research Society International (OARSI) guidelines do not recommend intra-articular corticosteroid injections for hip or polyarticular osteoarthritis [10]. However, NICE suggests that intra-articular corticosteroid injections may be used in patients with persistent symptoms when response to initial pharmacological treatment strategies is limited [7].

Given the variability in evidence-base behind established recommendations and the limited treatment options available for patients, the actual path taken by an individual living with symptomatic hip osteoarthritis prior to THR is likely to be variable, complex and influenced by multiple factors including patient preference, healthcare resource availability, clinician referral patterns and local policy.

Previous studies have been identified that focus on pre-arthroplasty healthcare utilisation, though these are limited to a specific healthcare procedure or a short observational period (typically 12 to 24 months) preceding THR [11–18]. Considering the median age at diagnosis for osteoarthritis is between 57 and 67 years in the UK [19, 20], whereas the median age for primary THR is 69 years [3], a longer observational time frame is crucial as it implies patients will have had several years of conservative management between diagnosis and surgery. It therefore remains unclear which care strategies are being employed, their chronological implementation and the association between specific care pathways and future joint replacement. Understanding these correlations could enable risk modelling based on the types of care received prior to surgery. Furthermore, whilst the main indication for THR is osteoarthritis [3], the underlying aetiology may vary by demographic. In younger patients, advanced hip dysplasia or femoroacetabular impingement (FAI) can be associated with secondary osteoarthritis [21, 22]. Care patterns preceding hip replacement are therefore likely to vary based on age profile, however this has not previously been explored.

This is a descriptive study which aims to define the national prevalence of different care strategies prior to primary THR for patients in England with comparison to a matched control population not undergoing surgery. By exploring how prevalence varies over time, the long-term care pathways preceding THR can be defined. The primary objective is to describe the longitudinal prevalence of surgical, pharmacological and other non-pharmacological care strategies received by patients within specific time-intervals in the 10 years prior to primary THR. Secondary objectives are (1) To place these care patterns in perspective by comparing the prevalence of care strategies within specific time-intervals with those of matched controls (non-recipients); (2) To quantify and compare the longitudinal prevalence of care strategies across different age groups to identify differences in care received prior to surgery between the young adult and elderly THR cohorts.

## Methods

### Study design and data source

A population-based retrospective case–control study was undertaken using primary care data from the Clinical Practice Research Datalink (CPRD) Aurum linked to Patient Level Index of Multiple Deprivation (IMD) and a secondary care data source (Hospital Episode Statistics Admitted Patient Care [HES APC]). CPRD Aurum contains routinely collected primary care records from General Practitioner (GP) practices using the EMIS software system across the United Kingdom [23]. At the time of data extraction, CPRD Aurum included records for 50.1 million patients of whom 16.6 million were actively registered across 1,605 general practices in England. HES APC provides data relating to all admissions to National Health Service (NHS) hospitals in England [24]. The English Index of Multiple Deprivation (IMD) is a composite measure of area-level socioeconomic deprivation based on the domains of income, employment, education and skills training, health deprivation and disability, barriers to housing and services, crime and the living environment [25]. In this study, patient postcode linked IMD was used, which is only available for patients in England.

The study was approved by the CPRD’s Research Data Governance (RDG) process (Protocol #24_003897) [26]. No additional ethical approval was required for the analysis of anonymised patient-level data. The study is reported in accordance with the Reporting of Studies Conducted using Observational Routinely Collected Data (RECORD) extension to the Strengthening the Reporting of Observational Studies in Epidemiology (STROBE) guidelines (Additional File 1) [27].

### Case definition

The study population was adults aged 30 years and over with a documented primary THR recorded within the HES APC database between 1st January 2007 and 31st March 2021. Primary THR was the outcome of interest, defined as replacement of a native hip joint. The index date was the date of the first recorded primary THR. In individuals with bilateral sequential primary THRs, only the first event would be included. Office of Population Censuses and Surveys (OPCS) Classification of Interventions and Procedures codes in HES APC were used to identify this case cohort, with linkage to CPRD Aurum data and Patient Level IMD subsequently performed. The identification of cases in a secondary care database provided a validated procedural index date and reduced the risk of misclassification. All cases required continuous registration in CPRD Aurum for at least 10 years prior to the index THR date for inclusion. The initial index year (2007) was selected to allow for a 10-year look-back period. Patients with a recorded diagnosis of autoimmune inflammatory arthritis and crystal arthropathy in the 10 years prior to the index THR were excluded from analysis.

### Control definition

For each case, controls were matched 1:1 using exact matching on sex, age group (stratified in 5-year intervals: 30–34, 35–39, 40–44, 45–49, 50–54, 55–59, 60–64, 65–69, 70–74, 75–79, 80–84, ≥ 85 years) and general practice. Controls were selected using incidence density sampling implemented via risk-set sampling from the general population using CPRD Aurum. Under this sampling framework, controls are identified from the pool of individuals at risk of the outcome at the time each case occurs [28]. Consequently, an individual’s probability of selection as a control is proportional to their contributed person-time at risk, and controls within the population at risk remain eligible to later become cases. When analysed as matched data, this approach generates an odds ratio that provides a reliable estimate of the incidence rate ratio [29]. Controls were each assigned an index date corresponding to the last day of the index year of their matched case to provide a consistent, year-anchored reference date for constructing the pre-specified 6-month windows used in the utilisation analyses and for applying uniform look-back requirements across the matched population. Given the very large denominator population in CPRD Aurum, assigning a control-specific index date at the exact event date for every matched set would require rebuilding and querying a distinctive risk set and time-window structure for each case date across a very large study base, substantially increasing computational burden and potentially reducing reproducibility of sampling across runs. The year-end assignment preserves calendar-year alignment between cases and controls while remaining computationally feasible [30]. This index date assignment occurs before risk-set sampling and does not use post-index information to select controls. Matching was performed using time-to-event and nested case-control risk-set sampling commands with the start date for the at-risk period defined as the date of registration for both cases and controls, and the time to index date measured in years. All eligible controls were additionally required to have at least 10 years of continuous registration in CPRD Aurum prior to the index year. Individuals with a documented THR in the 10-year period immediately preceding the index date were excluded. Additionally, those in the control group with a recorded diagnosis of autoimmune inflammatory arthritis or crystal arthropathy in the 10 years prior to the index date were excluded from analysis.

### Exposure and covariate definition

Exposure variables included surgical, pharmacological and non-pharmacological care strategies. OPCS codes were used to identify surgical care types from HES APC records, which included non-arthroplasty invasive procedures – hip arthroscopy, osteotomy of the proximal femur, hip or pelvis, and hip arthrodesis – as well as intra-articular hip injections of local anaesthetic with or without steroid. Pharmacological care strategies included prescriptions for topical and oral non-steroidal anti-inflammatory drugs (NSAIDs), opioids (categorised into weak and strong opioids), paracetamol, antidepressants, antiepileptics, bisphosphonates and corticosteroids. Corticosteroids were sub-categorised into ‘all’ and ‘systemic’ depending on their mode of administration. Systemic corticosteroids included intra-articular injections as well as oral, subcutaneous, intramuscular and intravenous corticosteroid use. These were identified within CPRD using Prodcodeids based on Dictionary of Medicines and Devices (DM + D) codes. Systematised Nomenclature of Medicine Clinical Terms (SNOMED CT) codes were used to identify all non-pharmacological care strategies from CPRD Aurum records. Non-pharmacological care strategies included general practice consultations (defined as any primary care consultation in which the individual recording the contact is a physician or clinician with GP-equivalent clinical responsibility who has the capacity to perform clinical assessment and make onward referral for secondary care), referral to physiotherapy or structured therapeutic exercise programmes (including self-referral), referral to radiology services for imaging of the hip (including X-ray [XR], computed tomography [CT], magnetic resonance imaging [MRI], ultrasound [US], or MR/CT arthrogram), exploration of absence of work due to sick leave, including through issuing of fit noes, and referrals to a pain clinic or pain management service.

In addition to deprivation, measured using linked Patient Level IMD, other demographic covariates assessed included age at index date, sex, geographical region (within England) and ethnicity (recorded based on CPRD Ethnicity Records). Lifestyle and clinical factors included were comorbidities, body mass index (BMI), history of hip injury, smoking status and drinking status, extracted from CPRD Aurum using SNOMED CT codes. For smoking status, drinking status and BMI, the most recent record prior to the index date was used. For all other lifestyle and clinical factors, covariates were defined based on the presence of at least one diagnostic code during the 10-year look-back period from index date. Hip injury was categorised as hip injury without fracture and hip fracture. Comorbidities were defined based on the Charlson Comorbidity Index (CCI) [31], which included the conditions of acquired immunodeficiency syndrome (AIDS), cancer (including localised solid tumour, leukaemia and lymphoma), metastatic solid tumour, congestive heart failure, chronic pulmonary disease, cerebrovascular accident (including transient ischaemic attack [TIA]), dementia, diabetes mellitus (categorised into uncomplicated diabetes and diabetes with end-organ damage), hemiplegia, myocardial infarction (MI), liver disease (categorised into mild liver disease and moderate-severe liver disease), peptic ulcer disease, peripheral vascular disease, moderate-severe renal disease and rheumatic disease.

The code lists used to define the outcome, exposures and co-variates have been presented in a supplementary file (Additional File 2).

### Statistical analysis

Contingency tables were used to describe the distribution of cases and controls by demographic factors (including age, sex, ethnicity, region) and other covariates (including BMI and comorbidity profile). The prevalence of having at least one recorded care strategy was estimated at 6-month intervals within the 10 years prior to the index date, overall and stratified by sex and other covariates, with 95% confidence intervals (CIs) derived using Poisson regression models. Prevalence rate ratios (PRRs) were calculated to compare the prevalence of each care strategy in each 6-month intervals against the most recent 6-month period, adjusting for age, sex, geographical region, ethnicity, neighbourhood deprivation, comorbidities, BMI, smoking status, drinking status and history of hip injury. Conditional logistic regression models were used to estimate odds ratios (ORs) for primary THR associated with each care strategy compared with controls within each 6-month period, adjusted for BMI, comorbidities, ethnicity, region, neighbourhood deprivation, smoking status, drinking status and history of hip injury. If smoking and drinking were not recorded, individuals were assigned to the ‘Never’ group. Missing data for BMI and IMD were handled using multiple imputation by chained equations under the assumption that data was missing at random [32]. The imputation model included all variables used in the regression analyses. Variable-specific imputation models were applied, with linear regression used for BMI and ordered logistic regression used for IMD. A total of 50 imputed datasets were generated and estimates were combined using Rubin’s rules to obtain overall effect estimates and standard errors.

## Results

312,860 individuals were identified who underwent primary THR between 1st January 2007 and 31st March 2021 in England and had ten or more years of continuous registration in the CPRD database prior to index date. Applying the study inclusion and exclusion criteria, 240,299 cases were identified. A flow diagram outlining the process by which cases and controls were selected is detailed in a supplementary file (Additional File 3).

Baseline demographic and clinical characteristics are presented in Table 1. Mean age of primary THR was 67 years and 23.8% were obese. Most individuals in the case group were female (62.2%) and of white ethnicity (96.1%). More data was derived from South East England (21.6%), with the highest proportion of patients being within the least deprived IMD decile (12.5%). Within the control group, a smaller proportion had BMI ≥ 25 Kg/m^2^ (68.2% vs. 74.7%) and prior hip injury with fracture (0.8% vs. 3.8%) or without fracture (0.2% vs. 0.7%) relative to the case population. A higher proportion were of ‘Other or Unknown’ ethnicity, and a lower proportion were of ‘White’ ethnicity within the controls relative to the case group (Other/Unknown: 7.9% vs. 0.9%; White: 88.5% vs. 96.1%). Other baseline clinical and demographic characteristics were similar between both groups.

**Table 1:** Patients’ baseline characteristics

Patients’ characteristics	Cases (*n* = 240,299)	Controls (*n* = 240,299)
**Sex, n (%)**		
Male	90,773 (37.78)	90,773 (37.78)
Female	149,526 (62.22)	149,526 (62.22)
**Age**,** Mean (SD)**	67.17 (12.71)	67.56 (13.59)
**Age Group**,** n (%)**		
30–44	13,061 (5.44)	13,061 (5.44)
45–54	27,670 (11.51)	27,670 (11.51)
55–64	51,323 (21.36)	51,323 (21.36)
65–74	75,544 (31.44)	75,544 (31.44)
75–84	55,650 (23.16)	55,650 (23.16)
85+	17,051 (7.10)	17,051 (7.10)
**BMI**,** Mean (SD)**	30.77 (10.55)	30.36(11.32)
**BMI Group**,** n (%)**		
Underweight (< 18.5)	2056 (0.86)	2900 (1.21)
Normal (18.5–24.9)	34,976 (14.56)	37,971 (15.80)
Overweight (25-29.9)	52,150 (21.70)	43,714 (18.19)
Obese ( > = 30)	57,287 (23.84)	43,883 (18.26)
Missing	93,830 (39.05)	111,831 (46.54)
**Comorbidities**,** n (%)**		
0	148,806 (61.93)	154,589 (64.33)
1	52,679 (21.92)	46,967 (19.55)
2	23,620 (9.83)	22,560 (9.39)
3+	15,194 (6.32)	16,183 (6.73)
**Ethnicity**,** n (%)**		
White	230,926 (96.10)	212,576 (88.46)
Mixed/Multiple	786 (0.33)	896 (0.37)
South Asian	2653 (1.10)	3410 (1.42)
Other Asian	1046 (0.44)	1452 (0.60)
Black	2631 (1.09)	2952 (1.23)
Other/Unknown	2257 (0.94)	19,013 (7.91)
**Region**		
North East	8516 (3.54)	8516 (3.54)
North West	45,179 (18.80)	45,179 (18.80)
Yorkshire and The Humber	8655 (3.60)	8655 (3.60)
East Midlands	5543 (2.31)	5543 (2.31)
West Midlands	42,856 (17.83)	42,856 (17.83)
East of England	10,545 (4.39)	10,545 (4.39)
London	29,280 (12.18)	29,280 (12.18)
South East	51,921 (21.61)	51,921 (21.61)
South West	37,804 (15.73)	37,804 (15.73)
**IMD**		
1 (least deprived)	29,932 (12.46)	29,726 (12.37)
2	27,234 (11.33)	26,877 (11.18)
3	27,981 (11.64)	27,313 (11.37)
4	27,888 (11.61)	27,171 (11.31)
5	23,695 (9.86)	23,509 (9.78)
6	24,021 (10.00)	24,076 (10.02)
7	23,091 (9.61)	23,326 (9.71)
8	19,670 (8.19)	20,228 (8.42)
9	18,774 (7.81)	19,398 (8.07)
10 (most deprived)	17,612 (7.33)	18,309 (7.62)
Missing	401 (0.17)	366 (0.15)
**Smoking**,** n (%)**		
Never/Not Recorded	159,561 (66.40)	166,875 (69.44)
Ex	48,847 (20.33)	41,147 (17.12)
Current	31,891 (13.27)	32,277 (13.43)
**Drinking**		
Never/Not Recorded	221,552 (92.20)	224,597 (93.47)
Ever	18,747 (7.80)	15,702 (6.53)
**Hip injury**		
Yes	1773 (0.74)	456 (0.19)
No	238,526 (99.26)	239,843 (99.81)
**Hip fracture**		
Yes	9038 (3.76)	1940 (0.81)
No	231,261 (96.24)	238,359 (99.19)

### Longitudinal prevalence of care strategies

**Fig. 1:**
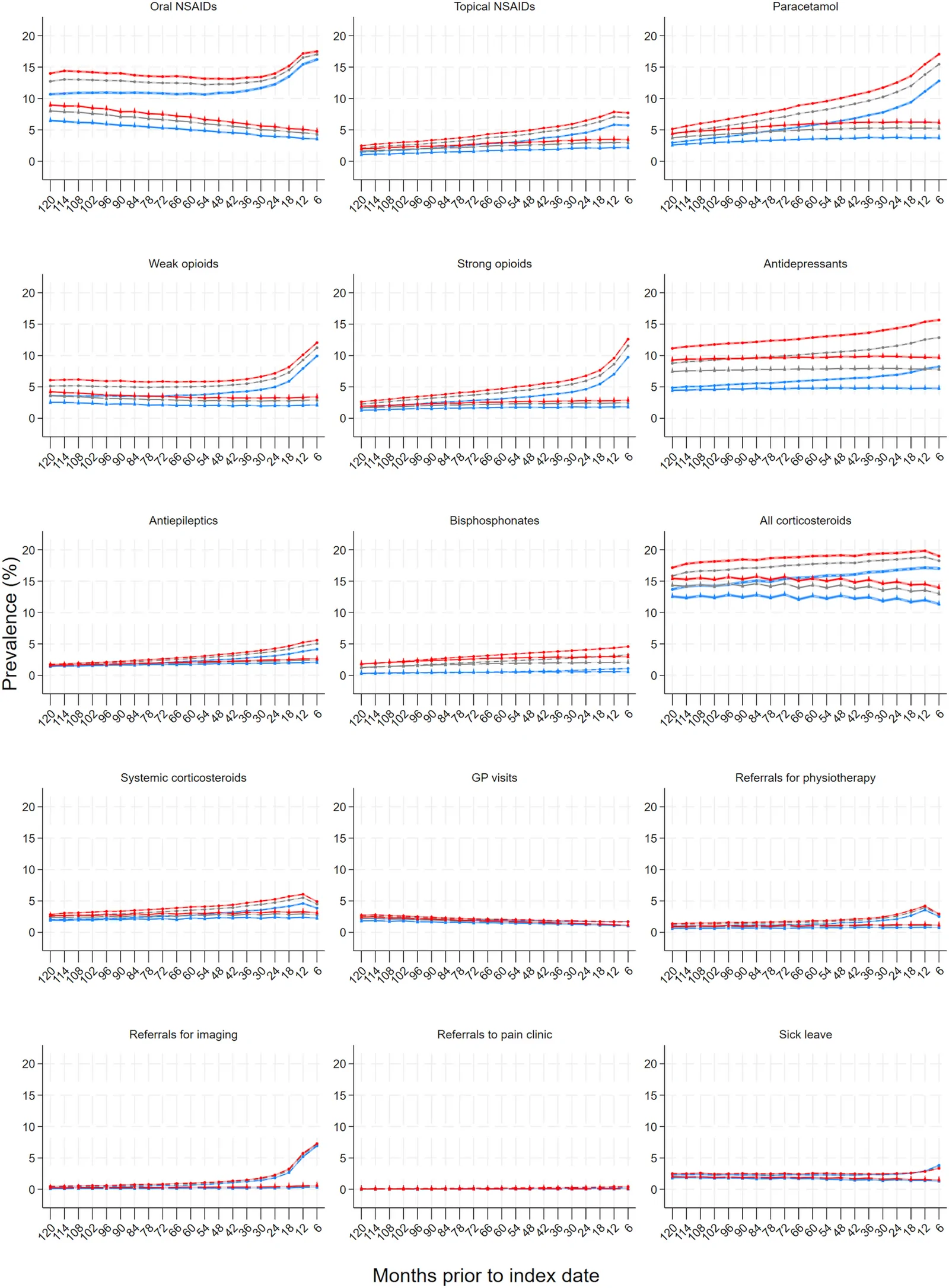
Prevalence of pharmacological and non-pharmacological care strategies within 10 years prior to index date. Prevalence and 95% CI of each care strategy within 10 years prior to index date in the case and control group, overall and stratified by gender. Grey, blue and red lines indicate the prevalence for overall, men and women, respectively. Triangle and dot lines represent the prevalence of the control and case group, respectively

The longitudinal prevalence of pharmacological (analgesic and non-analgesic medication) prescriptions and non-pharmacological interventions in the 10 years prior to index date for THR recipients compared to matched controls is presented in Fig. 1.

The prevalence of prescriptions for paracetamol, oral NSAIDs, topical NSAIDs, weak opioids and strong opioids was 15.5% (95% CI 15.3, 15.6), 17.0% (95% CI 16.9, 17.2), 7.0% (95% CI 6.9, 7.1), 11.3% (95% CI 11.1, 11.4) and 11.5% (95% CI 11.4, 11.7) respectively in the most recent 6-month period (0 to 6 months) prior to THR within the case cohort (Additional File 4, Supplementary Table 1). The prevalence of prescriptions for all analgesic medications was higher in the most recent 6-month period compared to the initial period 10 years (114 to 120 months) prior to index surgery, with prescriptions of paracetamol and strong opioids representing the largest percentage point increase. Between 120 and 24 months prior to THR, a gradual increase in prevalence was noted for prescriptions of topical NSAIDs, paracetamol and strong opioids, whereas the prevalence of prescriptions for oral NSAIDs and weak opioids remained static. The prevalence sharply increased for oral NSAIDs, weak opioids, strong opioids and paracetamol from 24 months preceding THR. This rate of prevalence increase to index surgery was greatest for prescriptions of weak and strong opioids. Female patients consistently demonstrated a higher prevalence of analgesic prescriptions through all time periods.

The prevalence of prescriptions for antidepressants, antiepileptics, bisphosphonates, all corticosteroids and systemic corticosteroids increased from 8.8% (95% CI 8.7, 8.9), 1.6% (95% CI 1.6, 1.7), 1.2% (95% CI 1.2, 1.3), 15.8% (95% CI 15.7, 16.0) and 2.5% (95% CI 2.5, 2.6) respectively in the initial period 10 years (114 to 120 months) prior to index THR to 12.9% (95% CI 12.7, 13.0), 5.1% (95% CI 5.0, 5.1), 3.3% (95% CI 3.2, 3.3), 18.3% (95% CI 18.1, 18.4) and 5.5% (95% CI 5.4, 5.6) respectively in the most recent 6-month period (0 to 6 months) prior to THR within the case cohort (Additional File 4, Supplementary Table 1). Antidepressants represented the largest percentage point increase. When stratified by sex, female patients consistently demonstrated a higher prevalence of non-analgesic prescriptions through all time periods.

Within the initial period, 10 years (114 to 120 months) prior to index surgery in case individuals, the prevalence of physiotherapy referral, hip imaging referral, referral to pain clinic and sick leave was 1.3% (95% 1.2, 1.3), 0.4% (95% CI 0.4, 0.4), 0.1% (95% CI 0.1, 0.1) and 2.4% (95% CI 2.4, 2.5) respectively, increasing to 2.8% (95% CI 2.7, 2.8), 7.2% (95% CI 7.1, 7.3), 0.4% (95% CI 0.3, 0.4) and 3.5% (95% CI 3.4, 3.6) in the most recent 6-month period (Additional File 4, Supplementary Table 1). Referral for hip imaging represented the largest percentage point increase, with a sharp rise noted from 24 months prior to index THR. A similar, though less pronounced upward trend, was observed for referral to physiotherapy and sick leave. Physiotherapy prevalence was noted to peak in the 12-month period (6 to 12 months) prior to surgery. Conversely, the prevalence of GP visits decreased from 2.5% (95% CI 2.5, 2.6) to 1.7% (95% CI 1.7, 1.8) over the 10-year observation period (Additional File 4, Supplementary Table 1).

The prevalence of intra-articular hip injections and non-arthroplasty surgical procedures are presented in Table 2. These were low across the observation period. Hip injection prevalence increased from 0.03% at 12 months (6 to 12 months pre-THR) to 15.1% at 6 months (0 to 6 months pre-THR) within the case group. Hip arthroscopy prevalence was 1.2% at 6 months prior to THR. Due to the absence of recorded events at multiple time-points in the case and control cohort, regression models to estimate ORs or PRRs did not converge.

**Table 2:** Prevalence of non-arthroplasty surgical care strategies (including hip injections) per 10,000 individuals

Months prior to index date	Hip injection		Hip arthrodesis	
	case; *n* = 240,299	control; *n* = 240,299	case; *n* = 240,299	control; *n* = 240,299
114–120	0.12	0.00	0.00	0.00
108–114	0.08	0.12	0.00	0.00
102–108	0.04	0.00	0.00	0.00
96–102	0.12	0.00	0.00	0.00
90–96	0.25	0.00	0.00	0.00
84–90	0.17	0.04	0.00	0.00
78–84	0.29	0.04	0.00	0.00
72–78	0.17	0.04	0.00	0.00
66–72	0.12	0.04	0.00	0.00
60–66	0.25	0.04	0.00	0.00
54–60	0.29	0.04	0.00	0.00
48–54	0.33	0.04	0.00	0.00
42–48	0.25	0.12	0.00	0.00
36–42	0.21	0.12	0.00	0.00
30–36	0.33	0.04	0.00	0.00
24–30	0.62	0.00	0.00	0.00
18–24	0.83	0.12	0.00	0.00
12–18	1.21	0.12	0.00	0.00
6–12	2.62	0.17	0.00	0.00
0–6	1513.32	29.05	0.29	0.04

	Hip arthroscopy		Hip osteotomy	
114–120	0.00	0.04	0.08	0.00
108–114	0.00	0.00	0.12	0.04
102–108	0.00	0.00	0.08	0.00
96–102	0.00	0.00	0.00	0.00
90–96	0.00	0.00	0.17	0.00
84–90	0.00	0.00	0.08	0.00
78–84	0.00	0.00	0.04	0.00
72–78	0.00	0.00	0.08	0.00
66–72	0.00	0.00	0.04	0.00
60–66	0.12	0.00	0.08	0.00
54–60	0.21	0.00	0.17	0.00
48–54	0.17	0.00	0.12	0.00
42–48	0.08	0.00	0.08	0.00
36–42	0.00	0.00	0.21	0.00
30–36	0.08	0.00	0.29	0.00
24–30	0.04	0.00	0.21	0.00
18–24	0.08	0.00	0.21	0.00
12–18	0.17	0.00	0.25	0.00
6–12	0.17	0.00	0.67	0.00
0–6	115.23	0.21	8.07	0.17

**Fig. 2:**
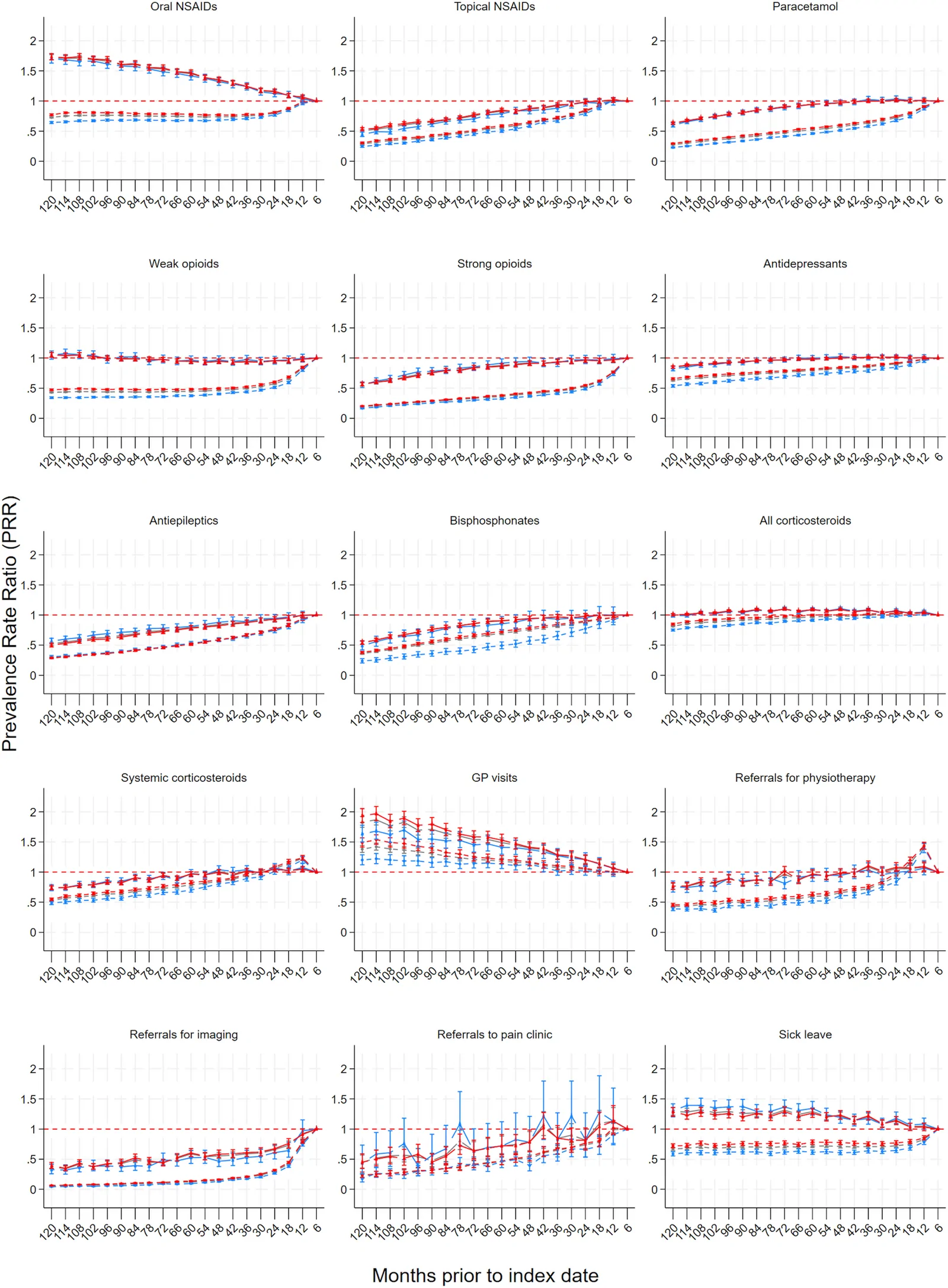
Adjusted PRR of pharmacological and non-pharmacological care strategies within 10 years prior to index date. PRR and 95% CI of each care strategy in each 6-month interval against the most recent 6-month period adjusted for sex, age, geographical region, ethnicity, neighbourhood deprivation, comorbidity status, BMI, smoking status, drinking status, and hip injury. Grey, blue and red lines indicate the PRR for overall, men and women, respectively. Triangle and dot lines represent the PRR of the control and case group, respectively

The temporal trends observed in the longitudinal prevalence of pharmacological prescriptions and non-pharmacological care events (Fig. 1) were mirrored by the adjusted PRRs (Fig. 2). Strong opioids, antiepileptics and hip imaging referral represented the largest relative increase in prevalence over the 10-year observation period prior to THR for analgesics, non-analgesic prescriptions and non-pharmacological care respectively. This was evidenced by low PRRs when comparing the prevalence at 10 years (114 to 120 months) to the immediate pre-operative (0–6 month) period (strong opioids 0.19 [0.19, 0.20], antiepileptics 0.29 [0.28, 0.31], hip imaging: 0.06 [0.05, 0.06]) (Additional File 4, Supplementary Table 2).

**Fig. 3:**
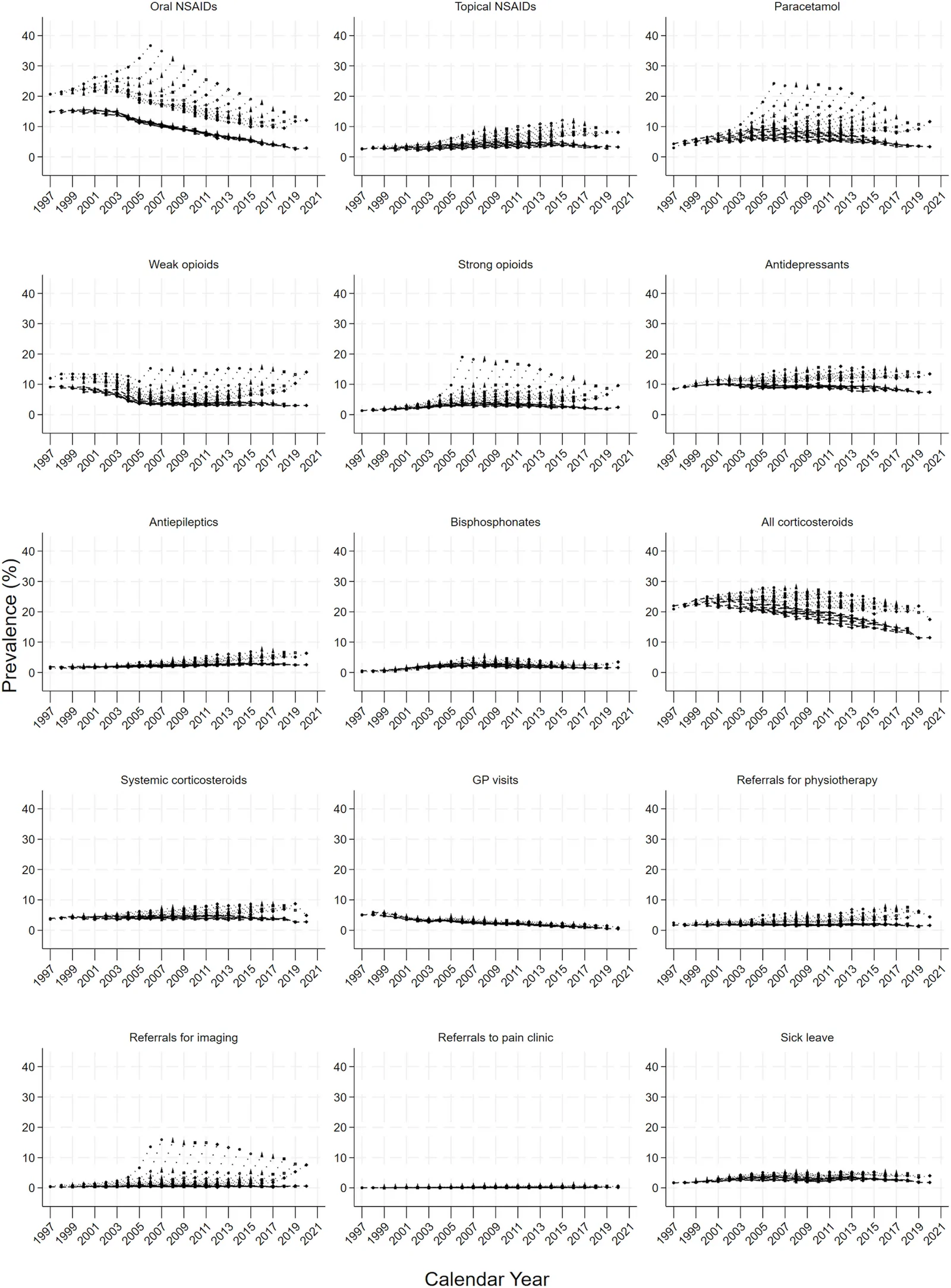
Period and cohort effect within 10 years of index date in case and control groups. Prevalence of each care strategy within 10 years prior to index date in case and control groups stratified by index year. Dashed and dotted lines represent the prevalence of the control and case group, respectively

A period effect was observed for prescriptions of oral NSAIDs, all corticosteroids and GP visits, characterised by a decline in overall prevalence observed over calendar time in both case and control groups (Fig. 3). A cohort effect was observed for oral NSAID prescriptions and hip imaging referrals within the case group. Specifically, individuals who underwent THR in later calendar years demonstrated a lower rate of prevalence increase over the observation period and a lower absolute prevalence in the more recent time periods prior to surgery. A similar, though less pronounced, trend was also observed for prescriptions of paracetamol and strong opioids within the case group. There was minimal evidence of a cohort effect across non-analgesic medication prescriptions.

**Fig. 4:**
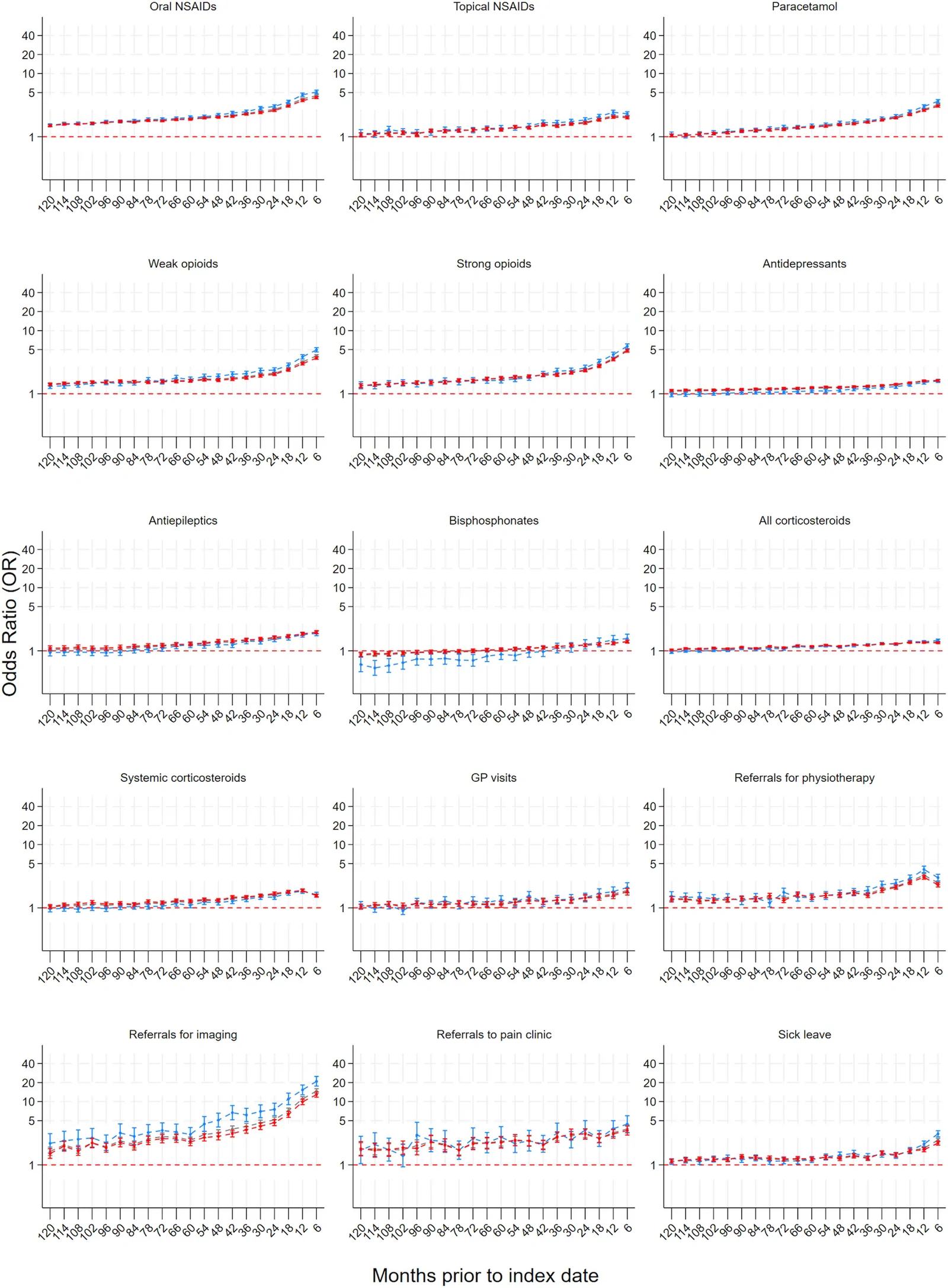
Adjusted ORs for THR associated with care strategies within each 6-month period before index date. ORs and 95% CI for primary THR associated with pharmacological and non-pharmacological care strategies within each 6-month period adjusted for geographical region, ethnicity, neighbourhood deprivation, comorbidity status, BMI, smoking status, drinking status, and hip injury. Grey, blue and red line indicate ORs for overall, men and women, respectively

Adjusted ORs characterise the association between each care type and primary THR (Fig. 4). Prescriptions of all analgesic sub-types and antidepressants, as well as physiotherapy referral, hip imaging referral, pain clinic referral and sick leave were associated with an increased odds of THR throughout the entire 10-year observation period (Additional File 4, Supplementary Table 3). This association was time-dependent with ORs rising as the index date approached. Among analgesics, the OR was greatest for oral NSAIDs at 10 years prior to index date (OR 1.5 [95% CI 1.5, 1.6]), while strong opioids represented the strongest association in the final 6-month period (OR 5.0 [95% CI 4.8, 5.2]). Antiepileptics represented the highest OR in the most immediate 6-month period among non-analgesic medications (OR 2.0 [95% CI 1.9, 2.0]). For non-pharmacological care, hip imaging referral represented the strongest association in the final 6-month period (OR 14.7 [95% CI 13.5, 16.0]) followed by pain clinic referral (OR 3.7 [95% CI 3.2, 4.3]) (Additional File 4, Supplementary Table 3).

Stratification by ethnicity, IMD and geographical region revealed consistent temporal trends across pharmacological and non-pharmacological care strategies (Additional File 5 – Supplementary Figs. 7 to 31, Supplementary Figs. 38 to 62, Supplementary Figs. 69 to 93).

A sensitivity analysis was performed by restricting the study population to the period 2007 to 2019, to evaluate the potential impact of the COVID-19 pandemic on pre-operative care pathways. 223,258 matched cases and controls were included in subgroup analysis. Estimates of prevalence in case and control individuals across all time periods prior to index date remained consistent with those observed in the primary analysis for pharmacological and non-pharmacological care strategies (Additional File 5, Supplementary Fig. 94). Similar findings were also observed for estimates and temporal trends of adjusted PRRs and ORs (Additional File 5, Supplementary Figs. 95 and 96). This suggests the primary results are robust and not affected by pandemic-related disruptions.

An additional sensitivity analysis was undertaken for antiepileptic and antidepressant medications, estimating PRRs and ORs after including epilepsy as well as depression, anxiety and stress as additional comorbidities for adjustment. No differences in point estimates for ORs, or PRRs across cases and controls, were identified in the overall cohort at all time periods prior to index date when compared to primary analysis (Additional File 4, Supplementary Tables 4 and 5; Additional File 5, Supplementary Figs. 97 and 98).

### Age-related differences in pharmacological and non-pharmacological care strategies

The prevalence of oral NSAID prescriptions in the initial period at 10 years (114 to 120 months) prior to THR was lowest in individuals aged 30 to 44 and greatest in those aged 75 to 84 years. Oral NSAID prevalence in the most recent 6-month period (0 to 6 months) was lowest in individuals aged 85 years and over, similar to those aged 30 to 44 years, and highest in the 55 to 64 years cohort. For all other analgesic medications, a greater prevalence was noted in older patient groups during both the initial and immediate 6-month periods prior to THR relative to younger patient cohorts. This difference was particularly pronounced for paracetamol prescriptions (Additional File 5, Supplementary Figs. 1 to 6). The ORs for future THR were higher in the younger patient subgroups for all analgesic prescriptions received during the initial period 10 years prior to index date. This age-based difference persisted in the immediate 6-month period for strong opioid prescriptions (Additional File 5, Supplementary Figs. 63 to 68).

The prevalence of all non-analgesic medication prescriptions was lowest in the youngest patient cohort (30 to 44 years) during both the initial 10-year (114 to 120 month) and immediate 6-month (0 to 6 month) period prior to THR. For prescriptions of antiepileptics, bisphosphonates and corticosteroids, prevalence increased with age group across both the initial 10-year and immediate 6-month time periods. For antidepressant prescriptions, prevalence peaked in the 55 to 64 years age group during both periods (Additional File 5, Supplementary Figs. 1 to 6). No age-related differences in ORs were observed at 10 years prior to index date for non-analgesic medication prescriptions. In the immediate 6-month period before index date, ORs for individuals prescribed antiepileptics were highest in the younger patient cohorts (30 to 54 years) (Additional File 5, Supplementary Figs. 63 to 68).

Similar trends in the prevalence of GP visits, physiotherapy referral, referral for hip imaging and pain clinic referral were noted over the observational period for case individuals leading up to index THR across all age groups. There was a higher prevalence of referral for hip imaging and physiotherapy in the older versus younger patient cohort in the immediate 6-month period with prevalence increasing across age groups (Additional File 5, Supplementary Figs. 1 to 6). Elevated odds for eventual THR were most pronounced in younger patient groups at specific time periods. In the immediate 6-month period prior to THR, the 30 to 44 years cohort demonstrated the highest ORs for THR with GP visits and physiotherapy referral compared to other age subgroups, and the 45 to 54 years cohort demonstrated the high OR with hip imaging referral (Additional File 5, Supplementary Figs. 63 to 68).

Sick leave prevalence was higher in the younger working-age cohort aged less than 65 years at the time of THR across all time periods preceding surgery, greatest across most time periods in individuals aged 55 to 64 years (Additional File 4, Supplementary Table 6). This age group was also associated with the largest relative increase in sick leave prevalence over the 10-year observation period prior to THR (PRR at 114 to 120 months 0.46 [0.44, 0.49]) (Additional File 4, Supplementary Table 7). Across all working-age cohorts (less than 65 years of age) temporal trends for sick leave were similar, with a rise in prevalence noted from approximately 18 months preceding THR (Additional File 5, Supplementary Figs. 1 to 3). OR was greatest in the 30 to 44 years group (1.87 [95% CI 1.28, 2.74]) and 45 to 54 years group (2.49 [95% CI 2.19, 2.84]) at 114 to 120 months and 0 to 6 months prior to index date respectively (Additional File 4, Supplementary Table 8).

## Discussion

This case-control study defines the national prevalence and longitudinal patterns of surgical, pharmacological and other non-pharmacological care strategies in the 10 years preceding primary THR in England from 2007 to 2021. The longitudinal associations between care strategies and future hip replacement surgery have also been investigated by comparing to matched individuals in the general population.

Among analgesic prescriptions, oral NSAIDs demonstrated the highest prevalence throughout the 10-year observation period for THR recipients. This aligns with current recommendations supporting their use in the symptomatic management of hip osteoarthritis [7] and existing evidence reporting oral NSAIDs are the most commonly used analgesic prior to THR [16, 33]. However, the observed period effect, with a decline in oral NSAID prescriptions over time noted in both case and control cohorts, suggests a shift in primary care prescribing habits over the last two decades in England. Similar trends are reported in the literature, reflecting a growing awareness and caution regarding the potential cardiovascular and gastrointestinal risk profiles associated with the long-term use of NSAIDs in routine clinical practice [34–36]. This study reports a relative shift away from oral NSAID use in the elderly cohort towards other analgesic options, which likely reflects the increased clinical caution due to its risk profile particularly in the older patients with higher comorbidity status and contraindications to oral NSAID use [37]. Inacio et al. similarly report a decreasing trend for NSAID prescriptions in an older Australian cohort in the one year prior to THR between 2001 and 2012 (adjusted PRR 0.95 [95% CI 0.94–0.95]) [18].

High prevalence of paracetamol, weak opioids and strong opioids were also noted throughout the observation period for individuals progressing to THR. Though trends in prescribing patterns varied between all analgesic types, there was a clear escalation in prescription rates starting from 24 months prior to THR of paracetamol and opioids peaking just prior to surgery. Previous population-based registry studies have also observed increasing rates of analgesic medication prescriptions in the short-term (one to two years) before hip replacement surgery [14, 16, 38]. These findings highlight a potential discordance with current national guidelines, which advise against the routine offer of paracetamol or weak opioids, unless used infrequently for short-term pain relief and other analgesic treatments are contraindicated or ineffective, or strong opioids [7]. Furthermore, the escalating use of antiepileptic prescriptions observed over the 10-year observation period is likely due to the inclusion of gabapentinoids within this medication category. This can be used to treat neuropathic pain, which is thought to represent a component of osteoarthritis-related pain [39]. The finding that strong opioids and antiepileptics emerged as the analgesic and non-analgesic medication subtypes with the strongest association with THR in the immediate 6-month period further highlights a discordance between national prescribing practices and current recommendations.

The observed escalation in the prevalence of oral NSAIDs, paracetamol and opioids from two years prior to THR, increasing up to the time of surgery, may reflect a critical threshold of symptomatic deterioration at this stage in the disease process. A sharp rise in prevalence of opioid prescribing from 2 years prior to total knee replacement has previously been observed [30]. These findings may also represent a shift towards more aggressive short-term pain management therapies, such as strong opioid prescriptions, once patients have been placed on a waiting list to ensure adequate symptom control prior to a planned surgical date. A similar trend is also noted across the non-pharmacological care strategies of referral for hip imaging, referral for physiotherapy and sick leave. The concurrent increase in these care strategies may reflect evolving GP prescribing and referral trends designed to document symptomatic failure and the exhaustion of conservative measures to justify onward referral for consideration of joint replacement surgery at a time when access to surgery is being limited due to increasing demand and financial pressure [40]. Furthermore, a small number of patients undergoing THR will experience complications – evidence that conservative treatment has failed before proceeding with joint replacement is an important medicolegal consideration [41].

Sustained associations have been identified throughout the 10-year observation period prior to THR for paracetamol, NSAID and opioid prescriptions. Similarly, referrals to physiotherapy, hip imaging and pain clinic as well as issuance of sick leave had a consistent positive association (OR > 1.0) with THR. These findings highlight significant differences in analgesic and non-pharmacological care utilisation between THR cases and controls identified from the general population. The strength of the association between analgesic prescriptions and THR was time-dependent and non-linear, increasing as index surgery date approached. Similar temporal trends in ORs were also observed for non-pharmacological interventions. These healthcare utilisation gaps therefore appear to widen towards surgery. Referral to pain clinic and for hip imaging represented the strongest association with THR in the short-term pre-operative period, suggesting radiological assessment and specialised pain management are the final definitive steps undertaken by clinicians to confirm diagnosis and relieve symptoms prior to arthroplasty. Existing prognostic models predicting joint replacement in individuals with hip osteoarthritis have included analgesic medications as candidate predictors [42, 43]. However, direct inferences relating to the utility of these medications as independent prognostic factors for THR are unable to be made from this study, as the majority of individuals in the control group will not have a diagnosis of hip osteoarthritis which would act as a confounder.

Literature quantifying physiotherapy utilisation prior to arthroplasty is limited. In a cross-sectional study, Postler et al. reported that 43% of patients with hip and knee osteoarthritis in Germany received a physical therapy prescription [44]. To our knowledge, no previous studies have reported the longitudinal prevalence of physiotherapy referrals rates for hip osteoarthritis. In this study, the recorded prevalence of physiotherapy referrals remained low throughout the observation window, reaching a peak of 4.0% in the 6 to 12 month period preceding THR. However, unlike pharmacological management where continuous repeat prescriptions are recorded in primary care records, a physiotherapy referral is typically a single administrative event that initiates a prolonged period of care. Consequently, measuring referral prevalence within discrete time-intervals captures only the initiation of therapy at that time point rather the ongoing duration of conservative treatment. Given that the timing of initial referral will vary based on symptoms and patient factors, these referral events will be spread over the 10-year observation period. Prevalence within a single time window is therefore expected to be low and underestimates the overall burden of physiotherapy engagement prior to THR. Furthermore, these findings likely represent an underestimation of total utilisation, as the CPRD Aurum database does not capture care accessed through private healthcare pathways. It also does not account for self-management strategies or verbal advice, including exercise sheets, provided by the healthcare provider during routine primary care consultations.

Although the prevalence of GP visits was higher in the THR group across all time periods, a period effect was observed with a decline in prevalence across both case and control cohorts over the 10-year observation period. This trend aligns with recent observational data from the CPRD Aurum database, which has highlighted a period of decreasing GP consultation counts between 2012 and 2019 [45]. However, this decline may also reflect a shift in service delivery toward telephone or remote consultations, which may not have been captured by the codes utilised in this analysis [46]. Importantly, this data represents all-cause consultations and were not specific for hip pain or osteoarthritis.

A high proportion of individuals with end-stage hip osteoarthritis have depression or related mood disorders [47]. In this study, the prevalence of antidepressants at all stages in both the case and control cohort was assessed and found to be high, and the prevalence was higher in the case group with a gradual increase over the observation period observed. Limited data reporting the temporal trends of antidepressant prescription use prior to THR exists. A prospective observational study from Norway reports static antidepressant user rates prior to hip replacement, though only a one-year preoperative period was evaluated [14]. The sustained association (OR > 1) between antidepressant use and future THR over the 10-year observation period may support previous longitudinal data reporting increased risk of depression in individuals with hip or knee osteoarthritis [48]. However, this association may also be attributed to secondary indications for antidepressants in chronic pain management. The interplay between hip osteoarthritis and depression is complex, with a bidirectional relationship proposed [49].

The prevalence of intra-articular hip injections remained low throughout most of the observation period, with a marked escalation noted only in the immediate 6-month period prior to THR for case individuals. The prevalence observed is lower than reported in previous literature [50, 51] and may reflect international differences in treatment pathways. Furthermore, to strictly define intra-articular injection rather than soft tissue injection such as for trochanteric bursitis, OPCS procedure codes were utilised to identify this procedure within secondary care. Therefore, a proportion of individuals who were referred for US-guided hip injections within a community care setting would be missed leading to a potential underestimation in prevalence. Given that the median waiting time to hip replacement in England was approximately 174 days in 2024 [52], the utilisation of hip injections represents a late-stage clinical intervention, likely either contributing to the decision for surgical listing or forming part of the management pathway once a decision has been made. This trend persists despite intra-articular injections being associated with increased risk of periprosthetic joint infections if used within three months prior to THR [53, 54]. A slight rise in prevalence was also observed within the control group in the 0 to 6 month period preceding index date. However, this may be attributed to the risk-set sampling method, where potential future cases may be included in the control group. Consequently, utilisation of care strategies, including injections, may be captured for these individuals prior to their own index case date.

The prevalence of conservative care strategies and differences in their temporal trends prior to THR within young adult patients aged 30 to 64 years have not previously been characterised. Significant age-related differences were observed in the prevalence and longitudinal trends of both pharmacological and non-pharmacological care strategies preceding THR. Across all time periods, younger patient cohorts consistently demonstrated a lower prevalence of both analgesic and non-analgesic medications prescriptions relative to the older patient population, with prevalence rising across increasing age groups. Polypharmacy has been shown to increase with age [55], which may relate to an increase in comorbidity status [56], partially accounting for this finding. However, this disparity was also evident in non-pharmacological interventions, with a lower prevalence of referral for hip imaging and physiotherapy noted in younger patient groups as index THR date approached. The lower recorded prevalence of care strategies in younger THR cohorts is likely driven by clinical risk management and health policy artifacts rather than a deficit in care. Evidence from Bayliss et al. indicates that the lifetime risk of revision (LTRR) increases with decreasing age – estimated at 30% for males aged 50 to 54 verses < 5% for those aged 75 years and over [57]. This high risk mandates a strategy of surgical delay for younger patients, meaning diagnostic imaging and physiotherapy referrals often occur years prior to the immediate pre-operative window. Additionally, pharmacological disparities are likely influenced by the English prescription policy, which provides free medication only to those aged 60 years and over [58]. Younger patients are consequently more inclined to utilise over-the-counter analgesics including NSAIDs. Because these non-prescription acquisitions are excluded from the CPRD database, the result is an artificial reduction in the documented pharmacological care of younger cohorts.

Conversely, and as expected, sick leave prevalence was higher in those of working age. Particularly high rates of sick leave were noted in the immediate 18-month period prior to surgery across all working-age sub-groups. Individuals working with musculoskeletal disorders including arthritis, have a higher risk of sick leave relative to healthy employees [59, 60]. Stigmar et al. similarly report increased sick leave rates within a hip osteoarthritis cohort from Sweden relative to matched controls within 12 months of THR [15]. However, our results suggest increased sick leave rates compared to controls at all time periods throughout the 10-year observative period. THR being undertaken in individuals of working age is therefore accompanied by long-term elevated rates of sick leave. Given length of sickness absence is associated with ability to return to work following THR [61], delaying hip replacement in the younger patient with confirmed hip osteoarthritis and failure of conservative management may be associated with increased economic cost to both the individual and society [59–61].

Furthermore, where pharmacological or non-pharmacological treatment was given, the association with future hip replacement was generally stronger in the younger patient cohorts across all time points. This suggest that recorded care events in younger individuals are more specific for future THR relative to an older patient, where potential confounding pathology or competing health concerns may mean that prescribed care is not solely attributable to the hip-related morbidity [62].

A key strength of this study is the utilisation of a large, nationally representative dataset which allowed for a matched case-control analysis to be performed, quantifying associations between care strategies and joint replacement. Furthermore, the extended 10-year observation period with narrow 6-month time windows enabled the mapping of long-term temporal trends with high granularity and has identified novel care patterns preceding THR. However, there are several limitations to this study which must be acknowledged. Firstly, these findings reflect the prevalence of prescribing and referral rates documented within the primary care record. This does not necessarily equate to utilisation or adherence to care which may limit the conclusions drawn from care trajectory analysis. Secondly, paracetamol and NSAIDs are frequently purchased over-the-counter, without requirement for a prescription. The recorded prevalence of these medications therefore likely represents an underestimation of their actual use within the study population. Furthermore, CPRD does not link medication prescriptions to specific diagnostic codes. Definitively attributing prescriptions to symptoms from hip osteoarthritis is therefore not possible. For example, analgesics may be prescribed for other sources of pain. Similarly, certain medication classes, such as antidepressants and antiepileptics, can have overlapping indications. However, the contribution of non-osteoarthritis causes for these prescriptions is expected to be reduced by our use of matching and adjustment for comorbidities, including depression and epilepsy as part of a sensitivity analysis, although residual confounding is likely to remain. Thirdly, a limitation of using HES data is that it primarily captures publicly funded THR procedures [63]. There is therefore a risk of individuals undergoing privately funded THRs in the independent sector being misclassified within the control group. Also, the pre-operative care patterns between privately versus publicly funded THR may differ, which may limit the generalisability of these results. Fourthly, whilst the data indicates a discordance between national prescribing practices and guidelines, the study cannot account for local policy variations that may guide GP referral or pain management thresholds. This may potentially lead to geographical variations in practice that influences the observed trends. Fifthly, although the extent of missing data for IMD was balanced between case and control groups, missingness for BMI was higher in the control cohort. This likely relates to the required documentation of height and weight for surgical referral and pre-operative assessment. Although multiple imputation was used to reduce risk of bias, some uncertainty will remain in the point estimates for these covariates. Moreover, although potential confounding variables were adjusted for during analysis, residual confounding effects may remain. One important potential confounder is hip osteoarthritis due to a lack of complete registrations in primary care data. Sixthly, the requirement for 10 years of continuous primary care registration may have introduced selection bias by excluding younger individuals who may more frequently move practices, or those with significant comorbidities leading to nursing home transfer and deregistration. However, this look-back period length does allow for a sufficiently long and complete window to capture accurate longitudinal care trajectories. Notably, since the primary outcome is THR, and survival to the index date is a requirement for inclusion, healthy survival bias is unlikely to have affected the study results. Finally, the study population is not restricted to individuals with only hip osteoarthritis. Although individuals with autoimmune and crystal arthropathies were excluded, THR can also be indicated for acute trauma or fracture which may lead to outcome misclassification. Our results may therefore underestimate the true prevalence of care strategies prior to hip joint replacement for osteoarthritis in the case group, as those who undergo THR for trauma will likely lack the sustained healthcare utilisation patterns characteristic of individuals with symptomatic, progressive hip osteoarthritis. However, national registry-level data suggests indications other than osteoarthritis account for a small proportion of individuals undergoing primary THR, with 91.2% of primary THRs being performed for osteoarthritis between April 2003 and December 2024 [3]. In this analysis, 3.8% of the case group had a recorded history of hip fracture (compared to 0.8% in the matched controls). Given that this accounts for a small proportion of the total study population, it is unlikely to have skewed the longitudinal care patterns observed. Furthermore, previous evidence has identified significant underrecording of osteoarthritis in primary care records and suggest using joint replacement as a measure of disease burden [64].

## Conclusions

In conclusion, this population-based case-control analysis provides a comprehensive summary of the longitudinal care pathways in the 10 years preceding primary THR in England. High prevalence of oral NSAIDs, paracetamol and opioids were observed at all time periods, with a marked escalation in both pharmacological and non-pharmacological care in the two years preceding hip joint replacement. Furthermore, significant age-related differences were identified in the prevalence of medication prescriptions, hip imaging referral and physiotherapy highlighting the distinct pre-operative care patterns in the young adult patient group undergoing THR. This is accompanied by long-term elevated rates of sick leave in the working population prior to hip replacement surgery. This study was designed as a descriptive study with no hypothesis that care causes THR. Causal inferences should therefore not be made, with the lack of information about hip osteoarthritis diagnosis among cases and controls further precluding any causative assumptions regarding the relationship between care strategies and joint replacement.

## Supplementary Information

Below is the link to the electronic supplementary material.

Supplementary Material 1: Additional File 1: The RECORD statement: Checklist of items, extended from the STROBE statement, that should be reported in observational studies using routinely collection health data.

Supplementary Material 2: Additional File 2: Code List: Spreadsheet documenting the code lists used to define the outcome, exposures and other co-variates.

Supplementary Material 3: Additional File 3: Workflow of Selection of Matched Pairs: Flow diagram summarising the process by which cases and matched controls were generated.

Supplementary Material 4: Additional File 4: Supplementary Tables: File containing Supplementary Tables 1 to 6

Supplementary Material 5: Additional File 5: Supplementary Figures 1 to 6: Prevalence Stratified by Age. Supplementary Figures 7 to 12: Prevalence Stratified by Ethnicity. Supplementary Figures 13 to 21: Prevalence Stratified by Region. Supplementary Figures 22 to 31: Prevalence Stratified by IMD. Supplementary Figures 32 to 37: PRR Stratified by Age. Supplementary Figures 38 to 43: PRR Stratified by Ethnicity. Supplementary Figures 44 to 52: PRR Stratified by Region. Supplementary Figures 53 to 62: PRR Stratified by IMD. Supplementary Figures 63 to 68: OR Stratified by Age. Supplementary Figures 69 to 74: OR Stratified by Ethnicity. Supplementary Figures 75 to 83: OR Stratified by Region. Supplementary Figures 84 to 93: OR Stratified by IMD. Supplementary Figures 94 to 98: Sensitivity Analysis.

## Data Availability

The data that supports the findings of this study was obtained from the Clinical Practice Research Datalink (CPRD). CPRD data governance does not allow us to distribute patient data to other parties. Researchers may apply for data access at [http://www.CPRD.com/](http:/www.CPRD.com) . The CPRD RDG protocol has been made available to reviewers.

## Abbreviations

THR: Total hip replacement
YLD: Years lived with disability
PROM: Patient–reported outcome measure
NJR: National Joint Registry
HRQoL: Health–related quality of life
NICE: National Institute for Health and Care Excellence
NSAID: Non–steroidal anti–inflammatory drugs
OARSI: Osteoarthritis Research Society International
FAI: Femoroacetabular impingement
CPRD: Clinical Practice Research Datalink
IMD: Index of Multiple Deprivation
HES APC: Hospital Episode Statistics Admitted Patient Care
GP: General practitioner
NHS: National Health Service
RDG: Research Data Governance
RECORD: Reporting of Studies Conducted using Observational Routinely Collected Data
STROBE: Strengthening the Reporting of Observational Studies in Epidemiology
OPCS: Office of Population Censuses and Surveys
DM + D: Dictionary of Medicines and Devices
SNOMED CT: Systematised Nomenclature of Medicine Clinical Terms
XR: X–ray
CT: Computed tomography
MRI: Magnetic resonance imaging
US: Ultrasound
BMI: Body mass index
CCI: Charlson Comorbidity Index
AIDS: Acquired immunodeficiency syndrome
TIA: Transient ischaemic attack
MI: Myocardial infarction
CI: Confidence intervals
PRR: Prevalence rate ratio
OR: Odds ratio
LTRR: Lifetime risk of revision
