# Supplementary material for: Clinical care patterns in the 10-years prior to primary total hip replacement: a matched population-based case-control analysis in England
Source: BMC Med. 2026 Jun 24;24:480. doi: 10.1186/s12916-026-05011-7 (PMC13560152; doi:10.1186/s12916-026-05011-7)
Supplement: Supplementary file 2 [file 12916_2026_5011_MOESM3_ESM.docx]

**Workflow of Selection of Matched Pairs**

Patients who underwent THR between 1/1/2007 and 31/3/2021 and ≥10 years of continuous registration in the CPRD prior to index date

N=312,860

Patients who had no recorded THR and ≥10 years of continuous registration in the CPRD prior to index date and 1:1 matched with age, gender and practice ID

N=312,860

Total patients

N=625,720

Patients with linkage

N=596,479

Patients without diagnosis of autoimmune inflammatory arthritis in the 10 years prior to index date

N=559,293

Patients with age ≥ 30 years

N=545,935

Patients with match ID

N=480,598

Patients with recorded diagnosis of autoimmune inflammatory arthritis in the 10 years prior to index date

N=37,186

Excluded due to age <30 years N=13,358

All patients with THR OPCS codes in HES records from 1997 to 2021

N= 1,210,237

Patients eligible for including in the control

N= 50,055,001

Patients without linkage

N=29,241
