# Supplementary material for: Clinical care patterns in the 10-years prior to primary total hip replacement: a matched population-based case-control analysis in England
Source: BMC Med. 2026 Jun 24;24:480. doi: 10.1186/s12916-026-05011-7 (PMC13560152; doi:10.1186/s12916-026-05011-7)
Supplement: Supplementary file 3 [file 12916_2026_5011_MOESM4_ESM.docx]

**Supplementary Table 1**

Prevalence and 95% CIs of pharmacological and non-pharmacological care strategies within 10 years prior to index date in case and control groups, overall and by gender.

| Months prior to index date | Oral NSAIDs | | | | | |
| --- | --- | --- | --- | --- | --- | --- |
|  | Overall | | Male | | Female | |
|  | Cases; n=240,299 | Controls; n=240,299 | Cases; n=90,773 | Controls; n=90,773 | Cases; n=149,526 | Controls; n=149,526 |
| 114-120 | 12.75 (12.61, 12.88) | 8.02 (7.91, 8.13) | 10.68 (10.48, 10.88) | 6.49 (6.33, 6.65) | 14.00 (13.83, 14.18) | 8.95 (8.81, 9.10) |
| 108-114 | 13.04 (12.91, 13.18) | 7.89 (7.78, 8.00) | 10.79 (10.59, 10.99) | 6.36 (6.20, 6.52) | 14.41 (14.23, 14.59) | 8.82 (8.67, 8.96) |
| 102-108 | 13.02 (12.88, 13.15) | 7.81 (7.71, 7.92) | 10.90 (10.70, 11.10) | 6.18 (6.03, 6.34) | 14.30 (14.13, 14.48) | 8.80 (8.66, 8.95) |
| 96-102 | 12.95 (12.82, 13.09) | 7.60 (7.49, 7.70) | 10.91 (10.71, 11.12) | 6.14 (5.98, 6.29) | 14.19 (14.01, 14.37) | 8.48 (8.34, 8.63) |
| 90-96 | 12.87 (12.73, 13.00) | 7.44 (7.34, 7.55) | 10.95 (10.74, 11.15) | 5.93 (5.78, 6.09) | 14.03 (13.86, 14.21) | 8.36 (8.22, 8.50) |
| 84-90 | 12.84 (12.71, 12.97) | 7.10 (6.99, 7.20) | 10.89 (10.69, 11.09) | 5.75 (5.60, 5.90) | 14.02 (13.85, 14.20) | 7.91 (7.77, 8.05) |
| 78-84 | 12.67 (12.53, 12.80) | 7.07 (6.97, 7.17) | 10.94 (10.74, 11.14) | 5.65 (5.50, 5.80) | 13.71 (13.54, 13.89) | 7.93 (7.80, 8.07) |
| 72-78 | 12.55 (12.42, 12.68) | 6.78 (6.68, 6.88) | 10.88 (10.68, 11.09) | 5.47 (5.33, 5.62) | 13.56 (13.39, 13.74) | 7.58 (7.44, 7.71) |
| 66-72 | 12.49 (12.36, 12.62) | 6.66 (6.56, 6.76) | 10.82 (10.62, 11.02) | 5.30 (5.15, 5.45) | 13.50 (13.33, 13.67) | 7.49 (7.35, 7.62) |
| 60-66 | 12.48 (12.34, 12.61) | 6.46 (6.36, 6.55) | 10.69 (10.49, 10.89) | 5.21 (5.06, 5.35) | 13.56 (13.39, 13.73) | 7.21 (7.08, 7.35) |
| 54-60 | 12.41 (12.28, 12.54) | 6.27 (6.18, 6.37) | 10.80 (10.60, 11.00) | 4.99 (4.84, 5.13) | 13.38 (13.21, 13.56) | 7.05 (6.92, 7.18) |
| 48-54 | 12.21 (12.08, 12.34) | 5.99 (5.89, 6.08) | 10.64 (10.44, 10.84) | 4.88 (4.74, 5.02) | 13.16 (12.99, 13.33) | 6.65 (6.53, 6.78) |
| 42-48 | 12.30 (12.17, 12.43) | 5.80 (5.71, 5.89) | 10.89 (10.68, 11.09) | 4.67 (4.53, 4.80) | 13.16 (12.99, 13.33) | 6.49 (6.36, 6.61) |
| 36-42 | 12.31 (12.18, 12.44) | 5.59 (5.49, 5.68) | 10.95 (10.75, 11.16) | 4.55 (4.41, 4.68) | 13.13 (12.96, 13.30) | 6.21 (6.09, 6.34) |
| 30-36 | 12.55 (12.41, 12.68) | 5.38 (5.29, 5.47) | 11.25 (11.04, 11.45) | 4.41 (4.28, 4.55) | 13.33 (13.16, 13.51) | 5.96 (5.84, 6.08) |
| 24-30 | 12.76 (12.63, 12.90) | 5.04 (4.95, 5.13) | 11.66 (11.46, 11.87) | 4.06 (3.93, 4.19) | 13.43 (13.26, 13.60) | 5.63 (5.52, 5.75) |
| 18-24 | 13.34 (13.20, 13.47) | 4.94 (4.85, 5.02) | 12.26 (12.05, 12.47) | 3.95 (3.83, 4.08) | 13.99 (13.81, 14.16) | 5.53 (5.42, 5.65) |
| 12-18 | 14.55 (14.41, 14.69) | 4.70 (4.62, 4.79) | 13.50 (13.27, 13.72) | 3.88 (3.75, 4.00) | 15.19 (15.01, 15.37) | 5.21 (5.09, 5.32) |
| 6-12 | 16.52 (16.37, 16.67) | 4.55 (4.47, 4.63) | 15.45 (15.21, 15.68) | 3.63 (3.51, 3.76) | 17.17 (16.98, 17.36) | 5.10 (4.99, 5.22) |
| 0-6 | 17.02 (16.87, 17.17) | 4.34 (4.26, 4.42) | 16.22 (15.98, 16.46) | 3.56 (3.43, 3.68) | 17.51 (17.31, 17.70) | 4.81 (4.70, 4.92) |
| Topical NSAIDs | | | | | | |
| 114-120 | 2.10 (2.04, 2.16) | 1.66 (1.61, 1.71) | 1.49 (1.41, 1.56) | 1.08 (1.01, 1.15) | 2.47 (2.39, 2.55) | 2.01 (1.93, 2.08) |
| 108-114 | 2.30 (2.24, 2.36) | 1.72 (1.66, 1.77) | 1.60 (1.52, 1.68) | 1.16 (1.09, 1.23) | 2.72 (2.64, 2.81) | 2.05 (1.98, 2.12) |
| 102-108 | 2.45 (2.38, 2.51) | 1.81 (1.75, 1.86) | 1.72 (1.64, 1.81) | 1.16 (1.09, 1.23) | 2.89 (2.80, 2.97) | 2.20 (2.13, 2.27) |
| 96-102 | 2.57 (2.51, 2.64) | 1.91 (1.86, 1.97) | 1.79 (1.70, 1.88) | 1.29 (1.22, 1.36) | 3.05 (2.96, 3.14) | 2.29 (2.22, 2.37) |
| 90-96 | 2.68 (2.62, 2.75) | 1.98 (1.93, 2.04) | 1.94 (1.85, 2.03) | 1.31 (1.24, 1.39) | 3.13 (3.04, 3.22) | 2.39 (2.31, 2.47) |
| 84-90 | 2.88 (2.82, 2.95) | 2.03 (1.97, 2.08) | 2.10 (2.01, 2.20) | 1.41 (1.33, 1.48) | 3.36 (3.27, 3.45) | 2.40 (2.32, 2.48) |
| 78-84 | 3.05 (2.98, 3.12) | 2.11 (2.05, 2.17) | 2.26 (2.16, 2.35) | 1.49 (1.41, 1.57) | 3.53 (3.43, 3.62) | 2.49 (2.41, 2.57) |
| 72-78 | 3.24 (3.17, 3.31) | 2.16 (2.10, 2.22) | 2.42 (2.32, 2.52) | 1.52 (1.44, 1.60) | 3.73 (3.64, 3.83) | 2.55 (2.47, 2.63) |
| 66-72 | 3.45 (3.37, 3.52) | 2.31 (2.25, 2.37) | 2.60 (2.49, 2.70) | 1.58 (1.50, 1.66) | 3.96 (3.86, 4.06) | 2.75 (2.67, 2.83) |
| 60-66 | 3.75 (3.68, 3.83) | 2.43 (2.37, 2.49) | 2.82 (2.71, 2.92) | 1.70 (1.61, 1.78) | 4.32 (4.22, 4.42) | 2.87 (2.79, 2.96) |
| 54-60 | 3.91 (3.83, 3.99) | 2.53 (2.46, 2.59) | 2.89 (2.78, 3.00) | 1.73 (1.65, 1.82) | 4.53 (4.42, 4.63) | 3.01 (2.92, 3.09) |
| 48-54 | 4.09 (4.01, 4.17) | 2.51 (2.45, 2.58) | 3.10 (2.98, 3.21) | 1.84 (1.75, 1.93) | 4.70 (4.59, 4.81) | 2.92 (2.84, 3.01) |
| 42-48 | 4.35 (4.27, 4.43) | 2.61 (2.55, 2.67) | 3.35 (3.24, 3.47) | 1.84 (1.75, 1.92) | 4.95 (4.84, 5.06) | 3.08 (2.99, 3.17) |
| 36-42 | 4.71 (4.62, 4.79) | 2.65 (2.59, 2.71) | 3.73 (3.60, 3.85) | 1.88 (1.79, 1.97) | 5.30 (5.19, 5.42) | 3.12 (3.03, 3.21) |
| 30-36 | 4.93 (4.84, 5.01) | 2.76 (2.69, 2.82) | 3.88 (3.76, 4.01) | 1.91 (1.82, 2.00) | 5.56 (5.45, 5.68) | 3.27 (3.18, 3.36) |
| 24-30 | 5.30 (5.21, 5.39) | 2.84 (2.78, 2.91) | 4.26 (4.13, 4.39) | 2.08 (1.99, 2.17) | 5.93 (5.81, 6.05) | 3.31 (3.22, 3.40) |
| 18-24 | 5.75 (5.66, 5.85) | 2.95 (2.88, 3.01) | 4.55 (4.42, 4.69) | 2.13 (2.04, 2.22) | 6.48 (6.36, 6.61) | 3.44 (3.35, 3.53) |
| 12-18 | 6.34 (6.24, 6.44) | 2.95 (2.88, 3.02) | 5.12 (4.98, 5.26) | 2.10 (2.00, 2.19) | 7.08 (6.95, 7.21) | 3.47 (3.38, 3.57) |
| 6-12 | 7.09 (6.99, 7.19) | 3.00 (2.93, 3.07) | 5.82 (5.67, 5.98) | 2.17 (2.08, 2.27) | 7.86 (7.72, 7.99) | 3.51 (3.41, 3.60) |
| 0-6 | 6.96 (6.86, 7.06) | 2.99 (2.92, 3.06) | 5.73 (5.58, 5.88) | 2.19 (2.10, 2.29) | 7.71 (7.57, 7.84) | 3.47 (3.38, 3.57) |
| Paracetamol | | | | | | |
| 114-120 | 4.32 (4.24, 4.41) | 3.74 (3.66, 3.81) | 2.97 (2.86, 3.08) | 2.59 (2.49, 2.70) | 5.15 (5.03, 5.26) | 4.43 (4.33, 4.53) |
| 108-114 | 4.71 (4.62, 4.79) | 3.96 (3.88, 4.04) | 3.22 (3.11, 3.34) | 2.77 (2.66, 2.87) | 5.61 (5.49, 5.72) | 4.69 (4.58, 4.79) |
| 102-108 | 5.06 (4.97, 5.15) | 4.10 (4.02, 4.17) | 3.48 (3.36, 3.59) | 2.87 (2.76, 2.98) | 6.02 (5.90, 6.14) | 4.84 (4.73, 4.95) |
| 96-102 | 5.36 (5.27, 5.45) | 4.22 (4.14, 4.30) | 3.73 (3.61, 3.86) | 3.01 (2.90, 3.12) | 6.35 (6.23, 6.48) | 4.95 (4.84, 5.06) |
| 90-96 | 5.69 (5.60, 5.78) | 4.37 (4.28, 4.45) | 3.96 (3.83, 4.09) | 3.09 (2.98, 3.20) | 6.74 (6.61, 6.87) | 5.14 (5.03, 5.25) |
| 84-90 | 6.04 (5.94, 6.13) | 4.48 (4.40, 4.56) | 4.25 (4.12, 4.38) | 3.17 (3.05, 3.28) | 7.12 (6.99, 7.25) | 5.28 (5.16, 5.39) |
| 78-84 | 6.38 (6.28, 6.48) | 4.64 (4.56, 4.72) | 4.53 (4.40, 4.67) | 3.29 (3.17, 3.41) | 7.50 (7.36, 7.63) | 5.46 (5.34, 5.57) |
| 72-78 | 6.78 (6.68, 6.88) | 4.75 (4.67, 4.84) | 4.88 (4.74, 5.02) | 3.33 (3.21, 3.44) | 7.93 (7.80, 8.07) | 5.62 (5.50, 5.74) |
| 66-72 | 7.10 (7.00, 7.21) | 4.88 (4.79, 4.96) | 5.14 (5.00, 5.29) | 3.41 (3.29, 3.53) | 8.29 (8.15, 8.43) | 5.76 (5.65, 5.88) |
| 60-66 | 7.59 (7.49, 7.70) | 4.96 (4.88, 5.05) | 5.44 (5.29, 5.59) | 3.50 (3.38, 3.62) | 8.90 (8.75, 9.04) | 5.85 (5.73, 5.97) |
| 54-60 | 7.91 (7.81, 8.02) | 5.09 (5.00, 5.17) | 5.76 (5.61, 5.91) | 3.56 (3.44, 3.68) | 9.22 (9.07, 9.37) | 6.01 (5.89, 6.13) |
| 48-54 | 8.25 (8.14, 8.36) | 5.09 (5.01, 5.18) | 6.06 (5.90, 6.22) | 3.60 (3.48, 3.72) | 9.59 (9.44, 9.74) | 6.00 (5.88, 6.12) |
| 42-48 | 8.69 (8.57, 8.80) | 5.17 (5.08, 5.26) | 6.44 (6.28, 6.60) | 3.63 (3.51, 3.75) | 10.05 (9.90, 10.20) | 6.11 (5.98, 6.23) |
| 36-42 | 9.16 (9.05, 9.28) | 5.24 (5.15, 5.33) | 6.84 (6.68, 7.01) | 3.69 (3.56, 3.81) | 10.57 (10.42, 10.73) | 6.19 (6.07, 6.31) |
| 30-36 | 9.65 (9.53, 9.77) | 5.31 (5.22, 5.40) | 7.35 (7.18, 7.52) | 3.80 (3.68, 3.93) | 11.05 (10.89, 11.21) | 6.23 (6.11, 6.35) |
| 24-30 | 10.25 (10.13, 10.37) | 5.27 (5.18, 5.36) | 7.80 (7.62, 7.97) | 3.74 (3.62, 3.86) | 11.74 (11.58, 11.91) | 6.20 (6.08, 6.33) |
| 18-24 | 11.04 (10.91, 11.16) | 5.34 (5.25, 5.43) | 8.57 (8.38, 8.75) | 3.81 (3.69, 3.94) | 12.54 (12.37, 12.71) | 6.27 (6.15, 6.40) |
| 12-18 | 12.01 (11.88, 12.14) | 5.29 (5.20, 5.38) | 9.41 (9.22, 9.60) | 3.73 (3.61, 3.85) | 13.58 (13.41, 13.75) | 6.23 (6.11, 6.35) |
| 6-12 | 13.82 (13.69, 13.96) | 5.30 (5.21, 5.39) | 11.13 (10.93, 11.34) | 3.74 (3.62, 3.86) | 15.46 (15.27, 15.64) | 6.24 (6.12, 6.37) |
| 0-6 | 15.46 (15.31, 15.60) | 5.27 (5.18, 5.35) | 12.81 (12.59, 13.03) | 3.75 (3.62, 3.87) | 17.07 (16.88, 17.26) | 6.19 (6.07, 6.31) |
| Weak opioids | | | | | | |
| 114-120 | 5.14 (5.05, 5.23) | 3.60 (3.52, 3.67) | 3.60 (3.48, 3.72) | 2.54 (2.44, 2.65) | 6.07 (5.95, 6.20) | 4.24 (4.14, 4.34) |
| 108-114 | 5.17 (5.09, 5.26) | 3.51 (3.44, 3.58) | 3.59 (3.47, 3.71) | 2.54 (2.43, 2.64) | 6.13 (6.01, 6.26) | 4.10 (4.00, 4.20) |
| 102-108 | 5.18 (5.10, 5.27) | 3.43 (3.36, 3.51) | 3.56 (3.43, 3.68) | 2.45 (2.35, 2.55) | 6.17 (6.05, 6.30) | 4.03 (3.93, 4.13) |
| 96-102 | 5.09 (5.00, 5.17) | 3.31 (3.24, 3.38) | 3.55 (3.43, 3.67) | 2.39 (2.30, 2.49) | 6.02 (5.90, 6.14) | 3.87 (3.77, 3.96) |
| 90-96 | 5.05 (4.96, 5.13) | 3.16 (3.09, 3.23) | 3.59 (3.47, 3.71) | 2.24 (2.15, 2.34) | 5.93 (5.81, 6.05) | 3.72 (3.63, 3.82) |
| 84-90 | 5.05 (4.96, 5.13) | 3.15 (3.08, 3.22) | 3.51 (3.39, 3.63) | 2.30 (2.20, 2.40) | 5.98 (5.86, 6.10) | 3.67 (3.57, 3.76) |
| 78-84 | 4.97 (4.89, 5.06) | 3.10 (3.03, 3.17) | 3.54 (3.42, 3.66) | 2.27 (2.18, 2.37) | 5.84 (5.72, 5.96) | 3.60 (3.51, 3.70) |
| 72-78 | 4.94 (4.86, 5.03) | 2.98 (2.91, 3.05) | 3.54 (3.42, 3.66) | 2.09 (2.00, 2.19) | 5.79 (5.68, 5.91) | 3.52 (3.43, 3.61) |
| 66-72 | 4.99 (4.90, 5.08) | 2.99 (2.92, 3.06) | 3.53 (3.41, 3.65) | 2.14 (2.04, 2.23) | 5.87 (5.75, 5.99) | 3.51 (3.41, 3.60) |
| 60-66 | 4.99 (4.91, 5.08) | 2.90 (2.83, 2.96) | 3.67 (3.54, 3.79) | 2.06 (1.97, 2.15) | 5.80 (5.68, 5.92) | 3.40 (3.31, 3.50) |
| 54-60 | 5.02 (4.94, 5.11) | 2.86 (2.79, 2.92) | 3.68 (3.56, 3.81) | 2.08 (1.98, 2.17) | 5.84 (5.72, 5.96) | 3.33 (3.24, 3.42) |
| 48-54 | 5.07 (4.98, 5.16) | 2.79 (2.73, 2.86) | 3.79 (3.66, 3.91) | 2.02 (1.93, 2.11) | 5.85 (5.73, 5.97) | 3.27 (3.18, 3.36) |
| 42-48 | 5.16 (5.08, 5.25) | 2.85 (2.79, 2.92) | 3.95 (3.82, 4.07) | 2.07 (1.98, 2.17) | 5.90 (5.79, 6.02) | 3.33 (3.23, 3.42) |
| 36-42 | 5.32 (5.23, 5.41) | 2.78 (2.71, 2.84) | 4.16 (4.03, 4.29) | 1.99 (1.90, 2.08) | 6.03 (5.91, 6.15) | 3.25 (3.16, 3.34) |
| 30-36 | 5.50 (5.41, 5.60) | 2.80 (2.73, 2.87) | 4.29 (4.15, 4.42) | 2.07 (1.98, 2.16) | 6.24 (6.12, 6.37) | 3.24 (3.15, 3.33) |
| 24-30 | 5.86 (5.76, 5.95) | 2.76 (2.69, 2.82) | 4.57 (4.43, 4.70) | 1.97 (1.88, 2.06) | 6.64 (6.51, 6.76) | 3.23 (3.14, 3.32) |
| 18-24 | 6.35 (6.25, 6.44) | 2.81 (2.74, 2.88) | 4.99 (4.84, 5.13) | 2.02 (1.93, 2.11) | 7.17 (7.04, 7.30) | 3.29 (3.20, 3.38) |
| 12-18 | 7.30 (7.19, 7.40) | 2.78 (2.72, 2.85) | 5.88 (5.73, 6.03) | 2.01 (1.92, 2.10) | 8.16 (8.02, 8.30) | 3.25 (3.16, 3.34) |
| 6-12 | 9.29 (9.18, 9.41) | 2.87 (2.80, 2.94) | 7.94 (7.76, 8.11) | 2.08 (1.99, 2.17) | 10.11 (9.96, 10.27) | 3.35 (3.26, 3.44) |
| 0-6 | 11.25 (11.13, 11.38) | 2.92 (2.86, 2.99) | 9.94 (9.74, 10.13) | 2.11 (2.01, 2.20) | 12.05 (11.89, 12.22) | 3.42 (3.33, 3.51) |
| Strong opioids | | | | | | |
| 114-120 | 2.28 (2.22, 2.34) | 1.70 (1.64, 1.75) | 1.69 (1.61, 1.77) | 1.30 (1.23, 1.37) | 2.63 (2.55, 2.71) | 1.94 (1.87, 2.01) |
| 108-114 | 2.46 (2.40, 2.53) | 1.75 (1.70, 1.80) | 1.84 (1.75, 1.93) | 1.33 (1.26, 1.41) | 2.84 (2.76, 2.93) | 2.00 (1.93, 2.07) |
| 102-108 | 2.67 (2.60, 2.73) | 1.80 (1.75, 1.86) | 2.08 (1.98, 2.17) | 1.41 (1.33, 1.48) | 3.02 (2.94, 3.11) | 2.04 (1.97, 2.11) |
| 96-102 | 2.87 (2.80, 2.93) | 1.89 (1.84, 1.95) | 2.19 (2.09, 2.28) | 1.49 (1.41, 1.56) | 3.28 (3.19, 3.37) | 2.14 (2.07, 2.21) |
| 90-96 | 3.01 (2.95, 3.08) | 1.98 (1.92, 2.03) | 2.29 (2.19, 2.38) | 1.56 (1.48, 1.64) | 3.46 (3.36, 3.55) | 2.23 (2.16, 2.31) |
| 84-90 | 3.20 (3.13, 3.27) | 2.04 (1.98, 2.09) | 2.48 (2.38, 2.58) | 1.54 (1.46, 1.62) | 3.64 (3.54, 3.73) | 2.33 (2.26, 2.41) |
| 78-84 | 3.37 (3.30, 3.44) | 2.11 (2.05, 2.17) | 2.61 (2.51, 2.72) | 1.60 (1.52, 1.68) | 3.83 (3.74, 3.93) | 2.42 (2.34, 2.50) |
| 72-78 | 3.55 (3.48, 3.63) | 2.12 (2.06, 2.18) | 2.68 (2.58, 2.79) | 1.63 (1.55, 1.71) | 4.08 (3.98, 4.19) | 2.42 (2.34, 2.49) |
| 66-72 | 3.71 (3.63, 3.78) | 2.20 (2.15, 2.26) | 2.85 (2.75, 2.96) | 1.66 (1.58, 1.75) | 4.22 (4.12, 4.33) | 2.53 (2.45, 2.61) |
| 60-66 | 3.92 (3.85, 4.00) | 2.24 (2.18, 2.30) | 2.96 (2.85, 3.07) | 1.70 (1.61, 1.78) | 4.51 (4.40, 4.61) | 2.57 (2.49, 2.65) |
| 54-60 | 4.09 (4.01, 4.17) | 2.27 (2.21, 2.33) | 3.10 (2.99, 3.22) | 1.75 (1.66, 1.83) | 4.70 (4.59, 4.80) | 2.59 (2.51, 2.67) |
| 48-54 | 4.36 (4.28, 4.44) | 2.32 (2.26, 2.38) | 3.30 (3.19, 3.42) | 1.77 (1.68, 1.85) | 5.01 (4.90, 5.12) | 2.66 (2.58, 2.74) |
| 42-48 | 4.54 (4.46, 4.63) | 2.35 (2.29, 2.41) | 3.45 (3.33, 3.57) | 1.77 (1.69, 1.86) | 5.21 (5.10, 5.32) | 2.70 (2.62, 2.78) |
| 36-42 | 4.84 (4.76, 4.93) | 2.33 (2.27, 2.39) | 3.70 (3.57, 3.82) | 1.74 (1.65, 1.82) | 5.54 (5.43, 5.66) | 2.69 (2.60, 2.77) |
| 30-36 | 5.05 (4.96, 5.14) | 2.37 (2.31, 2.43) | 3.90 (3.77, 4.03) | 1.73 (1.65, 1.82) | 5.75 (5.63, 5.87) | 2.75 (2.67, 2.84) |
| 24-30 | 5.42 (5.33, 5.51) | 2.40 (2.34, 2.46) | 4.18 (4.05, 4.31) | 1.82 (1.73, 1.90) | 6.18 (6.06, 6.30) | 2.75 (2.67, 2.84) |
| 18-24 | 5.97 (5.87, 6.06) | 2.43 (2.37, 2.49) | 4.64 (4.50, 4.77) | 1.78 (1.70, 1.87) | 6.77 (6.64, 6.90) | 2.83 (2.74, 2.91) |
| 12-18 | 6.83 (6.72, 6.93) | 2.40 (2.34, 2.46) | 5.46 (5.31, 5.61) | 1.78 (1.70, 1.87) | 7.66 (7.52, 7.79) | 2.78 (2.69, 2.86) |
| 6-12 | 8.61 (8.50, 8.73) | 2.44 (2.37, 2.50) | 7.03 (6.86, 7.19) | 1.82 (1.73, 1.91) | 9.58 (9.43, 9.73) | 2.81 (2.73, 2.90) |
| 0-6 | 11.53 (11.40, 11.65) | 2.50 (2.43, 2.56) | 9.74 (9.54, 9.93) | 1.84 (1.75, 1.92) | 12.61 (12.44, 12.78) | 2.90 (2.81, 2.98) |
|  | Antidepressants | | | | | |
| 114-120 | 8.77 (8.66, 8.89) | 7.46 (7.36, 7.57) | 4.86 (4.72, 5.00) | 4.47 (4.33, 4.60) | 11.15 (10.99, 11.31) | 9.28 (9.14, 9.43) |
| 108-114 | 9.01 (8.89, 9.12) | 7.57 (7.46, 7.67) | 5.05 (4.90, 5.19) | 4.50 (4.36, 4.63) | 11.41 (11.25, 11.58) | 9.43 (9.28, 9.58) |
| 102-108 | 9.12 (9.00, 9.23) | 7.59 (7.49, 7.70) | 5.06 (4.92, 5.20) | 4.58 (4.45, 4.72) | 11.58 (11.42, 11.74) | 9.42 (9.27, 9.57) |
| 96-102 | 9.29 (9.17, 9.41) | 7.64 (7.54, 7.75) | 5.22 (5.08, 5.37) | 4.57 (4.44, 4.71) | 11.76 (11.60, 11.92) | 9.51 (9.36, 9.66) |
| 90-96 | 9.45 (9.33, 9.57) | 7.69 (7.58, 7.80) | 5.36 (5.22, 5.51) | 4.68 (4.54, 4.82) | 11.93 (11.77, 12.09) | 9.52 (9.37, 9.66) |
| 84-90 | 9.55 (9.43, 9.67) | 7.69 (7.58, 7.80) | 5.48 (5.33, 5.63) | 4.66 (4.53, 4.80) | 12.02 (11.85, 12.18) | 9.53 (9.38, 9.67) |
| 78-84 | 9.68 (9.56, 9.80) | 7.79 (7.68, 7.90) | 5.57 (5.42, 5.72) | 4.78 (4.64, 4.92) | 12.18 (12.01, 12.34) | 9.62 (9.47, 9.77) |
| 72-78 | 9.82 (9.70, 9.93) | 7.78 (7.68, 7.89) | 5.61 (5.46, 5.75) | 4.72 (4.58, 4.86) | 12.37 (12.20, 12.54) | 9.64 (9.49, 9.79) |
| 66-72 | 9.93 (9.81, 10.05) | 7.80 (7.69, 7.90) | 5.77 (5.62, 5.92) | 4.73 (4.59, 4.87) | 12.45 (12.29, 12.62) | 9.66 (9.51, 9.81) |
| 60-66 | 10.09 (9.97, 10.21) | 7.87 (7.76, 7.97) | 5.91 (5.76, 6.07) | 4.80 (4.66, 4.93) | 12.62 (12.45, 12.79) | 9.73 (9.58, 9.88) |
| 54-60 | 10.29 (10.17, 10.41) | 7.84 (7.73, 7.94) | 6.04 (5.88, 6.19) | 4.78 (4.65, 4.92) | 12.87 (12.70, 13.04) | 9.69 (9.54, 9.84) |
| 48-54 | 10.45 (10.33, 10.58) | 7.86 (7.76, 7.97) | 6.16 (6.00, 6.31) | 4.81 (4.67, 4.95) | 13.06 (12.89, 13.24) | 9.71 (9.56, 9.86) |
| 42-48 | 10.60 (10.48, 10.72) | 7.94 (7.84, 8.05) | 6.28 (6.12, 6.44) | 4.84 (4.70, 4.98) | 13.22 (13.05, 13.39) | 9.83 (9.68, 9.98) |
| 36-42 | 10.77 (10.64, 10.89) | 7.90 (7.79, 8.01) | 6.41 (6.25, 6.57) | 4.79 (4.65, 4.93) | 13.41 (13.24, 13.58) | 9.79 (9.64, 9.94) |
| 30-36 | 10.93 (10.81, 11.06) | 7.96 (7.86, 8.07) | 6.48 (6.32, 6.64) | 4.82 (4.68, 4.96) | 13.63 (13.46, 13.81) | 9.87 (9.72, 10.03) |
| 24-30 | 11.28 (11.15, 11.40) | 7.96 (7.86, 8.07) | 6.76 (6.60, 6.93) | 4.81 (4.67, 4.95) | 14.02 (13.84, 14.19) | 9.88 (9.73, 10.03) |
| 18-24 | 11.55 (11.42, 11.67) | 7.95 (7.84, 8.06) | 6.92 (6.75, 7.08) | 4.80 (4.67, 4.94) | 14.36 (14.18, 14.54) | 9.86 (9.71, 10.01) |
| 12-18 | 11.96 (11.83, 12.09) | 7.86 (7.75, 7.97) | 7.31 (7.14, 7.48) | 4.76 (4.62, 4.90) | 14.78 (14.60, 14.96) | 9.74 (9.59, 9.89) |
| 6-12 | 12.55 (12.41, 12.68) | 7.85 (7.74, 7.96) | 7.88 (7.70, 8.05) | 4.79 (4.65, 4.92) | 15.38 (15.20, 15.57) | 9.71 (9.56, 9.86) |
| 0-6 | 12.86 (12.72, 12.99) | 7.82 (7.72, 7.93) | 8.23 (8.05, 8.41) | 4.76 (4.62, 4.90) | 15.66 (15.48, 15.85) | 9.68 (9.53, 9.83) |
| Antiepileptics | | | | | | |
| 114-120 | 1.62 (1.57, 1.68) | 1.53 (1.48, 1.58) | 1.42 (1.35, 1.50) | 1.45 (1.37, 1.53) | 1.75 (1.68, 1.81) | 1.58 (1.52, 1.64) |
| 108-114 | 1.70 (1.64, 1.75) | 1.58 (1.53, 1.63) | 1.48 (1.40, 1.55) | 1.48 (1.40, 1.55) | 1.83 (1.76, 1.90) | 1.64 (1.58, 1.71) |
| 102-108 | 1.79 (1.73, 1.84) | 1.60 (1.55, 1.65) | 1.54 (1.46, 1.62) | 1.46 (1.39, 1.54) | 1.94 (1.87, 2.01) | 1.68 (1.62, 1.75) |
| 96-102 | 1.87 (1.82, 1.93) | 1.68 (1.63, 1.73) | 1.62 (1.53, 1.70) | 1.56 (1.48, 1.64) | 2.03 (1.96, 2.10) | 1.76 (1.69, 1.82) |
| 90-96 | 1.93 (1.88, 1.99) | 1.69 (1.64, 1.74) | 1.65 (1.57, 1.74) | 1.58 (1.50, 1.67) | 2.10 (2.03, 2.17) | 1.75 (1.68, 1.82) |
| 84-90 | 2.03 (1.97, 2.08) | 1.75 (1.70, 1.80) | 1.72 (1.64, 1.80) | 1.64 (1.56, 1.72) | 2.21 (2.14, 2.29) | 1.82 (1.75, 1.89) |
| 78-84 | 2.16 (2.10, 2.22) | 1.80 (1.75, 1.85) | 1.83 (1.74, 1.91) | 1.63 (1.55, 1.71) | 2.37 (2.29, 2.44) | 1.91 (1.84, 1.97) |
| 72-78 | 2.27 (2.21, 2.33) | 1.86 (1.80, 1.91) | 1.90 (1.81, 1.98) | 1.70 (1.62, 1.79) | 2.50 (2.42, 2.58) | 1.95 (1.88, 2.02) |
| 66-72 | 2.39 (2.33, 2.46) | 1.88 (1.83, 1.94) | 2.01 (1.92, 2.11) | 1.70 (1.61, 1.78) | 2.62 (2.54, 2.71) | 2.00 (1.93, 2.07) |
| 60-66 | 2.53 (2.47, 2.60) | 1.93 (1.87, 1.98) | 2.14 (2.05, 2.24) | 1.71 (1.63, 1.80) | 2.77 (2.69, 2.85) | 2.06 (1.99, 2.13) |
| 54-60 | 2.65 (2.59, 2.72) | 1.97 (1.92, 2.03) | 2.25 (2.15, 2.34) | 1.74 (1.66, 1.83) | 2.90 (2.82, 2.99) | 2.11 (2.04, 2.19) |
| 48-54 | 2.81 (2.75, 2.88) | 2.03 (1.97, 2.08) | 2.36 (2.26, 2.46) | 1.80 (1.72, 1.89) | 3.09 (3.00, 3.17) | 2.16 (2.09, 2.23) |
| 42-48 | 2.99 (2.92, 3.06) | 2.08 (2.02, 2.13) | 2.49 (2.39, 2.59) | 1.85 (1.76, 1.94) | 3.30 (3.21, 3.39) | 2.22 (2.14, 2.29) |
| 36-42 | 3.14 (3.07, 3.21) | 2.14 (2.08, 2.19) | 2.59 (2.48, 2.69) | 1.89 (1.80, 1.98) | 3.47 (3.38, 3.57) | 2.29 (2.21, 2.36) |
| 30-36 | 3.35 (3.28, 3.42) | 2.16 (2.10, 2.21) | 2.76 (2.65, 2.86) | 1.87 (1.78, 1.96) | 3.71 (3.61, 3.80) | 2.33 (2.25, 2.40) |
| 24-30 | 3.58 (3.50, 3.65) | 2.24 (2.18, 2.30) | 2.95 (2.84, 3.06) | 1.94 (1.85, 2.03) | 3.96 (3.86, 4.06) | 2.42 (2.34, 2.49) |
| 18-24 | 3.83 (3.75, 3.90) | 2.27 (2.21, 2.33) | 3.10 (2.99, 3.22) | 1.94 (1.85, 2.03) | 4.27 (4.16, 4.37) | 2.47 (2.39, 2.55) |
| 12-18 | 4.18 (4.10, 4.26) | 2.31 (2.25, 2.37) | 3.40 (3.29, 3.52) | 1.98 (1.89, 2.07) | 4.66 (4.55, 4.76) | 2.51 (2.43, 2.59) |
| 6-12 | 4.70 (4.61, 4.78) | 2.40 (2.34, 2.46) | 3.81 (3.69, 3.94) | 2.03 (1.93, 2.12) | 5.23 (5.12, 5.35) | 2.63 (2.54, 2.71) |
| 0-6 | 5.05 (4.96, 5.14) | 2.42 (2.36, 2.48) | 4.15 (4.02, 4.28) | 2.06 (1.96, 2.15) | 5.59 (5.47, 5.71) | 2.64 (2.56, 2.72) |
| Bisphosphonates | | | | | | |
| 114-120 | 1.21 (1.17, 1.25) | 1.26 (1.21, 1.30) | 0.27 (0.24, 0.31) | 0.33 (0.29, 0.37) | 1.78 (1.71, 1.85) | 1.82 (1.75, 1.89) |
| 108-114 | 1.31 (1.26, 1.35) | 1.35 (1.30, 1.39) | 0.29 (0.25, 0.32) | 0.36 (0.32, 0.40) | 1.93 (1.86, 2.00) | 1.95 (1.88, 2.02) |
| 102-108 | 1.41 (1.36, 1.46) | 1.44 (1.39, 1.48) | 0.33 (0.29, 0.36) | 0.39 (0.35, 0.44) | 2.07 (2.00, 2.14) | 2.07 (2.00, 2.14) |
| 96-102 | 1.52 (1.47, 1.56) | 1.48 (1.44, 1.53) | 0.35 (0.31, 0.39) | 0.41 (0.37, 0.45) | 2.23 (2.15, 2.30) | 2.14 (2.07, 2.21) |
| 90-96 | 1.64 (1.59, 1.69) | 1.56 (1.51, 1.61) | 0.38 (0.34, 0.42) | 0.42 (0.38, 0.46) | 2.41 (2.33, 2.49) | 2.25 (2.17, 2.32) |
| 84-90 | 1.74 (1.69, 1.79) | 1.64 (1.59, 1.69) | 0.39 (0.35, 0.43) | 0.45 (0.40, 0.49) | 2.56 (2.48, 2.64) | 2.37 (2.29, 2.45) |
| 78-84 | 1.85 (1.80, 1.90) | 1.69 (1.64, 1.74) | 0.42 (0.38, 0.46) | 0.47 (0.42, 0.51) | 2.72 (2.64, 2.80) | 2.44 (2.36, 2.52) |
| 72-78 | 1.95 (1.90, 2.01) | 1.75 (1.70, 1.80) | 0.44 (0.39, 0.48) | 0.47 (0.43, 0.52) | 2.87 (2.79, 2.96) | 2.53 (2.45, 2.61) |
| 66-72 | 2.04 (1.99, 2.10) | 1.81 (1.75, 1.86) | 0.46 (0.42, 0.51) | 0.47 (0.43, 0.52) | 3.00 (2.92, 3.09) | 2.61 (2.53, 2.70) |
| 60-66 | 2.15 (2.09, 2.21) | 1.88 (1.82, 1.93) | 0.51 (0.47, 0.56) | 0.48 (0.43, 0.52) | 3.14 (3.05, 3.23) | 2.72 (2.64, 2.81) |
| 54-60 | 2.25 (2.19, 2.31) | 1.89 (1.84, 1.95) | 0.54 (0.49, 0.58) | 0.49 (0.45, 0.54) | 3.29 (3.20, 3.38) | 2.74 (2.66, 2.83) |
| 48-54 | 2.34 (2.28, 2.40) | 1.92 (1.87, 1.98) | 0.56 (0.52, 0.61) | 0.51 (0.46, 0.56) | 3.41 (3.32, 3.50) | 2.78 (2.70, 2.87) |
| 42-48 | 2.45 (2.39, 2.51) | 1.96 (1.90, 2.01) | 0.63 (0.57, 0.68) | 0.53 (0.48, 0.58) | 3.55 (3.46, 3.65) | 2.82 (2.74, 2.90) |
| 36-42 | 2.54 (2.47, 2.60) | 1.98 (1.92, 2.03) | 0.65 (0.60, 0.70) | 0.54 (0.50, 0.59) | 3.68 (3.59, 3.78) | 2.85 (2.77, 2.93) |
| 30-36 | 2.64 (2.58, 2.70) | 2.02 (1.96, 2.08) | 0.71 (0.66, 0.77) | 0.54 (0.49, 0.58) | 3.81 (3.71, 3.91) | 2.92 (2.84, 3.01) |
| 24-30 | 2.73 (2.67, 2.80) | 1.98 (1.93, 2.04) | 0.78 (0.72, 0.83) | 0.55 (0.50, 0.59) | 3.92 (3.82, 4.02) | 2.85 (2.77, 2.94) |
| 18-24 | 2.85 (2.78, 2.92) | 2.03 (1.97, 2.09) | 0.85 (0.79, 0.91) | 0.55 (0.50, 0.59) | 4.07 (3.97, 4.17) | 2.93 (2.85, 3.02) |
| 12-18 | 2.98 (2.91, 3.05) | 2.05 (2.00, 2.11) | 0.93 (0.87, 1.00) | 0.57 (0.52, 0.62) | 4.22 (4.12, 4.32) | 2.95 (2.87, 3.04) |
| 6-12 | 3.09 (3.02, 3.16) | 2.05 (2.00, 2.11) | 1.00 (0.93, 1.06) | 0.57 (0.52, 0.62) | 4.36 (4.26, 4.47) | 2.95 (2.87, 3.04) |
| 0-6 | 3.26 (3.19, 3.33) | 2.07 (2.02, 2.13) | 1.10 (1.03, 1.17) | 0.57 (0.52, 0.62) | 4.57 (4.47, 4.68) | 2.98 (2.90, 3.07) |
| All corticosteroids | | | | | | |
| 114-120 | 15.84 (15.70, 15.99) | 14.35 (14.21, 14.49) | 13.69 (13.47, 13.91) | 12.56 (12.35, 12.78) | 17.15 (16.96, 17.34) | 15.43 (15.25, 15.61) |
| 108-114 | 16.41 (16.27, 16.56) | 14.18 (14.04, 14.32) | 14.18 (13.96, 14.41) | 12.34 (12.12, 12.55) | 17.77 (17.57, 17.96) | 15.29 (15.11, 15.48) |
| 102-108 | 16.62 (16.47, 16.77) | 14.43 (14.29, 14.57) | 14.33 (14.10, 14.55) | 12.65 (12.44, 12.87) | 18.01 (17.82, 18.21) | 15.52 (15.33, 15.70) |
| 96-102 | 16.67 (16.52, 16.82) | 14.17 (14.03, 14.31) | 14.24 (14.02, 14.47) | 12.38 (12.17, 12.60) | 18.14 (17.95, 18.34) | 15.25 (15.07, 15.43) |
| 90-96 | 16.83 (16.68, 16.98) | 14.57 (14.43, 14.71) | 14.47 (14.24, 14.69) | 12.80 (12.59, 13.02) | 18.26 (18.06, 18.46) | 15.65 (15.46, 15.83) |
| 84-90 | 17.08 (16.93, 17.23) | 14.25 (14.11, 14.39) | 14.80 (14.57, 15.03) | 12.45 (12.24, 12.67) | 18.46 (18.27, 18.66) | 15.34 (15.16, 15.52) |
| 78-84 | 17.12 (16.97, 17.27) | 14.62 (14.48, 14.76) | 15.07 (14.84, 15.31) | 12.78 (12.57, 13.00) | 18.36 (18.16, 18.55) | 15.73 (15.55, 15.92) |
| 72-78 | 17.26 (17.11, 17.41) | 14.16 (14.02, 14.30) | 14.92 (14.68, 15.15) | 12.36 (12.15, 12.58) | 18.68 (18.48, 18.88) | 15.25 (15.06, 15.43) |
| 66-72 | 17.48 (17.33, 17.64) | 14.64 (14.50, 14.78) | 15.38 (15.15, 15.62) | 12.87 (12.66, 13.09) | 18.76 (18.56, 18.96) | 15.71 (15.52, 15.89) |
| 60-66 | 17.59 (17.44, 17.74) | 13.95 (13.81, 14.09) | 15.55 (15.31, 15.78) | 12.09 (11.88, 12.30) | 18.83 (18.63, 19.03) | 15.08 (14.90, 15.26) |
| 54-60 | 17.73 (17.58, 17.88) | 14.37 (14.23, 14.51) | 15.63 (15.40, 15.87) | 12.64 (12.43, 12.86) | 19.00 (18.80, 19.20) | 15.42 (15.23, 15.60) |
| 48-54 | 17.84 (17.69, 17.99) | 13.96 (13.82, 14.10) | 15.88 (15.64, 16.12) | 12.22 (12.01, 12.43) | 19.03 (18.83, 19.23) | 15.02 (14.83, 15.20) |
| 42-48 | 17.91 (17.76, 18.07) | 14.39 (14.25, 14.53) | 15.92 (15.68, 16.15) | 12.69 (12.47, 12.90) | 19.13 (18.93, 19.32) | 15.43 (15.25, 15.61) |
| 36-42 | 17.91 (17.75, 18.06) | 13.83 (13.69, 13.97) | 16.08 (15.84, 16.32) | 12.23 (12.02, 12.44) | 19.01 (18.81, 19.21) | 14.81 (14.63, 14.99) |
| 30-36 | 18.21 (18.06, 18.37) | 14.17 (14.03, 14.31) | 16.42 (16.18, 16.66) | 12.44 (12.22, 12.65) | 19.30 (19.10, 19.50) | 15.22 (15.04, 15.40) |
| 24-30 | 18.33 (18.18, 18.48) | 13.57 (13.43, 13.70) | 16.53 (16.29, 16.77) | 11.84 (11.63, 12.05) | 19.42 (19.22, 19.62) | 14.62 (14.44, 14.80) |
| 18-24 | 18.46 (18.31, 18.62) | 13.89 (13.75, 14.03) | 16.78 (16.53, 17.02) | 12.25 (12.03, 12.46) | 19.48 (19.28, 19.68) | 14.89 (14.71, 15.07) |
| 12-18 | 18.65 (18.49, 18.80) | 13.39 (13.25, 13.52) | 16.96 (16.71, 17.20) | 11.69 (11.48, 11.90) | 19.67 (19.47, 19.87) | 14.42 (14.24, 14.60) |
| 6-12 | 18.82 (18.66, 18.97) | 13.55 (13.42, 13.69) | 17.14 (16.90, 17.39) | 11.98 (11.77, 12.19) | 19.84 (19.63, 20.04) | 14.51 (14.33, 14.69) |
| 0-6 | 18.25 (18.10, 18.40) | 12.99 (12.86, 13.13) | 17.02 (16.78, 17.26) | 11.37 (11.16, 11.57) | 19.00 (18.80, 19.19) | 13.98 (13.81, 14.16) |
| Systemic corticosteroids | | | | | | |
| 114-120 | 2.53 (2.46, 2.59) | 2.39 (2.33, 2.46) | 2.02 (1.93, 2.11) | 1.93 (1.84, 2.02) | 2.83 (2.75, 2.92) | 2.68 (2.59, 2.76) |
| 108-114 | 2.68 (2.62, 2.75) | 2.37 (2.31, 2.43) | 2.05 (1.96, 2.14) | 1.91 (1.82, 2.00) | 3.07 (2.98, 3.16) | 2.65 (2.57, 2.74) |
| 102-108 | 2.74 (2.67, 2.80) | 2.43 (2.37, 2.49) | 2.13 (2.04, 2.23) | 1.97 (1.88, 2.06) | 3.11 (3.02, 3.20) | 2.72 (2.63, 2.80) |
| 96-102 | 2.80 (2.73, 2.86) | 2.41 (2.35, 2.47) | 2.11 (2.01, 2.20) | 1.97 (1.88, 2.06) | 3.21 (3.12, 3.30) | 2.68 (2.60, 2.76) |
| 90-96 | 2.92 (2.85, 2.98) | 2.53 (2.47, 2.59) | 2.22 (2.12, 2.31) | 2.07 (1.98, 2.16) | 3.34 (3.25, 3.43) | 2.81 (2.73, 2.89) |
| 84-90 | 2.92 (2.85, 2.98) | 2.46 (2.40, 2.52) | 2.19 (2.09, 2.28) | 2.04 (1.95, 2.14) | 3.36 (3.27, 3.45) | 2.71 (2.63, 2.79) |
| 78-84 | 3.08 (3.01, 3.15) | 2.65 (2.59, 2.72) | 2.39 (2.29, 2.49) | 2.14 (2.05, 2.24) | 3.50 (3.41, 3.59) | 2.96 (2.88, 3.05) |
| 72-78 | 3.15 (3.08, 3.22) | 2.54 (2.47, 2.60) | 2.41 (2.31, 2.51) | 2.08 (1.99, 2.18) | 3.61 (3.51, 3.70) | 2.81 (2.73, 2.90) |
| 66-72 | 3.29 (3.22, 3.37) | 2.73 (2.67, 2.80) | 2.56 (2.46, 2.66) | 2.18 (2.09, 2.28) | 3.74 (3.64, 3.84) | 3.07 (2.98, 3.15) |
| 60-66 | 3.39 (3.32, 3.46) | 2.57 (2.51, 2.63) | 2.59 (2.48, 2.69) | 2.04 (1.95, 2.13) | 3.88 (3.78, 3.98) | 2.89 (2.81, 2.98) |
| 54-60 | 3.53 (3.45, 3.60) | 2.77 (2.70, 2.83) | 2.69 (2.58, 2.79) | 2.26 (2.17, 2.36) | 4.04 (3.94, 4.14) | 3.07 (2.99, 3.16) |
| 48-54 | 3.64 (3.56, 3.71) | 2.70 (2.63, 2.76) | 2.89 (2.78, 3.00) | 2.16 (2.07, 2.26) | 4.09 (3.99, 4.19) | 3.02 (2.93, 3.10) |
| 42-48 | 3.79 (3.72, 3.87) | 2.86 (2.79, 2.92) | 3.08 (2.97, 3.20) | 2.35 (2.25, 2.45) | 4.22 (4.12, 4.33) | 3.16 (3.08, 3.25) |
| 36-42 | 3.93 (3.85, 4.01) | 2.71 (2.65, 2.78) | 3.18 (3.07, 3.30) | 2.29 (2.20, 2.39) | 4.38 (4.28, 4.48) | 2.97 (2.88, 3.05) |
| 30-36 | 4.22 (4.14, 4.30) | 2.89 (2.82, 2.95) | 3.43 (3.31, 3.55) | 2.38 (2.28, 2.48) | 4.70 (4.59, 4.80) | 3.19 (3.10, 3.28) |
| 24-30 | 4.43 (4.35, 4.51) | 2.78 (2.71, 2.84) | 3.55 (3.42, 3.67) | 2.23 (2.13, 2.33) | 4.97 (4.86, 5.08) | 3.11 (3.02, 3.20) |
| 18-24 | 4.73 (4.65, 4.82) | 2.96 (2.89, 3.03) | 3.87 (3.74, 3.99) | 2.41 (2.31, 2.51) | 5.26 (5.15, 5.37) | 3.29 (3.20, 3.38) |
| 12-18 | 5.13 (5.04, 5.22) | 2.85 (2.79, 2.92) | 4.15 (4.02, 4.28) | 2.29 (2.19, 2.39) | 5.72 (5.61, 5.84) | 3.20 (3.11, 3.29) |
| 6-12 | 5.51 (5.42, 5.60) | 2.94 (2.87, 3.01) | 4.60 (4.46, 4.74) | 2.40 (2.30, 2.50) | 6.06 (5.94, 6.18) | 3.27 (3.18, 3.36) |
| 0-6 | 4.51 (4.42, 4.59) | 2.79 (2.73, 2.86) | 3.86 (3.73, 3.98) | 2.26 (2.17, 2.36) | 4.90 (4.79, 5.01) | 3.11 (3.02, 3.20) |
| GP visits | | | | | | |
| 114-120 | 2.52 (2.46, 2.58) | 2.21 (2.15, 2.26) | 2.19 (2.09, 2.28) | 1.83 (1.74, 1.91) | 2.72 (2.64, 2.80) | 2.44 (2.36, 2.52) |
| 108-114 | 2.55 (2.49, 2.62) | 2.24 (2.18, 2.30) | 2.19 (2.09, 2.28) | 1.86 (1.77, 1.95) | 2.77 (2.69, 2.86) | 2.48 (2.40, 2.55) |
| 102-108 | 2.47 (2.40, 2.53) | 2.09 (2.03, 2.14) | 2.13 (2.04, 2.23) | 1.76 (1.67, 1.84) | 2.67 (2.59, 2.75) | 2.29 (2.21, 2.36) |
| 96-102 | 2.42 (2.36, 2.48) | 2.12 (2.07, 2.18) | 2.10 (2.01, 2.20) | 1.83 (1.74, 1.92) | 2.61 (2.53, 2.69) | 2.30 (2.22, 2.38) |
| 90-96 | 2.33 (2.27, 2.39) | 1.97 (1.91, 2.02) | 2.06 (1.97, 2.15) | 1.66 (1.58, 1.75) | 2.49 (2.41, 2.57) | 2.15 (2.08, 2.22) |
| 84-90 | 2.27 (2.21, 2.33) | 1.96 (1.90, 2.01) | 2.02 (1.93, 2.11) | 1.66 (1.58, 1.75) | 2.42 (2.34, 2.50) | 2.14 (2.06, 2.21) |
| 78-84 | 2.17 (2.12, 2.23) | 1.86 (1.80, 1.91) | 2.00 (1.91, 2.09) | 1.59 (1.51, 1.68) | 2.28 (2.20, 2.36) | 2.02 (1.95, 2.09) |
| 72-78 | 2.11 (2.06, 2.17) | 1.80 (1.75, 1.86) | 1.93 (1.84, 2.02) | 1.61 (1.53, 1.69) | 2.22 (2.15, 2.30) | 1.92 (1.85, 1.99) |
| 66-72 | 2.06 (2.00, 2.11) | 1.74 (1.69, 1.79) | 1.93 (1.84, 2.02) | 1.53 (1.45, 1.61) | 2.13 (2.06, 2.21) | 1.87 (1.80, 1.94) |
| 60-66 | 2.03 (1.98, 2.09) | 1.72 (1.67, 1.77) | 1.94 (1.85, 2.03) | 1.53 (1.45, 1.61) | 2.09 (2.02, 2.16) | 1.83 (1.77, 1.90) |
| 54-60 | 2.00 (1.94, 2.06) | 1.67 (1.62, 1.72) | 1.91 (1.82, 2.00) | 1.47 (1.39, 1.55) | 2.05 (1.98, 2.12) | 1.79 (1.72, 1.85) |
| 48-54 | 1.95 (1.90, 2.01) | 1.62 (1.57, 1.67) | 1.86 (1.77, 1.94) | 1.46 (1.39, 1.54) | 2.01 (1.94, 2.08) | 1.71 (1.64, 1.77) |
| 42-48 | 1.95 (1.89, 2.00) | 1.56 (1.51, 1.61) | 1.90 (1.81, 1.99) | 1.43 (1.35, 1.50) | 1.98 (1.91, 2.05) | 1.64 (1.57, 1.70) |
| 36-42 | 1.87 (1.81, 1.92) | 1.52 (1.47, 1.57) | 1.82 (1.73, 1.90) | 1.37 (1.30, 1.45) | 1.90 (1.83, 1.97) | 1.61 (1.54, 1.67) |
| 30-36 | 1.80 (1.75, 1.86) | 1.43 (1.38, 1.47) | 1.71 (1.63, 1.79) | 1.32 (1.25, 1.39) | 1.86 (1.79, 1.93) | 1.49 (1.43, 1.55) |
| 24-30 | 1.81 (1.76, 1.86) | 1.39 (1.34, 1.44) | 1.75 (1.67, 1.84) | 1.26 (1.19, 1.33) | 1.84 (1.77, 1.91) | 1.47 (1.41, 1.53) |
| 18-24 | 1.76 (1.70, 1.81) | 1.33 (1.28, 1.37) | 1.72 (1.63, 1.80) | 1.24 (1.17, 1.31) | 1.78 (1.71, 1.85) | 1.38 (1.32, 1.44) |
| 12-18 | 1.71 (1.66, 1.76) | 1.25 (1.21, 1.30) | 1.71 (1.62, 1.79) | 1.15 (1.09, 1.22) | 1.71 (1.65, 1.78) | 1.31 (1.25, 1.37) |
| 6-12 | 1.69 (1.64, 1.74) | 1.18 (1.14, 1.23) | 1.68 (1.60, 1.77) | 1.11 (1.04, 1.18) | 1.69 (1.62, 1.75) | 1.23 (1.17, 1.28) |
| 0-6 | 1.70 (1.65, 1.75) | 1.09 (1.05, 1.14) | 1.72 (1.63, 1.80) | 1.04 (0.97, 1.10) | 1.69 (1.62, 1.75) | 1.13 (1.08, 1.18) |
| Referrals for physiotherapy | | | | | | |
| 114-120 | 1.25 (1.21, 1.30) | 0.82 (0.78, 0.86) | 1.03 (0.97, 1.10) | 0.61 (0.56, 0.66) | 1.39 (1.33, 1.45) | 0.95 (0.90, 1.00) |
| 108-114 | 1.29 (1.25, 1.34) | 0.83 (0.79, 0.86) | 1.04 (0.97, 1.10) | 0.62 (0.57, 0.67) | 1.44 (1.38, 1.51) | 0.95 (0.90, 1.00) |
| 102-108 | 1.33 (1.29, 1.38) | 0.88 (0.84, 0.91) | 1.06 (0.99, 1.13) | 0.63 (0.57, 0.68) | 1.50 (1.43, 1.56) | 1.03 (0.98, 1.08) |
| 96-102 | 1.30 (1.25, 1.35) | 0.87 (0.83, 0.90) | 0.99 (0.93, 1.06) | 0.64 (0.59, 0.70) | 1.49 (1.42, 1.55) | 1.00 (0.95, 1.05) |
| 90-96 | 1.43 (1.38, 1.47) | 0.93 (0.90, 0.97) | 1.14 (1.07, 1.21) | 0.71 (0.65, 0.76) | 1.60 (1.54, 1.66) | 1.07 (1.02, 1.12) |
| 84-90 | 1.40 (1.35, 1.44) | 0.87 (0.83, 0.90) | 1.14 (1.07, 1.21) | 0.66 (0.61, 0.72) | 1.55 (1.49, 1.61) | 0.99 (0.94, 1.04) |
| 78-84 | 1.44 (1.39, 1.48) | 0.91 (0.87, 0.94) | 1.16 (1.09, 1.23) | 0.69 (0.63, 0.74) | 1.60 (1.54, 1.67) | 1.04 (0.99, 1.09) |
| 72-78 | 1.46 (1.42, 1.51) | 0.88 (0.84, 0.91) | 1.14 (1.07, 1.21) | 0.68 (0.63, 0.74) | 1.66 (1.59, 1.72) | 0.99 (0.94, 1.04) |
| 66-72 | 1.54 (1.49, 1.59) | 0.98 (0.94, 1.02) | 1.24 (1.17, 1.31) | 0.64 (0.59, 0.69) | 1.72 (1.66, 1.79) | 1.18 (1.13, 1.24) |
| 60-66 | 1.55 (1.50, 1.60) | 0.89 (0.85, 0.92) | 1.23 (1.16, 1.31) | 0.69 (0.64, 0.75) | 1.75 (1.68, 1.81) | 1.00 (0.95, 1.05) |
| 54-60 | 1.65 (1.60, 1.70) | 0.97 (0.93, 1.01) | 1.32 (1.25, 1.40) | 0.73 (0.67, 0.78) | 1.85 (1.78, 1.92) | 1.11 (1.06, 1.17) |
| 48-54 | 1.66 (1.61, 1.71) | 0.96 (0.92, 1.00) | 1.31 (1.23, 1.38) | 0.73 (0.68, 0.79) | 1.87 (1.80, 1.94) | 1.10 (1.04, 1.15) |
| 42-48 | 1.82 (1.77, 1.87) | 0.98 (0.94, 1.02) | 1.53 (1.45, 1.61) | 0.73 (0.67, 0.78) | 2.00 (1.92, 2.07) | 1.13 (1.08, 1.19) |
| 36-42 | 1.91 (1.85, 1.96) | 0.99 (0.95, 1.03) | 1.55 (1.47, 1.63) | 0.77 (0.71, 0.83) | 2.12 (2.05, 2.20) | 1.12 (1.07, 1.18) |
| 30-36 | 2.01 (1.96, 2.07) | 1.09 (1.05, 1.13) | 1.69 (1.61, 1.77) | 0.81 (0.75, 0.87) | 2.21 (2.14, 2.29) | 1.26 (1.20, 1.32) |
| 24-30 | 2.25 (2.19, 2.31) | 1.00 (0.96, 1.04) | 1.90 (1.81, 1.99) | 0.73 (0.67, 0.78) | 2.46 (2.38, 2.54) | 1.17 (1.12, 1.22) |
| 18-24 | 2.57 (2.51, 2.63) | 1.06 (1.02, 1.10) | 2.11 (2.01, 2.20) | 0.76 (0.70, 0.82) | 2.85 (2.77, 2.94) | 1.24 (1.19, 1.30) |
| 12-18 | 3.17 (3.10, 3.24) | 1.06 (1.02, 1.10) | 2.66 (2.56, 2.77) | 0.77 (0.71, 0.82) | 3.49 (3.39, 3.58) | 1.23 (1.18, 1.29) |
| 6-12 | 3.96 (3.88, 4.03) | 1.08 (1.04, 1.12) | 3.55 (3.43, 3.67) | 0.80 (0.74, 0.86) | 4.20 (4.10, 4.31) | 1.25 (1.19, 1.30) |
| 0-6 | 2.77 (2.71, 2.84) | 1.00 (0.96, 1.04) | 2.51 (2.41, 2.61) | 0.76 (0.70, 0.81) | 2.93 (2.85, 3.02) | 1.15 (1.10, 1.21) |
| Referrals for imaging | | | | | | |
| 114-120 | 0.42 (0.39, 0.44) | 0.21 (0.19, 0.23) | 0.12 (0.09, 0.14) | 0.31 (0.28, 0.35) | 0.48 (0.45, 0.52) | 0.27 (0.24, 0.29) |
| 108-114 | 0.45 (0.42, 0.47) | 0.19 (0.18, 0.21) | 0.11 (0.09, 0.13) | 0.33 (0.29, 0.37) | 0.52 (0.48, 0.55) | 0.24 (0.22, 0.27) |
| 102-108 | 0.49 (0.46, 0.52) | 0.22 (0.20, 0.24) | 0.12 (0.10, 0.14) | 0.37 (0.33, 0.41) | 0.56 (0.53, 0.60) | 0.28 (0.25, 0.30) |
| 96-102 | 0.51 (0.48, 0.53) | 0.21 (0.19, 0.23) | 0.14 (0.11, 0.16) | 0.34 (0.30, 0.38) | 0.61 (0.57, 0.65) | 0.26 (0.23, 0.29) |
| 90-96 | 0.53 (0.50, 0.56) | 0.22 (0.20, 0.24) | 0.13 (0.11, 0.15) | 0.40 (0.35, 0.44) | 0.62 (0.58, 0.65) | 0.27 (0.24, 0.30) |
| 84-90 | 0.59 (0.56, 0.62) | 0.23 (0.21, 0.25) | 0.13 (0.11, 0.15) | 0.42 (0.38, 0.47) | 0.68 (0.64, 0.73) | 0.29 (0.26, 0.31) |
| 78-84 | 0.65 (0.62, 0.68) | 0.26 (0.24, 0.29) | 0.14 (0.12, 0.17) | 0.47 (0.43, 0.52) | 0.76 (0.72, 0.81) | 0.34 (0.31, 0.37) |
| 72-78 | 0.70 (0.67, 0.73) | 0.24 (0.22, 0.26) | 0.13 (0.11, 0.15) | 0.54 (0.49, 0.59) | 0.80 (0.75, 0.84) | 0.30 (0.27, 0.33) |
| 66-72 | 0.75 (0.72, 0.78) | 0.24 (0.22, 0.26) | 0.17 (0.14, 0.19) | 0.62 (0.57, 0.67) | 0.83 (0.78, 0.88) | 0.28 (0.26, 0.31) |
| 60-66 | 0.81 (0.77, 0.84) | 0.27 (0.24, 0.29) | 0.16 (0.13, 0.18) | 0.59 (0.54, 0.64) | 0.94 (0.89, 0.99) | 0.33 (0.30, 0.36) |
| 54-60 | 0.87 (0.83, 0.90) | 0.31 (0.28, 0.33) | 0.18 (0.15, 0.21) | 0.68 (0.62, 0.73) | 0.98 (0.93, 1.03) | 0.38 (0.35, 0.41) |
| 48-54 | 0.98 (0.94, 1.02) | 0.28 (0.26, 0.30) | 0.18 (0.15, 0.21) | 0.78 (0.73, 0.84) | 1.10 (1.04, 1.15) | 0.34 (0.31, 0.37) |
| 42-48 | 1.06 (1.02, 1.11) | 0.29 (0.27, 0.31) | 0.16 (0.13, 0.18) | 0.89 (0.82, 0.95) | 1.17 (1.12, 1.23) | 0.37 (0.34, 0.40) |
| 36-42 | 1.24 (1.20, 1.29) | 0.29 (0.27, 0.32) | 0.17 (0.14, 0.20) | 1.08 (1.01, 1.15) | 1.34 (1.29, 1.40) | 0.37 (0.34, 0.40) |
| 30-36 | 1.40 (1.35, 1.45) | 0.31 (0.28, 0.33) | 0.18 (0.15, 0.20) | 1.23 (1.16, 1.30) | 1.50 (1.44, 1.56) | 0.39 (0.35, 0.42) |
| 24-30 | 1.66 (1.61, 1.71) | 0.31 (0.29, 0.34) | 0.19 (0.16, 0.22) | 1.41 (1.34, 1.49) | 1.81 (1.74, 1.88) | 0.39 (0.36, 0.42) |
| 18-24 | 2.11 (2.05, 2.17) | 0.34 (0.32, 0.37) | 0.21 (0.18, 0.24) | 1.83 (1.74, 1.92) | 2.28 (2.20, 2.36) | 0.43 (0.39, 0.46) |
| 12-18 | 3.02 (2.95, 3.09) | 0.38 (0.36, 0.41) | 0.22 (0.19, 0.25) | 2.66 (2.55, 2.76) | 3.25 (3.16, 3.34) | 0.48 (0.45, 0.52) |
| 6-12 | 5.53 (5.44, 5.62) | 0.48 (0.45, 0.50) | 0.33 (0.29, 0.37) | 5.18 (5.03, 5.32) | 5.74 (5.62, 5.86) | 0.56 (0.53, 0.60) |
| 0-6 | 7.16 (7.06, 7.26) | 0.52 (0.49, 0.55) | 0.34 (0.30, 0.38) | 6.94 (6.78, 7.11) | 7.29 (7.16, 7.42) | 0.63 (0.59, 0.67) |
| Referrals to pain clinic | | | | | | |
| 114-120 | 0.09 (0.07, 0.10) | 0.05 (0.04, 0.06) | 0.05 (0.03, 0.06) | 0.03 (0.02, 0.04) | 0.11 (0.09, 0.13) | 0.06 (0.05, 0.07) |
| 108-114 | 0.10 (0.08, 0.11) | 0.05 (0.04, 0.06) | 0.07 (0.05, 0.09) | 0.03 (0.02, 0.05) | 0.11 (0.09, 0.13) | 0.07 (0.05, 0.08) |
| 102-108 | 0.10 (0.09, 0.11) | 0.06 (0.05, 0.07) | 0.07 (0.05, 0.08) | 0.04 (0.03, 0.05) | 0.12 (0.10, 0.14) | 0.07 (0.05, 0.08) |
| 96-102 | 0.10 (0.09, 0.11) | 0.06 (0.05, 0.07) | 0.06 (0.05, 0.08) | 0.04 (0.03, 0.06) | 0.12 (0.11, 0.14) | 0.07 (0.05, 0.08) |
| 90-96 | 0.11 (0.10, 0.13) | 0.06 (0.05, 0.07) | 0.08 (0.06, 0.10) | 0.03 (0.02, 0.04) | 0.14 (0.12, 0.15) | 0.07 (0.06, 0.09) |
| 84-90 | 0.12 (0.10, 0.13) | 0.05 (0.04, 0.06) | 0.08 (0.06, 0.10) | 0.03 (0.02, 0.05) | 0.14 (0.12, 0.16) | 0.06 (0.05, 0.07) |
| 78-84 | 0.13 (0.11, 0.14) | 0.06 (0.05, 0.07) | 0.09 (0.07, 0.11) | 0.04 (0.03, 0.05) | 0.15 (0.13, 0.17) | 0.07 (0.06, 0.09) |
| 72-78 | 0.14 (0.12, 0.15) | 0.08 (0.07, 0.09) | 0.10 (0.08, 0.12) | 0.06 (0.04, 0.08) | 0.15 (0.13, 0.17) | 0.09 (0.08, 0.11) |
| 66-72 | 0.15 (0.13, 0.16) | 0.07 (0.06, 0.08) | 0.10 (0.08, 0.12) | 0.04 (0.03, 0.05) | 0.17 (0.15, 0.20) | 0.08 (0.07, 0.10) |
| 60-66 | 0.16 (0.14, 0.17) | 0.07 (0.06, 0.08) | 0.10 (0.08, 0.12) | 0.05 (0.03, 0.06) | 0.19 (0.17, 0.21) | 0.09 (0.07, 0.10) |
| 54-60 | 0.17 (0.15, 0.18) | 0.07 (0.06, 0.08) | 0.11 (0.09, 0.13) | 0.04 (0.03, 0.05) | 0.20 (0.18, 0.22) | 0.09 (0.07, 0.10) |
| 48-54 | 0.19 (0.17, 0.20) | 0.08 (0.07, 0.09) | 0.13 (0.10, 0.15) | 0.06 (0.04, 0.07) | 0.22 (0.20, 0.24) | 0.09 (0.08, 0.11) |
| 42-48 | 0.18 (0.17, 0.20) | 0.08 (0.07, 0.09) | 0.10 (0.08, 0.12) | 0.04 (0.03, 0.06) | 0.23 (0.21, 0.25) | 0.10 (0.08, 0.11) |
| 36-42 | 0.22 (0.20, 0.24) | 0.11 (0.09, 0.12) | 0.14 (0.12, 0.17) | 0.07 (0.05, 0.08) | 0.27 (0.24, 0.29) | 0.13 (0.11, 0.15) |
| 30-36 | 0.24 (0.22, 0.25) | 0.08 (0.07, 0.09) | 0.15 (0.12, 0.17) | 0.05 (0.03, 0.06) | 0.29 (0.26, 0.32) | 0.11 (0.09, 0.12) |
| 24-30 | 0.25 (0.23, 0.27) | 0.09 (0.08, 0.10) | 0.17 (0.15, 0.20) | 0.07 (0.05, 0.09) | 0.29 (0.27, 0.32) | 0.10 (0.08, 0.11) |
| 18-24 | 0.26 (0.24, 0.28) | 0.08 (0.07, 0.09) | 0.17 (0.14, 0.20) | 0.05 (0.03, 0.06) | 0.32 (0.29, 0.35) | 0.10 (0.09, 0.12) |
| 12-18 | 0.28 (0.26, 0.30) | 0.11 (0.09, 0.12) | 0.18 (0.16, 0.21) | 0.07 (0.05, 0.09) | 0.34 (0.31, 0.37) | 0.13 (0.11, 0.15) |
| 6-12 | 0.36 (0.34, 0.39) | 0.11 (0.10, 0.13) | 0.24 (0.21, 0.27) | 0.06 (0.05, 0.08) | 0.44 (0.40, 0.47) | 0.14 (0.12, 0.16) |
| 0-6 | 0.36 (0.33, 0.38) | 0.10 (0.09, 0.11) | 0.25 (0.22, 0.28) | 0.06 (0.04, 0.07) | 0.42 (0.39, 0.46) | 0.12 (0.10, 0.14) |
| Sick leave | | | | | | |
| 114-120 | 2.42 (2.36, 2.49) | 1.96 (1.91, 2.02) | 2.28 (2.18, 2.38) | 1.81 (1.73, 1.90) | 2.51 (2.43, 2.59) | 2.05 (1.98, 2.12) |
| 108-114 | 2.43 (2.37, 2.50) | 1.94 (1.88, 1.99) | 2.32 (2.22, 2.41) | 1.89 (1.80, 1.98) | 2.51 (2.43, 2.58) | 1.97 (1.90, 2.04) |
| 102-108 | 2.51 (2.45, 2.57) | 1.94 (1.89, 2.00) | 2.35 (2.25, 2.45) | 1.82 (1.74, 1.91) | 2.60 (2.52, 2.68) | 2.02 (1.94, 2.09) |
| 96-102 | 2.37 (2.31, 2.43) | 1.86 (1.81, 1.92) | 2.26 (2.16, 2.36) | 1.79 (1.71, 1.88) | 2.44 (2.36, 2.52) | 1.91 (1.84, 1.98) |
| 90-96 | 2.43 (2.37, 2.49) | 1.90 (1.84, 1.95) | 2.33 (2.23, 2.42) | 1.80 (1.71, 1.88) | 2.49 (2.41, 2.57) | 1.96 (1.89, 2.03) |
| 84-90 | 2.43 (2.37, 2.49) | 1.85 (1.80, 1.91) | 2.27 (2.17, 2.37) | 1.78 (1.70, 1.87) | 2.53 (2.45, 2.61) | 1.89 (1.82, 1.96) |
| 78-84 | 2.40 (2.34, 2.46) | 1.85 (1.80, 1.90) | 2.28 (2.18, 2.37) | 1.68 (1.59, 1.76) | 2.48 (2.40, 2.56) | 1.96 (1.89, 2.03) |
| 72-78 | 2.37 (2.31, 2.43) | 1.79 (1.74, 1.84) | 2.17 (2.07, 2.26) | 1.68 (1.60, 1.76) | 2.49 (2.41, 2.57) | 1.86 (1.79, 1.93) |
| 66-72 | 2.45 (2.39, 2.52) | 1.89 (1.84, 1.95) | 2.31 (2.22, 2.41) | 1.78 (1.70, 1.87) | 2.54 (2.46, 2.62) | 1.96 (1.89, 2.03) |
| 60-66 | 2.39 (2.33, 2.45) | 1.79 (1.74, 1.84) | 2.31 (2.22, 2.41) | 1.70 (1.61, 1.78) | 2.44 (2.36, 2.52) | 1.85 (1.78, 1.92) |
| 54-60 | 2.45 (2.39, 2.51) | 1.85 (1.79, 1.90) | 2.27 (2.18, 2.37) | 1.72 (1.63, 1.80) | 2.56 (2.48, 2.64) | 1.92 (1.85, 1.99) |
| 48-54 | 2.46 (2.40, 2.52) | 1.72 (1.67, 1.77) | 2.30 (2.20, 2.39) | 1.59 (1.51, 1.67) | 2.56 (2.48, 2.64) | 1.80 (1.73, 1.87) |
| 42-48 | 2.41 (2.34, 2.47) | 1.74 (1.69, 1.79) | 2.25 (2.16, 2.35) | 1.53 (1.45, 1.61) | 2.50 (2.42, 2.58) | 1.87 (1.80, 1.94) |
| 36-42 | 2.40 (2.34, 2.46) | 1.63 (1.58, 1.68) | 2.26 (2.17, 2.36) | 1.47 (1.39, 1.55) | 2.48 (2.40, 2.56) | 1.72 (1.66, 1.79) |
| 30-36 | 2.41 (2.34, 2.47) | 1.71 (1.66, 1.76) | 2.33 (2.24, 2.43) | 1.53 (1.45, 1.61) | 2.45 (2.37, 2.53) | 1.82 (1.75, 1.89) |
| 24-30 | 2.42 (2.36, 2.48) | 1.54 (1.50, 1.59) | 2.34 (2.24, 2.44) | 1.41 (1.33, 1.48) | 2.47 (2.39, 2.55) | 1.63 (1.56, 1.69) |
| 18-24 | 2.48 (2.42, 2.55) | 1.65 (1.60, 1.70) | 2.41 (2.31, 2.51) | 1.53 (1.45, 1.61) | 2.53 (2.45, 2.61) | 1.72 (1.65, 1.78) |
| 12-18 | 2.59 (2.52, 2.65) | 1.50 (1.45, 1.55) | 2.56 (2.46, 2.66) | 1.38 (1.31, 1.46) | 2.60 (2.52, 2.68) | 1.57 (1.51, 1.63) |
| 6-12 | 2.86 (2.80, 2.93) | 1.52 (1.47, 1.57) | 2.91 (2.80, 3.02) | 1.42 (1.34, 1.49) | 2.84 (2.75, 2.92) | 1.58 (1.52, 1.65) |
| 0-6 | 3.52 (3.44, 3.59) | 1.43 (1.38, 1.47) | 3.83 (3.70, 3.95) | 1.31 (1.23, 1.38) | 3.33 (3.24, 3.42) | 1.50 (1.44, 1.56) |

**Supplementary Table 2**

Prevalence rate ratio and 95% CIs of pharmacological and non-pharmacological care strategies in each 6-month interval with most recent 6-month interval adjusted for sex, age, geographical region, ethnicity, neighbourhood deprivation, comorbidity, BMI, smoking status, drinking status, and hip injury.

| Months prior to index date | Oral NSAIDs | | | | | |
| --- | --- | --- | --- | --- | --- | --- |
|  | Overall | | Male | | Female | |
|  | Cases; n=240,299 | Controls; n=240,299 | Cases; n=90,773 | Controls; n=90,773 | Cases; n=149,526 | Controls; n=149,526 |
| 114-120 | 0.73 (0.72, 0.74) | 1.73 (1.68, 1.77) | 0.64 (0.63, 0.66) | 1.71 (1.63, 1.79) | 0.77 (0.76, 0.79) | 1.73 (1.68, 1.79) |
| 108-114 | 0.75 (0.74, 0.76) | 1.71 (1.67, 1.75) | 0.65 (0.64, 0.67) | 1.68 (1.61, 1.76) | 0.81 (0.79, 0.82) | 1.72 (1.67, 1.77) |
| 102-108 | 0.76 (0.75, 0.77) | 1.71 (1.67, 1.76) | 0.67 (0.65, 0.69) | 1.66 (1.58, 1.74) | 0.81 (0.80, 0.82) | 1.74 (1.69, 1.79) |
| 96-102 | 0.76 (0.75, 0.77) | 1.69 (1.65, 1.73) | 0.67 (0.66, 0.69) | 1.66 (1.59, 1.74) | 0.81 (0.79, 0.82) | 1.70 (1.65, 1.75) |
| 90-96 | 0.76 (0.75, 0.77) | 1.67 (1.63, 1.71) | 0.68 (0.67, 0.70) | 1.62 (1.54, 1.69) | 0.81 (0.79, 0.82) | 1.69 (1.64, 1.74) |
| 84-90 | 0.76 (0.75, 0.77) | 1.60 (1.56, 1.64) | 0.68 (0.66, 0.70) | 1.58 (1.51, 1.66) | 0.81 (0.79, 0.82) | 1.61 (1.56, 1.66) |
| 78-84 | 0.76 (0.74, 0.77) | 1.61 (1.57, 1.65) | 0.69 (0.67, 0.70) | 1.57 (1.50, 1.65) | 0.79 (0.78, 0.81) | 1.62 (1.57, 1.67) |
| 72-78 | 0.75 (0.74, 0.76) | 1.55 (1.51, 1.59) | 0.68 (0.67, 0.70) | 1.53 (1.46, 1.61) | 0.79 (0.77, 0.80) | 1.56 (1.51, 1.60) |
| 66-72 | 0.75 (0.74, 0.76) | 1.53 (1.49, 1.57) | 0.68 (0.66, 0.70) | 1.49 (1.42, 1.56) | 0.79 (0.77, 0.80) | 1.55 (1.50, 1.60) |
| 60-66 | 0.75 (0.74, 0.76) | 1.48 (1.44, 1.52) | 0.67 (0.66, 0.69) | 1.46 (1.39, 1.53) | 0.79 (0.77, 0.80) | 1.49 (1.44, 1.54) |
| 54-60 | 0.74 (0.73, 0.76) | 1.45 (1.42, 1.49) | 0.68 (0.66, 0.70) | 1.41 (1.35, 1.48) | 0.78 (0.76, 0.79) | 1.47 (1.43, 1.51) |
| 48-54 | 0.74 (0.72, 0.75) | 1.39 (1.35, 1.42) | 0.67 (0.66, 0.69) | 1.38 (1.31, 1.44) | 0.77 (0.76, 0.78) | 1.39 (1.35, 1.43) |
| 42-48 | 0.74 (0.73, 0.75) | 1.34 (1.31, 1.38) | 0.69 (0.67, 0.70) | 1.32 (1.26, 1.39) | 0.77 (0.76, 0.78) | 1.35 (1.31, 1.40) |
| 36-42 | 0.74 (0.73, 0.75) | 1.29 (1.26, 1.33) | 0.69 (0.68, 0.71) | 1.28 (1.22, 1.34) | 0.76 (0.75, 0.78) | 1.30 (1.26, 1.34) |
| 30-36 | 0.75 (0.74, 0.76) | 1.24 (1.21, 1.28) | 0.71 (0.69, 0.73) | 1.24 (1.18, 1.30) | 0.78 (0.76, 0.79) | 1.24 (1.21, 1.28) |
| 24-30 | 0.76 (0.75, 0.77) | 1.17 (1.14, 1.20) | 0.73 (0.71, 0.75) | 1.15 (1.10, 1.21) | 0.78 (0.77, 0.79) | 1.18 (1.14, 1.22) |
| 18-24 | 0.80 (0.79, 0.81) | 1.15 (1.12, 1.18) | 0.77 (0.75, 0.79) | 1.13 (1.07, 1.18) | 0.81 (0.80, 0.83) | 1.16 (1.13, 1.20) |
| 12-18 | 0.87 (0.85, 0.88) | 1.09 (1.06, 1.12) | 0.85 (0.83, 0.87) | 1.10 (1.05, 1.16) | 0.88 (0.86, 0.89) | 1.09 (1.06, 1.13) |
| 6-12 | 0.98 (0.97, 0.99) | 1.06 (1.03, 1.09) | 0.96 (0.94, 0.98) | 1.03 (0.98, 1.08) | 0.99 (0.97, 1.00) | 1.07 (1.03, 1.10) |
| 0-6 | 1.00 (1.00, 1.00) | 1.00 (1.00, 1.00) | 1.00 (1.00, 1.00) | 1.00 (1.00, 1.00) | 1.00 (1.00, 1.00) | 1.00 (1.00, 1.00) |
| Topical NSAIDs | | | | | | |
| 114-120 | 0.29 (0.28, 0.30) | 0.51 (0.49, 0.53) | 0.25 (0.23, 0.26) | 0.45 (0.41, 0.48) | 0.31 (0.29, 0.32) | 0.53 (0.51, 0.56) |
| 108-114 | 0.32 (0.31, 0.33) | 0.53 (0.51, 0.55) | 0.27 (0.25, 0.28) | 0.49 (0.45, 0.53) | 0.34 (0.33, 0.35) | 0.55 (0.52, 0.57) |
| 102-108 | 0.34 (0.33, 0.35) | 0.57 (0.54, 0.59) | 0.29 (0.28, 0.31) | 0.49 (0.45, 0.53) | 0.36 (0.35, 0.38) | 0.60 (0.57, 0.62) |
| 96-102 | 0.36 (0.35, 0.37) | 0.60 (0.58, 0.63) | 0.30 (0.29, 0.32) | 0.55 (0.51, 0.59) | 0.39 (0.38, 0.40) | 0.63 (0.60, 0.65) |
| 90-96 | 0.38 (0.37, 0.39) | 0.63 (0.61, 0.66) | 0.33 (0.32, 0.35) | 0.58 (0.54, 0.62) | 0.40 (0.38, 0.41) | 0.66 (0.63, 0.68) |
| 84-90 | 0.41 (0.40, 0.42) | 0.65 (0.63, 0.68) | 0.36 (0.34, 0.38) | 0.62 (0.58, 0.67) | 0.43 (0.42, 0.44) | 0.66 (0.63, 0.69) |
| 78-84 | 0.43 (0.42, 0.45) | 0.68 (0.66, 0.71) | 0.39 (0.37, 0.41) | 0.66 (0.62, 0.71) | 0.46 (0.44, 0.47) | 0.69 (0.66, 0.72) |
| 72-78 | 0.46 (0.45, 0.47) | 0.71 (0.69, 0.74) | 0.42 (0.40, 0.44) | 0.68 (0.64, 0.73) | 0.48 (0.47, 0.50) | 0.72 (0.69, 0.76) |
| 66-72 | 0.49 (0.48, 0.51) | 0.75 (0.73, 0.78) | 0.45 (0.43, 0.47) | 0.70 (0.66, 0.76) | 0.51 (0.50, 0.53) | 0.77 (0.74, 0.80) |
| 60-66 | 0.54 (0.53, 0.55) | 0.80 (0.77, 0.83) | 0.49 (0.47, 0.51) | 0.77 (0.72, 0.82) | 0.56 (0.55, 0.58) | 0.81 (0.78, 0.85) |
| 54-60 | 0.56 (0.55, 0.58) | 0.83 (0.80, 0.86) | 0.50 (0.48, 0.53) | 0.79 (0.74, 0.85) | 0.59 (0.57, 0.61) | 0.85 (0.81, 0.88) |
| 48-54 | 0.59 (0.58, 0.60) | 0.83 (0.80, 0.86) | 0.54 (0.51, 0.56) | 0.84 (0.78, 0.90) | 0.61 (0.60, 0.63) | 0.83 (0.80, 0.86) |
| 42-48 | 0.63 (0.61, 0.64) | 0.87 (0.84, 0.90) | 0.59 (0.56, 0.61) | 0.84 (0.79, 0.90) | 0.65 (0.63, 0.67) | 0.88 (0.84, 0.92) |
| 36-42 | 0.68 (0.66, 0.70) | 0.88 (0.85, 0.91) | 0.65 (0.63, 0.68) | 0.86 (0.80, 0.91) | 0.69 (0.67, 0.71) | 0.89 (0.86, 0.93) |
| 30-36 | 0.71 (0.69, 0.72) | 0.92 (0.89, 0.95) | 0.67 (0.64, 0.70) | 0.88 (0.82, 0.94) | 0.72 (0.70, 0.74) | 0.94 (0.90, 0.97) |
| 24-30 | 0.76 (0.75, 0.78) | 0.95 (0.92, 0.98) | 0.74 (0.71, 0.77) | 0.94 (0.88, 1.01) | 0.77 (0.75, 0.79) | 0.95 (0.91, 0.99) |
| 18-24 | 0.83 (0.81, 0.85) | 0.98 (0.95, 1.02) | 0.79 (0.76, 0.82) | 0.98 (0.92, 1.04) | 0.85 (0.82, 0.87) | 0.99 (0.95, 1.03) |
| 12-18 | 0.91 (0.89, 0.93) | 0.99 (0.96, 1.02) | 0.89 (0.85, 0.92) | 0.96 (0.90, 1.02) | 0.92 (0.90, 0.95) | 1.00 (0.96, 1.04) |
| 6-12 | 1.02 (1.00, 1.04) | 1.01 (0.98, 1.04) | 1.02 (0.98, 1.06) | 0.99 (0.93, 1.06) | 1.02 (1.00, 1.05) | 1.02 (0.98, 1.05) |
| 0-6 | 1.00 (1.00, 1.00) | 1.00 (1.00, 1.00) | 1.00 (1.00, 1.00) | 1.00 (1.00, 1.00) | 1.00 (1.00, 1.00) | 1.00 (1.00, 1.00) |
| Paracetamol | | | | | | |
| 114-120 | 0.28 (0.27, 0.28) | 0.62 (0.61, 0.64) | 0.23 (0.22, 0.24) | 0.60 (0.57, 0.63) | 0.30 (0.29, 0.30) | 0.63 (0.61, 0.65) |
| 108-114 | 0.30 (0.30, 0.31) | 0.67 (0.65, 0.69) | 0.25 (0.24, 0.26) | 0.66 (0.62, 0.69) | 0.33 (0.32, 0.33) | 0.67 (0.65, 0.70) |
| 102-108 | 0.33 (0.32, 0.34) | 0.71 (0.69, 0.73) | 0.27 (0.26, 0.28) | 0.70 (0.66, 0.73) | 0.36 (0.35, 0.36) | 0.71 (0.69, 0.73) |
| 96-102 | 0.35 (0.34, 0.36) | 0.74 (0.72, 0.76) | 0.30 (0.29, 0.31) | 0.74 (0.71, 0.78) | 0.38 (0.37, 0.38) | 0.74 (0.72, 0.77) |
| 90-96 | 0.37 (0.37, 0.38) | 0.78 (0.76, 0.80) | 0.32 (0.30, 0.33) | 0.78 (0.74, 0.82) | 0.40 (0.39, 0.41) | 0.78 (0.76, 0.81) |
| 84-90 | 0.40 (0.39, 0.41) | 0.81 (0.79, 0.83) | 0.34 (0.33, 0.35) | 0.81 (0.77, 0.85) | 0.42 (0.42, 0.43) | 0.81 (0.78, 0.83) |
| 78-84 | 0.42 (0.41, 0.43) | 0.85 (0.83, 0.87) | 0.36 (0.35, 0.37) | 0.85 (0.81, 0.89) | 0.45 (0.44, 0.46) | 0.85 (0.82, 0.87) |
| 72-78 | 0.45 (0.44, 0.46) | 0.87 (0.85, 0.89) | 0.39 (0.38, 0.41) | 0.87 (0.83, 0.91) | 0.47 (0.46, 0.48) | 0.87 (0.85, 0.90) |
| 66-72 | 0.47 (0.46, 0.48) | 0.90 (0.88, 0.93) | 0.41 (0.40, 0.43) | 0.90 (0.85, 0.94) | 0.50 (0.49, 0.51) | 0.91 (0.88, 0.93) |
| 60-66 | 0.50 (0.49, 0.51) | 0.92 (0.90, 0.94) | 0.44 (0.42, 0.45) | 0.92 (0.87, 0.96) | 0.53 (0.52, 0.54) | 0.92 (0.89, 0.95) |
| 54-60 | 0.52 (0.51, 0.53) | 0.95 (0.92, 0.97) | 0.46 (0.45, 0.48) | 0.95 (0.90, 0.99) | 0.55 (0.54, 0.56) | 0.95 (0.92, 0.98) |
| 48-54 | 0.55 (0.54, 0.56) | 0.96 (0.93, 0.98) | 0.48 (0.47, 0.50) | 0.97 (0.92, 1.01) | 0.57 (0.56, 0.58) | 0.95 (0.93, 0.98) |
| 42-48 | 0.57 (0.57, 0.58) | 0.97 (0.95, 1.00) | 0.51 (0.50, 0.53) | 0.97 (0.92, 1.02) | 0.60 (0.59, 0.61) | 0.97 (0.95, 1.00) |
| 36-42 | 0.61 (0.60, 0.62) | 0.99 (0.96, 1.01) | 0.55 (0.53, 0.56) | 0.99 (0.94, 1.03) | 0.63 (0.62, 0.64) | 0.99 (0.96, 1.02) |
| 30-36 | 0.64 (0.63, 0.65) | 1.01 (0.98, 1.03) | 0.58 (0.57, 0.60) | 1.03 (0.98, 1.08) | 0.66 (0.65, 0.67) | 1.00 (0.97, 1.03) |
| 24-30 | 0.67 (0.66, 0.69) | 1.00 (0.98, 1.03) | 0.62 (0.60, 0.64) | 1.01 (0.97, 1.06) | 0.70 (0.69, 0.71) | 1.00 (0.97, 1.03) |
| 18-24 | 0.73 (0.71, 0.74) | 1.02 (0.99, 1.04) | 0.68 (0.66, 0.69) | 1.03 (0.99, 1.08) | 0.75 (0.73, 0.76) | 1.01 (0.99, 1.04) |
| 12-18 | 0.78 (0.77, 0.80) | 1.01 (0.98, 1.03) | 0.74 (0.72, 0.76) | 1.01 (0.96, 1.06) | 0.80 (0.79, 0.82) | 1.01 (0.98, 1.03) |
| 6-12 | 0.90 (0.89, 0.91) | 1.01 (0.98, 1.03) | 0.87 (0.85, 0.89) | 1.01 (0.96, 1.06) | 0.91 (0.89, 0.92) | 1.01 (0.98, 1.04) |
| 0-6 | 1.00 (1.00, 1.00) | 1.00 (1.00, 1.00) | 1.00 (1.00, 1.00) | 1.00 (1.00, 1.00) | 1.00 (1.00, 1.00) | 1.00 (1.00, 1.00) |
| Weak opioids | | | | | | |
| 114-120 | 0.43 (0.42, 0.44) | 1.05 (1.02, 1.09) | 0.34 (0.33, 0.36) | 1.05 (0.98, 1.12) | 0.47 (0.46, 0.49) | 1.05 (1.01, 1.09) |
| 108-114 | 0.44 (0.43, 0.45) | 1.05 (1.02, 1.09) | 0.34 (0.33, 0.36) | 1.08 (1.01, 1.15) | 0.48 (0.47, 0.50) | 1.04 (1.00, 1.08) |
| 102-108 | 0.44 (0.43, 0.45) | 1.05 (1.02, 1.09) | 0.34 (0.33, 0.36) | 1.06 (0.99, 1.13) | 0.49 (0.48, 0.51) | 1.05 (1.01, 1.09) |
| 96-102 | 0.44 (0.43, 0.45) | 1.03 (1.00, 1.07) | 0.35 (0.34, 0.36) | 1.05 (0.98, 1.12) | 0.48 (0.47, 0.50) | 1.02 (0.99, 1.06) |
| 90-96 | 0.44 (0.43, 0.45) | 0.99 (0.96, 1.03) | 0.35 (0.34, 0.37) | 0.98 (0.92, 1.05) | 0.48 (0.47, 0.49) | 1.00 (0.96, 1.04) |
| 84-90 | 0.44 (0.43, 0.45) | 0.99 (0.96, 1.03) | 0.35 (0.33, 0.36) | 1.02 (0.95, 1.09) | 0.49 (0.48, 0.50) | 0.99 (0.95, 1.03) |
| 78-84 | 0.44 (0.43, 0.45) | 0.99 (0.96, 1.03) | 0.35 (0.34, 0.37) | 1.02 (0.95, 1.09) | 0.48 (0.47, 0.49) | 0.99 (0.95, 1.02) |
| 72-78 | 0.44 (0.43, 0.45) | 0.97 (0.94, 1.01) | 0.36 (0.34, 0.37) | 0.95 (0.89, 1.02) | 0.47 (0.46, 0.49) | 0.98 (0.94, 1.02) |
| 66-72 | 0.44 (0.43, 0.45) | 0.98 (0.94, 1.01) | 0.36 (0.34, 0.37) | 0.98 (0.92, 1.05) | 0.48 (0.47, 0.50) | 0.97 (0.94, 1.01) |
| 60-66 | 0.44 (0.43, 0.45) | 0.95 (0.92, 0.98) | 0.38 (0.36, 0.39) | 0.95 (0.89, 1.02) | 0.48 (0.47, 0.49) | 0.95 (0.91, 0.99) |
| 54-60 | 0.45 (0.44, 0.46) | 0.95 (0.92, 0.98) | 0.37 (0.36, 0.39) | 0.97 (0.91, 1.03) | 0.48 (0.47, 0.50) | 0.95 (0.91, 0.98) |
| 48-54 | 0.45 (0.44, 0.46) | 0.93 (0.90, 0.97) | 0.39 (0.37, 0.40) | 0.94 (0.88, 1.01) | 0.49 (0.47, 0.50) | 0.93 (0.89, 0.97) |
| 42-48 | 0.47 (0.46, 0.48) | 0.95 (0.92, 0.98) | 0.40 (0.39, 0.42) | 0.97 (0.91, 1.04) | 0.49 (0.48, 0.51) | 0.95 (0.91, 0.98) |
| 36-42 | 0.48 (0.47, 0.49) | 0.93 (0.90, 0.97) | 0.43 (0.41, 0.44) | 0.94 (0.88, 1.01) | 0.50 (0.49, 0.52) | 0.93 (0.90, 0.97) |
| 30-36 | 0.50 (0.49, 0.51) | 0.95 (0.92, 0.98) | 0.44 (0.42, 0.45) | 0.98 (0.91, 1.04) | 0.52 (0.51, 0.54) | 0.94 (0.90, 0.98) |
| 24-30 | 0.53 (0.52, 0.54) | 0.94 (0.91, 0.97) | 0.47 (0.45, 0.49) | 0.95 (0.89, 1.01) | 0.56 (0.55, 0.57) | 0.93 (0.90, 0.97) |
| 18-24 | 0.57 (0.56, 0.58) | 0.96 (0.93, 0.99) | 0.51 (0.49, 0.53) | 0.96 (0.90, 1.03) | 0.60 (0.59, 0.62) | 0.96 (0.92, 0.99) |
| 12-18 | 0.66 (0.64, 0.67) | 0.96 (0.93, 0.99) | 0.59 (0.57, 0.61) | 0.97 (0.91, 1.03) | 0.68 (0.67, 0.70) | 0.95 (0.92, 0.99) |
| 6-12 | 0.83 (0.82, 0.85) | 0.98 (0.95, 1.02) | 0.80 (0.78, 0.83) | 0.99 (0.93, 1.06) | 0.84 (0.83, 0.86) | 0.98 (0.94, 1.02) |
| 0-6 | 1.00 (1.00, 1.00) | 1.00 (1.00, 1.00) | 1.00 (1.00, 1.00) | 1.00 (1.00, 1.00) | 1.00 (1.00, 1.00) | 1.00 (1.00, 1.00) |
| Strong opioids | | | | | | |
| 114-120 | 0.19 (0.19, 0.20) | 0.56 (0.54, 0.59) | 0.17 (0.16, 0.18) | 0.56 (0.51, 0.60) | 0.20 (0.20, 0.21) | 0.56 (0.54, 0.59) |
| 108-114 | 0.21 (0.21, 0.22) | 0.61 (0.58, 0.63) | 0.19 (0.18, 0.20) | 0.62 (0.57, 0.67) | 0.22 (0.22, 0.23) | 0.60 (0.57, 0.63) |
| 102-108 | 0.23 (0.22, 0.24) | 0.63 (0.61, 0.66) | 0.21 (0.20, 0.22) | 0.66 (0.61, 0.71) | 0.24 (0.23, 0.25) | 0.62 (0.59, 0.65) |
| 96-102 | 0.25 (0.24, 0.26) | 0.68 (0.65, 0.71) | 0.22 (0.21, 0.24) | 0.72 (0.67, 0.78) | 0.26 (0.25, 0.27) | 0.67 (0.64, 0.70) |
| 90-96 | 0.26 (0.26, 0.27) | 0.73 (0.70, 0.76) | 0.24 (0.23, 0.25) | 0.77 (0.72, 0.83) | 0.28 (0.27, 0.28) | 0.71 (0.68, 0.74) |
| 84-90 | 0.28 (0.27, 0.29) | 0.76 (0.73, 0.79) | 0.26 (0.25, 0.27) | 0.78 (0.72, 0.84) | 0.29 (0.28, 0.30) | 0.75 (0.72, 0.79) |
| 78-84 | 0.30 (0.29, 0.31) | 0.80 (0.76, 0.83) | 0.27 (0.26, 0.29) | 0.80 (0.74, 0.86) | 0.31 (0.30, 0.32) | 0.79 (0.76, 0.83) |
| 72-78 | 0.31 (0.31, 0.32) | 0.80 (0.77, 0.84) | 0.28 (0.27, 0.29) | 0.83 (0.77, 0.90) | 0.33 (0.32, 0.34) | 0.79 (0.76, 0.83) |
| 66-72 | 0.33 (0.32, 0.34) | 0.84 (0.81, 0.88) | 0.30 (0.29, 0.32) | 0.85 (0.79, 0.92) | 0.34 (0.33, 0.35) | 0.84 (0.80, 0.88) |
| 60-66 | 0.35 (0.34, 0.36) | 0.86 (0.83, 0.89) | 0.31 (0.30, 0.33) | 0.89 (0.83, 0.95) | 0.37 (0.36, 0.38) | 0.85 (0.81, 0.89) |
| 54-60 | 0.36 (0.35, 0.37) | 0.88 (0.85, 0.91) | 0.32 (0.31, 0.34) | 0.92 (0.85, 0.98) | 0.38 (0.37, 0.39) | 0.86 (0.83, 0.90) |
| 48-54 | 0.39 (0.38, 0.40) | 0.90 (0.87, 0.94) | 0.35 (0.33, 0.36) | 0.94 (0.87, 1.00) | 0.41 (0.40, 0.42) | 0.89 (0.85, 0.93) |
| 42-48 | 0.41 (0.40, 0.41) | 0.92 (0.89, 0.96) | 0.36 (0.35, 0.38) | 0.94 (0.88, 1.01) | 0.42 (0.41, 0.43) | 0.91 (0.87, 0.95) |
| 36-42 | 0.43 (0.42, 0.44) | 0.91 (0.88, 0.95) | 0.39 (0.37, 0.41) | 0.92 (0.86, 0.99) | 0.45 (0.44, 0.46) | 0.91 (0.87, 0.95) |
| 30-36 | 0.45 (0.44, 0.46) | 0.93 (0.90, 0.97) | 0.41 (0.39, 0.43) | 0.92 (0.86, 0.99) | 0.47 (0.45, 0.48) | 0.94 (0.90, 0.98) |
| 24-30 | 0.48 (0.47, 0.49) | 0.95 (0.92, 0.99) | 0.44 (0.42, 0.46) | 0.97 (0.91, 1.04) | 0.50 (0.49, 0.51) | 0.94 (0.90, 0.98) |
| 18-24 | 0.53 (0.52, 0.54) | 0.97 (0.93, 1.00) | 0.49 (0.47, 0.51) | 0.97 (0.91, 1.04) | 0.55 (0.53, 0.56) | 0.97 (0.92, 1.01) |
| 12-18 | 0.60 (0.59, 0.62) | 0.96 (0.92, 0.99) | 0.57 (0.55, 0.59) | 0.97 (0.90, 1.04) | 0.62 (0.60, 0.63) | 0.95 (0.91, 1.00) |
| 6-12 | 0.76 (0.74, 0.77) | 0.97 (0.94, 1.01) | 0.73 (0.71, 0.76) | 0.99 (0.92, 1.06) | 0.77 (0.75, 0.78) | 0.97 (0.93, 1.01) |
| 0-6 | 1.00 (1.00, 1.00) | 1.00 (1.00, 1.00) | 1.00 (1.00, 1.00) | 1.00 (1.00, 1.00) | 1.00 (1.00, 1.00) | 1.00 (1.00, 1.00) |
| Antidepressants | | | | | | |
| 114-120 | 0.63 (0.62, 0.64) | 0.84 (0.83, 0.86) | 0.54 (0.52, 0.56) | 0.82 (0.79, 0.86) | 0.66 (0.65, 0.67) | 0.85 (0.83, 0.87) |
| 108-114 | 0.66 (0.65, 0.67) | 0.88 (0.86, 0.89) | 0.57 (0.55, 0.59) | 0.85 (0.81, 0.89) | 0.69 (0.67, 0.70) | 0.88 (0.86, 0.90) |
| 102-108 | 0.67 (0.66, 0.69) | 0.89 (0.88, 0.91) | 0.58 (0.56, 0.60) | 0.89 (0.85, 0.93) | 0.70 (0.69, 0.72) | 0.90 (0.88, 0.92) |
| 96-102 | 0.69 (0.68, 0.70) | 0.91 (0.89, 0.93) | 0.60 (0.58, 0.62) | 0.89 (0.85, 0.93) | 0.72 (0.71, 0.73) | 0.91 (0.89, 0.94) |
| 90-96 | 0.71 (0.70, 0.72) | 0.93 (0.91, 0.94) | 0.62 (0.60, 0.64) | 0.92 (0.88, 0.97) | 0.74 (0.72, 0.75) | 0.93 (0.91, 0.95) |
| 84-90 | 0.72 (0.71, 0.74) | 0.94 (0.92, 0.96) | 0.64 (0.62, 0.67) | 0.93 (0.89, 0.97) | 0.75 (0.73, 0.76) | 0.94 (0.92, 0.96) |
| 78-84 | 0.74 (0.73, 0.75) | 0.96 (0.94, 0.98) | 0.66 (0.64, 0.68) | 0.96 (0.92, 1.00) | 0.76 (0.75, 0.78) | 0.96 (0.93, 0.98) |
| 72-78 | 0.75 (0.74, 0.77) | 0.96 (0.94, 0.98) | 0.67 (0.65, 0.69) | 0.96 (0.92, 1.00) | 0.78 (0.76, 0.79) | 0.96 (0.94, 0.99) |
| 66-72 | 0.77 (0.75, 0.78) | 0.97 (0.95, 0.99) | 0.69 (0.67, 0.72) | 0.97 (0.93, 1.02) | 0.79 (0.77, 0.80) | 0.97 (0.95, 0.99) |
| 60-66 | 0.78 (0.77, 0.79) | 0.99 (0.97, 1.01) | 0.71 (0.69, 0.74) | 1.00 (0.96, 1.04) | 0.80 (0.79, 0.82) | 0.98 (0.96, 1.00) |
| 54-60 | 0.80 (0.78, 0.81) | 0.98 (0.96, 1.00) | 0.73 (0.71, 0.76) | 1.00 (0.96, 1.04) | 0.82 (0.80, 0.83) | 0.98 (0.96, 1.00) |
| 48-54 | 0.81 (0.80, 0.83) | 0.99 (0.97, 1.01) | 0.75 (0.72, 0.77) | 1.01 (0.97, 1.06) | 0.83 (0.82, 0.85) | 0.99 (0.97, 1.01) |
| 42-48 | 0.83 (0.81, 0.84) | 1.01 (0.99, 1.03) | 0.77 (0.74, 0.79) | 1.02 (0.98, 1.07) | 0.84 (0.83, 0.86) | 1.00 (0.98, 1.02) |
| 36-42 | 0.84 (0.83, 0.85) | 1.01 (0.99, 1.03) | 0.78 (0.75, 0.81) | 1.01 (0.97, 1.06) | 0.86 (0.84, 0.87) | 1.00 (0.98, 1.03) |
| 30-36 | 0.85 (0.84, 0.87) | 1.01 (0.99, 1.03) | 0.79 (0.76, 0.82) | 1.01 (0.97, 1.06) | 0.87 (0.86, 0.89) | 1.01 (0.99, 1.03) |
| 24-30 | 0.88 (0.87, 0.89) | 1.02 (1.00, 1.04) | 0.83 (0.80, 0.86) | 1.01 (0.97, 1.06) | 0.90 (0.88, 0.91) | 1.02 (0.99, 1.04) |
| 18-24 | 0.90 (0.89, 0.92) | 1.01 (0.99, 1.03) | 0.85 (0.82, 0.87) | 1.01 (0.97, 1.06) | 0.92 (0.90, 0.93) | 1.01 (0.99, 1.04) |
| 12-18 | 0.93 (0.92, 0.95) | 1.00 (0.98, 1.02) | 0.89 (0.86, 0.92) | 1.00 (0.96, 1.05) | 0.95 (0.93, 0.96) | 1.00 (0.98, 1.03) |
| 6-12 | 0.98 (0.96, 0.99) | 1.00 (0.98, 1.02) | 0.96 (0.93, 0.99) | 1.01 (0.97, 1.05) | 0.98 (0.97, 1.00) | 1.00 (0.98, 1.02) |
| 0-6 | 1.00 (1.00, 1.00) | 1.00 (1.00, 1.00) | 1.00 (1.00, 1.00) | 1.00 (1.00, 1.00) | 1.00 (1.00, 1.00) | 1.00 (1.00, 1.00) |
| Antiepileptics | | | | | | |
| 114-120 | 0.29 (0.28, 0.31) | 0.52 (0.50, 0.54) | 0.31 (0.29, 0.33) | 0.57 (0.52, 0.61) | 0.29 (0.28, 0.30) | 0.50 (0.47, 0.53) |
| 108-114 | 0.31 (0.30, 0.32) | 0.55 (0.53, 0.58) | 0.32 (0.30, 0.35) | 0.60 (0.56, 0.65) | 0.31 (0.29, 0.32) | 0.53 (0.50, 0.56) |
| 102-108 | 0.33 (0.32, 0.34) | 0.58 (0.56, 0.61) | 0.34 (0.32, 0.37) | 0.62 (0.58, 0.67) | 0.33 (0.31, 0.34) | 0.56 (0.53, 0.59) |
| 96-102 | 0.35 (0.34, 0.36) | 0.62 (0.59, 0.65) | 0.36 (0.33, 0.38) | 0.67 (0.62, 0.72) | 0.35 (0.33, 0.36) | 0.60 (0.56, 0.63) |
| 90-96 | 0.37 (0.35, 0.38) | 0.63 (0.60, 0.66) | 0.37 (0.35, 0.40) | 0.69 (0.64, 0.74) | 0.36 (0.35, 0.38) | 0.60 (0.57, 0.63) |
| 84-90 | 0.39 (0.37, 0.40) | 0.66 (0.63, 0.69) | 0.39 (0.37, 0.42) | 0.72 (0.67, 0.77) | 0.38 (0.37, 0.40) | 0.64 (0.60, 0.67) |
| 78-84 | 0.42 (0.40, 0.43) | 0.69 (0.66, 0.72) | 0.42 (0.40, 0.45) | 0.73 (0.68, 0.78) | 0.41 (0.40, 0.43) | 0.67 (0.64, 0.71) |
| 72-78 | 0.44 (0.42, 0.45) | 0.73 (0.70, 0.76) | 0.44 (0.42, 0.47) | 0.78 (0.72, 0.83) | 0.44 (0.42, 0.46) | 0.70 (0.67, 0.74) |
| 66-72 | 0.46 (0.45, 0.48) | 0.74 (0.71, 0.78) | 0.47 (0.44, 0.50) | 0.78 (0.73, 0.84) | 0.46 (0.44, 0.48) | 0.73 (0.69, 0.76) |
| 60-66 | 0.49 (0.48, 0.51) | 0.77 (0.74, 0.80) | 0.51 (0.48, 0.53) | 0.80 (0.74, 0.85) | 0.49 (0.47, 0.51) | 0.76 (0.72, 0.79) |
| 54-60 | 0.52 (0.50, 0.54) | 0.79 (0.76, 0.83) | 0.53 (0.50, 0.56) | 0.81 (0.75, 0.87) | 0.52 (0.50, 0.54) | 0.79 (0.75, 0.82) |
| 48-54 | 0.56 (0.54, 0.57) | 0.82 (0.78, 0.85) | 0.56 (0.53, 0.59) | 0.85 (0.79, 0.91) | 0.55 (0.53, 0.57) | 0.80 (0.76, 0.84) |
| 42-48 | 0.59 (0.57, 0.61) | 0.84 (0.81, 0.88) | 0.59 (0.56, 0.62) | 0.88 (0.82, 0.94) | 0.59 (0.57, 0.61) | 0.82 (0.79, 0.86) |
| 36-42 | 0.62 (0.60, 0.64) | 0.87 (0.84, 0.90) | 0.61 (0.58, 0.65) | 0.91 (0.85, 0.97) | 0.62 (0.60, 0.65) | 0.85 (0.81, 0.89) |
| 30-36 | 0.66 (0.65, 0.68) | 0.88 (0.85, 0.92) | 0.65 (0.62, 0.69) | 0.89 (0.83, 0.96) | 0.67 (0.65, 0.69) | 0.88 (0.84, 0.92) |
| 24-30 | 0.71 (0.69, 0.73) | 0.92 (0.88, 0.95) | 0.70 (0.67, 0.74) | 0.94 (0.88, 1.01) | 0.71 (0.69, 0.74) | 0.91 (0.87, 0.95) |
| 18-24 | 0.76 (0.74, 0.78) | 0.93 (0.90, 0.97) | 0.74 (0.71, 0.78) | 0.94 (0.88, 1.01) | 0.77 (0.74, 0.79) | 0.93 (0.89, 0.97) |
| 12-18 | 0.83 (0.81, 0.85) | 0.96 (0.92, 0.99) | 0.81 (0.77, 0.85) | 0.97 (0.91, 1.04) | 0.84 (0.81, 0.86) | 0.95 (0.91, 0.99) |
| 6-12 | 0.93 (0.91, 0.96) | 1.00 (0.96, 1.03) | 0.92 (0.87, 0.96) | 0.99 (0.93, 1.06) | 0.94 (0.91, 0.97) | 1.00 (0.95, 1.04) |
| 0-6 | 1.00 (1.00, 1.00) | 1.00 (1.00, 1.00) | 1.00 (1.00, 1.00) | 1.00 (1.00, 1.00) | 1.00 (1.00, 1.00) | 1.00 (1.00, 1.00) |
| Bisphosphonates | | | | | | |
| 114-120 | 0.36 (0.35, 0.38) | 0.54 (0.51, 0.56) | 0.24 (0.20, 0.28) | 0.49 (0.42, 0.57) | 0.38 (0.36, 0.40) | 0.54 (0.52, 0.57) |
| 108-114 | 0.39 (0.38, 0.41) | 0.58 (0.56, 0.61) | 0.25 (0.22, 0.29) | 0.56 (0.48, 0.65) | 0.41 (0.40, 0.43) | 0.59 (0.56, 0.62) |
| 102-108 | 0.43 (0.41, 0.44) | 0.64 (0.61, 0.67) | 0.28 (0.25, 0.33) | 0.62 (0.53, 0.71) | 0.45 (0.43, 0.47) | 0.64 (0.61, 0.67) |
| 96-102 | 0.46 (0.44, 0.48) | 0.67 (0.64, 0.70) | 0.31 (0.27, 0.35) | 0.65 (0.57, 0.75) | 0.48 (0.46, 0.50) | 0.67 (0.64, 0.71) |
| 90-96 | 0.50 (0.48, 0.52) | 0.71 (0.68, 0.75) | 0.34 (0.30, 0.39) | 0.67 (0.58, 0.77) | 0.52 (0.50, 0.55) | 0.72 (0.69, 0.75) |
| 84-90 | 0.54 (0.52, 0.56) | 0.76 (0.73, 0.79) | 0.36 (0.32, 0.41) | 0.72 (0.63, 0.83) | 0.56 (0.54, 0.58) | 0.77 (0.73, 0.80) |
| 78-84 | 0.57 (0.55, 0.59) | 0.79 (0.76, 0.83) | 0.39 (0.35, 0.44) | 0.78 (0.68, 0.89) | 0.60 (0.58, 0.62) | 0.80 (0.76, 0.83) |
| 72-78 | 0.61 (0.58, 0.63) | 0.83 (0.79, 0.86) | 0.40 (0.36, 0.45) | 0.81 (0.71, 0.92) | 0.64 (0.61, 0.66) | 0.83 (0.79, 0.86) |
| 66-72 | 0.63 (0.61, 0.66) | 0.85 (0.82, 0.89) | 0.42 (0.38, 0.48) | 0.82 (0.71, 0.93) | 0.67 (0.64, 0.69) | 0.86 (0.82, 0.90) |
| 60-66 | 0.67 (0.65, 0.69) | 0.89 (0.85, 0.93) | 0.47 (0.42, 0.53) | 0.83 (0.73, 0.95) | 0.70 (0.67, 0.72) | 0.90 (0.86, 0.94) |
| 54-60 | 0.70 (0.68, 0.73) | 0.90 (0.87, 0.94) | 0.49 (0.44, 0.55) | 0.86 (0.75, 0.97) | 0.73 (0.70, 0.76) | 0.91 (0.87, 0.95) |
| 48-54 | 0.73 (0.70, 0.75) | 0.92 (0.88, 0.96) | 0.52 (0.47, 0.58) | 0.88 (0.78, 1.01) | 0.76 (0.73, 0.79) | 0.92 (0.89, 0.96) |
| 42-48 | 0.76 (0.74, 0.79) | 0.94 (0.90, 0.98) | 0.57 (0.52, 0.64) | 0.93 (0.82, 1.06) | 0.79 (0.76, 0.82) | 0.94 (0.90, 0.98) |
| 36-42 | 0.79 (0.76, 0.82) | 0.95 (0.91, 0.99) | 0.60 (0.54, 0.66) | 0.95 (0.84, 1.08) | 0.82 (0.79, 0.85) | 0.95 (0.91, 0.99) |
| 30-36 | 0.82 (0.79, 0.85) | 0.97 (0.93, 1.01) | 0.66 (0.59, 0.73) | 0.93 (0.82, 1.06) | 0.84 (0.82, 0.88) | 0.97 (0.93, 1.01) |
| 24-30 | 0.85 (0.82, 0.88) | 0.95 (0.92, 0.99) | 0.71 (0.65, 0.79) | 0.95 (0.83, 1.08) | 0.87 (0.84, 0.90) | 0.95 (0.92, 1.00) |
| 18-24 | 0.89 (0.86, 0.92) | 0.98 (0.94, 1.02) | 0.77 (0.70, 0.85) | 0.95 (0.84, 1.08) | 0.90 (0.87, 0.93) | 0.98 (0.94, 1.03) |
| 12-18 | 0.92 (0.89, 0.95) | 0.99 (0.95, 1.03) | 0.85 (0.78, 0.94) | 1.01 (0.89, 1.14) | 0.93 (0.90, 0.97) | 0.99 (0.95, 1.04) |
| 6-12 | 0.96 (0.93, 0.99) | 1.00 (0.96, 1.04) | 0.92 (0.84, 1.01) | 1.00 (0.88, 1.13) | 0.96 (0.93, 0.99) | 1.00 (0.96, 1.04) |
| 0-6 | 1.00 (1.00, 1.00) | 1.00 (1.00, 1.00) | 1.00 (1.00, 1.00) | 1.00 (1.00, 1.00) | 1.00 (1.00, 1.00) | 1.00 (1.00, 1.00) |
| All corticosteroids | | | | | | |
| 114-120 | 0.82 (0.81, 0.83) | 1.00 (0.99, 1.01) | 0.75 (0.73, 0.77) | 0.99 (0.96, 1.01) | 0.85 (0.84, 0.86) | 1.01 (0.99, 1.02) |
| 108-114 | 0.86 (0.85, 0.87) | 1.00 (0.99, 1.02) | 0.79 (0.77, 0.81) | 0.99 (0.97, 1.02) | 0.89 (0.88, 0.91) | 1.01 (0.99, 1.03) |
| 102-108 | 0.88 (0.87, 0.89) | 1.04 (1.02, 1.05) | 0.81 (0.79, 0.82) | 1.04 (1.01, 1.06) | 0.91 (0.90, 0.93) | 1.04 (1.02, 1.06) |
| 96-102 | 0.89 (0.88, 0.90) | 1.03 (1.02, 1.05) | 0.81 (0.79, 0.83) | 1.02 (1.00, 1.05) | 0.93 (0.91, 0.94) | 1.03 (1.02, 1.05) |
| 90-96 | 0.90 (0.89, 0.91) | 1.07 (1.05, 1.08) | 0.83 (0.81, 0.85) | 1.06 (1.03, 1.09) | 0.94 (0.93, 0.95) | 1.07 (1.05, 1.09) |
| 84-90 | 0.92 (0.91, 0.93) | 1.05 (1.04, 1.07) | 0.85 (0.84, 0.87) | 1.04 (1.02, 1.07) | 0.95 (0.94, 0.97) | 1.05 (1.04, 1.07) |
| 78-84 | 0.93 (0.92, 0.94) | 1.09 (1.07, 1.10) | 0.87 (0.86, 0.89) | 1.09 (1.06, 1.11) | 0.95 (0.94, 0.97) | 1.09 (1.07, 1.11) |
| 72-78 | 0.94 (0.93, 0.95) | 1.06 (1.05, 1.08) | 0.87 (0.85, 0.88) | 1.06 (1.03, 1.08) | 0.97 (0.96, 0.99) | 1.07 (1.05, 1.08) |
| 66-72 | 0.95 (0.94, 0.96) | 1.10 (1.09, 1.12) | 0.90 (0.88, 0.92) | 1.11 (1.08, 1.13) | 0.98 (0.97, 1.00) | 1.10 (1.08, 1.12) |
| 60-66 | 0.96 (0.95, 0.97) | 1.06 (1.04, 1.07) | 0.91 (0.89, 0.92) | 1.05 (1.02, 1.07) | 0.99 (0.97, 1.00) | 1.06 (1.05, 1.08) |
| 54-60 | 0.97 (0.96, 0.98) | 1.09 (1.08, 1.11) | 0.91 (0.90, 0.93) | 1.10 (1.07, 1.12) | 1.00 (0.99, 1.01) | 1.09 (1.07, 1.11) |
| 48-54 | 0.98 (0.97, 0.99) | 1.07 (1.05, 1.08) | 0.93 (0.92, 0.95) | 1.07 (1.04, 1.09) | 1.00 (0.99, 1.02) | 1.06 (1.05, 1.08) |
| 42-48 | 0.98 (0.97, 0.99) | 1.10 (1.08, 1.11) | 0.93 (0.92, 0.95) | 1.11 (1.08, 1.13) | 1.01 (0.99, 1.02) | 1.09 (1.07, 1.11) |
| 36-42 | 0.98 (0.97, 0.99) | 1.06 (1.04, 1.07) | 0.95 (0.93, 0.96) | 1.07 (1.04, 1.09) | 1.00 (0.99, 1.02) | 1.05 (1.04, 1.07) |
| 30-36 | 1.00 (0.99, 1.01) | 1.09 (1.07, 1.10) | 0.97 (0.95, 0.99) | 1.09 (1.06, 1.11) | 1.02 (1.01, 1.03) | 1.08 (1.07, 1.10) |
| 24-30 | 1.00 (0.99, 1.02) | 1.04 (1.03, 1.05) | 0.97 (0.95, 0.99) | 1.03 (1.01, 1.06) | 1.02 (1.01, 1.04) | 1.04 (1.03, 1.06) |
| 18-24 | 1.01 (1.00, 1.03) | 1.07 (1.05, 1.08) | 0.99 (0.97, 1.01) | 1.08 (1.05, 1.10) | 1.03 (1.01, 1.04) | 1.06 (1.05, 1.08) |
| 12-18 | 1.02 (1.01, 1.04) | 1.03 (1.02, 1.05) | 1.00 (0.98, 1.02) | 1.03 (1.00, 1.05) | 1.04 (1.02, 1.05) | 1.03 (1.01, 1.05) |
| 6-12 | 1.03 (1.02, 1.05) | 1.04 (1.03, 1.06) | 1.01 (0.99, 1.03) | 1.05 (1.03, 1.08) | 1.05 (1.03, 1.06) | 1.04 (1.02, 1.05) |
| 0-6 | 1.00 (1.00, 1.00) | 1.00 (1.00, 1.00) | 1.00 (1.00, 1.00) | 1.00 (1.00, 1.00) | 1.00 (1.00, 1.00) | 1.00 (1.00, 1.00) |
| Systemic corticosteroids | | | | | | |
| 114-120 | 0.53 (0.51, 0.55) | 0.74 (0.71, 0.77) | 0.49 (0.46, 0.52) | 0.74 (0.69, 0.79) | 0.55 (0.53, 0.57) | 0.74 (0.71, 0.77) |
| 108-114 | 0.57 (0.55, 0.59) | 0.74 (0.72, 0.77) | 0.51 (0.48, 0.54) | 0.74 (0.70, 0.80) | 0.60 (0.58, 0.62) | 0.75 (0.71, 0.78) |
| 102-108 | 0.59 (0.57, 0.61) | 0.78 (0.75, 0.81) | 0.53 (0.50, 0.56) | 0.79 (0.74, 0.84) | 0.61 (0.59, 0.64) | 0.78 (0.74, 0.81) |
| 96-102 | 0.61 (0.59, 0.63) | 0.79 (0.76, 0.82) | 0.53 (0.50, 0.56) | 0.79 (0.74, 0.85) | 0.64 (0.62, 0.67) | 0.79 (0.76, 0.82) |
| 90-96 | 0.64 (0.62, 0.66) | 0.84 (0.81, 0.87) | 0.56 (0.53, 0.60) | 0.85 (0.80, 0.91) | 0.67 (0.65, 0.70) | 0.83 (0.80, 0.87) |
| 84-90 | 0.64 (0.62, 0.66) | 0.83 (0.80, 0.86) | 0.56 (0.53, 0.60) | 0.85 (0.79, 0.90) | 0.68 (0.65, 0.70) | 0.82 (0.78, 0.85) |
| 78-84 | 0.68 (0.66, 0.70) | 0.91 (0.88, 0.94) | 0.62 (0.58, 0.65) | 0.91 (0.85, 0.96) | 0.71 (0.69, 0.74) | 0.91 (0.87, 0.95) |
| 72-78 | 0.69 (0.67, 0.71) | 0.87 (0.84, 0.90) | 0.62 (0.58, 0.65) | 0.89 (0.83, 0.94) | 0.73 (0.70, 0.76) | 0.87 (0.83, 0.90) |
| 66-72 | 0.73 (0.71, 0.75) | 0.94 (0.91, 0.98) | 0.66 (0.63, 0.70) | 0.93 (0.88, 1.00) | 0.77 (0.74, 0.79) | 0.95 (0.91, 0.99) |
| 60-66 | 0.75 (0.73, 0.78) | 0.89 (0.86, 0.92) | 0.67 (0.63, 0.70) | 0.87 (0.82, 0.93) | 0.79 (0.77, 0.82) | 0.90 (0.86, 0.94) |
| 54-60 | 0.79 (0.77, 0.81) | 0.97 (0.94, 1.00) | 0.70 (0.67, 0.74) | 0.98 (0.92, 1.04) | 0.83 (0.80, 0.86) | 0.97 (0.93, 1.01) |
| 48-54 | 0.81 (0.79, 0.84) | 0.95 (0.92, 0.99) | 0.75 (0.71, 0.79) | 0.95 (0.90, 1.02) | 0.84 (0.82, 0.87) | 0.95 (0.91, 0.99) |
| 42-48 | 0.85 (0.82, 0.87) | 1.01 (0.98, 1.04) | 0.80 (0.77, 0.85) | 1.04 (0.98, 1.10) | 0.87 (0.84, 0.90) | 1.00 (0.96, 1.04) |
| 36-42 | 0.88 (0.85, 0.90) | 0.97 (0.93, 1.00) | 0.83 (0.79, 0.87) | 1.01 (0.95, 1.08) | 0.90 (0.87, 0.93) | 0.95 (0.91, 0.99) |
| 30-36 | 0.94 (0.92, 0.97) | 1.02 (0.99, 1.06) | 0.89 (0.85, 0.94) | 1.04 (0.98, 1.11) | 0.97 (0.94, 1.00) | 1.02 (0.98, 1.06) |
| 24-30 | 0.99 (0.96, 1.02) | 0.99 (0.95, 1.02) | 0.92 (0.88, 0.97) | 0.98 (0.92, 1.04) | 1.02 (0.99, 1.05) | 0.99 (0.95, 1.03) |
| 18-24 | 1.06 (1.03, 1.09) | 1.06 (1.02, 1.09) | 1.01 (0.96, 1.06) | 1.07 (1.01, 1.14) | 1.08 (1.05, 1.12) | 1.05 (1.01, 1.09) |
| 12-18 | 1.14 (1.12, 1.17) | 1.02 (0.99, 1.06) | 1.08 (1.03, 1.14) | 1.01 (0.95, 1.08) | 1.17 (1.14, 1.21) | 1.02 (0.98, 1.07) |
| 6-12 | 1.23 (1.19, 1.26) | 1.06 (1.02, 1.09) | 1.20 (1.15, 1.26) | 1.08 (1.01, 1.14) | 1.24 (1.20, 1.28) | 1.05 (1.01, 1.09) |
| 0-6 | 1.00 (1.00, 1.00) | 1.00 (1.00, 1.00) | 1.00 (1.00, 1.00) | 1.00 (1.00, 1.00) | 1.00 (1.00, 1.00) | 1.00 (1.00, 1.00) |
| GP visits | | | | | | |
| 114-120 | 1.38 (1.33, 1.44) | 1.83 (1.74, 1.92) | 1.20 (1.12, 1.29) | 1.63 (1.50, 1.78) | 1.49 (1.41, 1.57) | 1.93 (1.82, 2.05) |
| 108-114 | 1.42 (1.36, 1.48) | 1.87 (1.78, 1.96) | 1.22 (1.14, 1.31) | 1.68 (1.55, 1.83) | 1.54 (1.46, 1.62) | 1.97 (1.86, 2.09) |
| 102-108 | 1.38 (1.33, 1.44) | 1.77 (1.68, 1.86) | 1.20 (1.12, 1.29) | 1.62 (1.49, 1.76) | 1.49 (1.42, 1.57) | 1.84 (1.74, 1.96) |
| 96-102 | 1.37 (1.31, 1.42) | 1.83 (1.74, 1.92) | 1.19 (1.11, 1.27) | 1.70 (1.56, 1.85) | 1.47 (1.40, 1.55) | 1.89 (1.79, 2.01) |
| 90-96 | 1.34 (1.29, 1.40) | 1.70 (1.61, 1.78) | 1.18 (1.11, 1.27) | 1.54 (1.42, 1.68) | 1.43 (1.36, 1.51) | 1.78 (1.67, 1.89) |
| 84-90 | 1.31 (1.25, 1.36) | 1.71 (1.63, 1.80) | 1.16 (1.08, 1.24) | 1.55 (1.42, 1.69) | 1.40 (1.33, 1.47) | 1.80 (1.69, 1.91) |
| 78-84 | 1.27 (1.22, 1.32) | 1.64 (1.56, 1.72) | 1.17 (1.10, 1.26) | 1.52 (1.40, 1.65) | 1.33 (1.26, 1.40) | 1.70 (1.60, 1.81) |
| 72-78 | 1.24 (1.19, 1.29) | 1.60 (1.52, 1.68) | 1.13 (1.06, 1.21) | 1.53 (1.41, 1.66) | 1.30 (1.23, 1.37) | 1.63 (1.54, 1.73) |
| 66-72 | 1.21 (1.16, 1.26) | 1.54 (1.46, 1.62) | 1.14 (1.07, 1.23) | 1.45 (1.33, 1.58) | 1.25 (1.18, 1.32) | 1.59 (1.49, 1.69) |
| 60-66 | 1.20 (1.15, 1.25) | 1.54 (1.47, 1.62) | 1.15 (1.07, 1.23) | 1.47 (1.35, 1.60) | 1.23 (1.17, 1.30) | 1.58 (1.49, 1.68) |
| 54-60 | 1.18 (1.13, 1.23) | 1.49 (1.42, 1.57) | 1.13 (1.06, 1.21) | 1.41 (1.30, 1.54) | 1.21 (1.14, 1.27) | 1.53 (1.44, 1.63) |
| 48-54 | 1.16 (1.12, 1.21) | 1.45 (1.38, 1.52) | 1.11 (1.03, 1.19) | 1.41 (1.29, 1.53) | 1.20 (1.13, 1.26) | 1.47 (1.38, 1.57) |
| 42-48 | 1.15 (1.11, 1.20) | 1.40 (1.33, 1.47) | 1.13 (1.05, 1.21) | 1.38 (1.26, 1.50) | 1.17 (1.11, 1.23) | 1.41 (1.33, 1.50) |
| 36-42 | 1.11 (1.06, 1.16) | 1.37 (1.30, 1.44) | 1.08 (1.01, 1.16) | 1.34 (1.23, 1.46) | 1.13 (1.07, 1.19) | 1.39 (1.31, 1.48) |
| 30-36 | 1.07 (1.03, 1.12) | 1.28 (1.22, 1.35) | 1.02 (0.95, 1.09) | 1.27 (1.17, 1.39) | 1.10 (1.05, 1.17) | 1.29 (1.21, 1.37) |
| 24-30 | 1.07 (1.02, 1.12) | 1.26 (1.19, 1.32) | 1.03 (0.96, 1.11) | 1.23 (1.13, 1.34) | 1.09 (1.03, 1.15) | 1.27 (1.19, 1.35) |
| 18-24 | 1.05 (1.00, 1.09) | 1.21 (1.15, 1.27) | 1.02 (0.95, 1.09) | 1.21 (1.11, 1.32) | 1.06 (1.00, 1.12) | 1.21 (1.13, 1.29) |
| 12-18 | 1.02 (0.97, 1.06) | 1.14 (1.08, 1.20) | 1.01 (0.94, 1.08) | 1.13 (1.04, 1.24) | 1.02 (0.97, 1.08) | 1.14 (1.07, 1.22) |
| 6-12 | 1.00 (0.96, 1.05) | 1.06 (1.01, 1.12) | 1.00 (0.93, 1.07) | 1.06 (0.97, 1.16) | 1.01 (0.95, 1.07) | 1.06 (1.00, 1.14) |
| 0-6 | 1.00 (1.00, 1.00) | 1.00 (1.00, 1.00) | 1.00 (1.00, 1.00) | 1.00 (1.00, 1.00) | 1.00 (1.00, 1.00) | 1.00 (1.00, 1.00) |
| Referrals for physiotherapy | | | | | | |
| 114-120 | 0.43 (0.41, 0.45) | 0.76 (0.72, 0.81) | 0.39 (0.36, 0.42) | 0.75 (0.67, 0.85) | 0.45 (0.43, 0.48) | 0.77 (0.71, 0.83) |
| 108-114 | 0.43 (0.42, 0.45) | 0.76 (0.72, 0.81) | 0.38 (0.35, 0.42) | 0.75 (0.66, 0.84) | 0.46 (0.44, 0.49) | 0.77 (0.71, 0.83) |
| 102-108 | 0.46 (0.44, 0.48) | 0.82 (0.77, 0.87) | 0.39 (0.36, 0.43) | 0.77 (0.68, 0.87) | 0.49 (0.47, 0.52) | 0.84 (0.78, 0.90) |
| 96-102 | 0.45 (0.43, 0.47) | 0.81 (0.76, 0.87) | 0.37 (0.34, 0.40) | 0.77 (0.68, 0.87) | 0.49 (0.47, 0.52) | 0.83 (0.77, 0.89) |
| 90-96 | 0.50 (0.48, 0.53) | 0.89 (0.84, 0.95) | 0.44 (0.41, 0.48) | 0.90 (0.80, 1.01) | 0.53 (0.51, 0.56) | 0.89 (0.83, 0.96) |
| 84-90 | 0.49 (0.47, 0.52) | 0.84 (0.79, 0.89) | 0.44 (0.41, 0.48) | 0.86 (0.76, 0.96) | 0.52 (0.49, 0.55) | 0.83 (0.77, 0.89) |
| 78-84 | 0.51 (0.49, 0.53) | 0.87 (0.82, 0.93) | 0.45 (0.42, 0.49) | 0.87 (0.78, 0.98) | 0.54 (0.51, 0.57) | 0.87 (0.81, 0.94) |
| 72-78 | 0.52 (0.50, 0.54) | 0.85 (0.80, 0.91) | 0.44 (0.40, 0.47) | 0.90 (0.80, 1.01) | 0.56 (0.54, 0.59) | 0.84 (0.78, 0.90) |
| 66-72 | 0.56 (0.53, 0.58) | 0.96 (0.91, 1.02) | 0.49 (0.46, 0.53) | 0.81 (0.72, 0.92) | 0.59 (0.56, 0.62) | 1.02 (0.95, 1.09) |
| 60-66 | 0.56 (0.54, 0.58) | 0.88 (0.83, 0.94) | 0.49 (0.45, 0.53) | 0.91 (0.81, 1.02) | 0.60 (0.57, 0.63) | 0.87 (0.81, 0.93) |
| 54-60 | 0.60 (0.57, 0.62) | 0.96 (0.91, 1.02) | 0.52 (0.49, 0.56) | 0.95 (0.84, 1.06) | 0.63 (0.60, 0.67) | 0.97 (0.90, 1.04) |
| 48-54 | 0.60 (0.58, 0.63) | 0.94 (0.88, 1.00) | 0.52 (0.48, 0.56) | 0.95 (0.85, 1.06) | 0.65 (0.61, 0.68) | 0.94 (0.87, 1.01) |
| 42-48 | 0.66 (0.63, 0.69) | 0.97 (0.92, 1.03) | 0.61 (0.57, 0.65) | 0.94 (0.84, 1.06) | 0.68 (0.65, 0.72) | 0.99 (0.92, 1.06) |
| 36-42 | 0.69 (0.66, 0.72) | 0.99 (0.93, 1.05) | 0.61 (0.57, 0.66) | 1.00 (0.89, 1.12) | 0.73 (0.69, 0.76) | 0.98 (0.92, 1.05) |
| 30-36 | 0.73 (0.70, 0.75) | 1.09 (1.02, 1.15) | 0.67 (0.62, 0.71) | 1.03 (0.92, 1.16) | 0.75 (0.72, 0.79) | 1.11 (1.03, 1.18) |
| 24-30 | 0.81 (0.78, 0.84) | 1.00 (0.94, 1.06) | 0.75 (0.70, 0.80) | 0.96 (0.85, 1.07) | 0.84 (0.81, 0.88) | 1.02 (0.95, 1.09) |
| 18-24 | 0.93 (0.90, 0.97) | 1.06 (1.00, 1.12) | 0.84 (0.78, 0.89) | 1.00 (0.89, 1.12) | 0.98 (0.94, 1.02) | 1.08 (1.01, 1.16) |
| 12-18 | 1.15 (1.11, 1.19) | 1.05 (0.99, 1.12) | 1.05 (0.99, 1.12) | 1.02 (0.91, 1.14) | 1.20 (1.15, 1.25) | 1.07 (1.00, 1.15) |
| 6-12 | 1.42 (1.38, 1.47) | 1.08 (1.02, 1.15) | 1.40 (1.32, 1.48) | 1.07 (0.95, 1.19) | 1.44 (1.38, 1.49) | 1.09 (1.02, 1.17) |
| 0-6 | 1.00 (1.00, 1.00) | 1.00 (1.00, 1.00) | 1.00 (1.00, 1.00) | 1.00 (1.00, 1.00) | 1.00 (1.00, 1.00) | 1.00 (1.00, 1.00) |
| Referrals for imaging | | | | | | |
| 114-120 | 0.06 (0.05, 0.06) | 0.38 (0.34, 0.42) | 0.04 (0.04, 0.05) | 0.33 (0.26, 0.42) | 0.06 (0.06, 0.07) | 0.39 (0.35, 0.45) |
| 108-114 | 0.06 (0.06, 0.07) | 0.34 (0.30, 0.38) | 0.05 (0.04, 0.05) | 0.32 (0.25, 0.41) | 0.07 (0.06, 0.07) | 0.35 (0.31, 0.40) |
| 102-108 | 0.07 (0.06, 0.07) | 0.42 (0.38, 0.47) | 0.05 (0.05, 0.06) | 0.36 (0.29, 0.46) | 0.08 (0.07, 0.08) | 0.44 (0.39, 0.49) |
| 96-102 | 0.07 (0.07, 0.08) | 0.38 (0.34, 0.42) | 0.05 (0.04, 0.06) | 0.39 (0.31, 0.48) | 0.08 (0.08, 0.09) | 0.38 (0.33, 0.43) |
| 90-96 | 0.08 (0.07, 0.08) | 0.42 (0.38, 0.47) | 0.06 (0.05, 0.07) | 0.39 (0.31, 0.49) | 0.08 (0.08, 0.09) | 0.43 (0.38, 0.48) |
| 84-90 | 0.08 (0.08, 0.09) | 0.43 (0.38, 0.47) | 0.06 (0.05, 0.07) | 0.37 (0.30, 0.47) | 0.10 (0.09, 0.10) | 0.44 (0.39, 0.50) |
| 78-84 | 0.09 (0.09, 0.10) | 0.49 (0.44, 0.54) | 0.07 (0.06, 0.07) | 0.39 (0.31, 0.49) | 0.10 (0.10, 0.11) | 0.52 (0.47, 0.58) |
| 72-78 | 0.10 (0.09, 0.10) | 0.45 (0.40, 0.50) | 0.08 (0.07, 0.08) | 0.38 (0.31, 0.48) | 0.11 (0.10, 0.12) | 0.47 (0.42, 0.53) |
| 66-72 | 0.11 (0.10, 0.11) | 0.45 (0.41, 0.50) | 0.09 (0.08, 0.10) | 0.49 (0.40, 0.60) | 0.11 (0.11, 0.12) | 0.44 (0.39, 0.49) |
| 60-66 | 0.11 (0.11, 0.12) | 0.52 (0.47, 0.57) | 0.09 (0.08, 0.10) | 0.47 (0.38, 0.58) | 0.13 (0.12, 0.14) | 0.53 (0.47, 0.59) |
| 54-60 | 0.12 (0.12, 0.13) | 0.59 (0.53, 0.65) | 0.10 (0.09, 0.11) | 0.53 (0.43, 0.64) | 0.14 (0.13, 0.14) | 0.61 (0.54, 0.67) |
| 48-54 | 0.14 (0.13, 0.14) | 0.54 (0.49, 0.60) | 0.11 (0.10, 0.12) | 0.53 (0.43, 0.65) | 0.15 (0.14, 0.16) | 0.55 (0.49, 0.61) |
| 42-48 | 0.15 (0.14, 0.16) | 0.56 (0.51, 0.61) | 0.13 (0.12, 0.14) | 0.47 (0.38, 0.58) | 0.16 (0.15, 0.17) | 0.58 (0.52, 0.65) |
| 36-42 | 0.18 (0.17, 0.18) | 0.56 (0.51, 0.62) | 0.16 (0.15, 0.17) | 0.49 (0.39, 0.60) | 0.19 (0.18, 0.20) | 0.59 (0.53, 0.66) |
| 30-36 | 0.20 (0.19, 0.21) | 0.59 (0.54, 0.65) | 0.18 (0.17, 0.19) | 0.53 (0.43, 0.65) | 0.21 (0.20, 0.22) | 0.61 (0.55, 0.68) |
| 24-30 | 0.23 (0.23, 0.24) | 0.60 (0.55, 0.66) | 0.20 (0.19, 0.22) | 0.55 (0.45, 0.67) | 0.25 (0.24, 0.26) | 0.62 (0.56, 0.69) |
| 18-24 | 0.30 (0.29, 0.31) | 0.66 (0.60, 0.72) | 0.27 (0.25, 0.28) | 0.61 (0.50, 0.74) | 0.32 (0.31, 0.33) | 0.68 (0.61, 0.75) |
| 12-18 | 0.42 (0.41, 0.44) | 0.73 (0.67, 0.80) | 0.38 (0.36, 0.40) | 0.64 (0.53, 0.78) | 0.45 (0.43, 0.46) | 0.76 (0.69, 0.84) |
| 6-12 | 0.77 (0.76, 0.79) | 0.93 (0.85, 1.01) | 0.75 (0.72, 0.78) | 0.97 (0.82, 1.15) | 0.79 (0.77, 0.81) | 0.91 (0.83, 1.00) |
| 0-6 | 1.00 (1.00, 1.00) | 1.00 (1.00, 1.00) | 1.00 (1.00, 1.00) | 1.00 (1.00, 1.00) | 1.00 (1.00, 1.00) | 1.00 (1.00, 1.00) |
| Referrals to pain clinic | | | | | | |
| 114-120 | 0.22 (0.19, 0.26) | 0.45 (0.35, 0.57) | 0.18 (0.12, 0.25) | 0.43 (0.26, 0.73) | 0.24 (0.20, 0.29) | 0.45 (0.35, 0.59) |
| 108-114 | 0.25 (0.22, 0.30) | 0.52 (0.42, 0.65) | 0.27 (0.20, 0.36) | 0.59 (0.37, 0.94) | 0.25 (0.21, 0.30) | 0.50 (0.39, 0.65) |
| 102-108 | 0.26 (0.22, 0.30) | 0.56 (0.45, 0.70) | 0.25 (0.18, 0.34) | 0.61 (0.38, 0.97) | 0.26 (0.22, 0.32) | 0.54 (0.42, 0.70) |
| 96-102 | 0.28 (0.24, 0.32) | 0.57 (0.45, 0.71) | 0.25 (0.18, 0.34) | 0.76 (0.49, 1.18) | 0.29 (0.24, 0.34) | 0.51 (0.40, 0.66) |
| 90-96 | 0.31 (0.26, 0.35) | 0.54 (0.43, 0.68) | 0.30 (0.22, 0.40) | 0.39 (0.23, 0.67) | 0.31 (0.26, 0.36) | 0.58 (0.46, 0.75) |
| 84-90 | 0.32 (0.28, 0.37) | 0.47 (0.38, 0.60) | 0.32 (0.24, 0.42) | 0.57 (0.35, 0.91) | 0.32 (0.27, 0.38) | 0.45 (0.35, 0.59) |
| 78-84 | 0.35 (0.30, 0.40) | 0.58 (0.46, 0.72) | 0.35 (0.26, 0.46) | 0.67 (0.43, 1.06) | 0.35 (0.30, 0.41) | 0.55 (0.43, 0.71) |
| 72-78 | 0.38 (0.33, 0.43) | 0.80 (0.66, 0.98) | 0.41 (0.31, 0.53) | 1.09 (0.73, 1.62) | 0.37 (0.31, 0.43) | 0.73 (0.58, 0.92) |
| 66-72 | 0.42 (0.37, 0.47) | 0.64 (0.52, 0.79) | 0.42 (0.32, 0.54) | 0.63 (0.40, 1.00) | 0.42 (0.36, 0.48) | 0.64 (0.51, 0.81) |
| 60-66 | 0.43 (0.38, 0.49) | 0.68 (0.56, 0.84) | 0.39 (0.30, 0.50) | 0.70 (0.44, 1.09) | 0.44 (0.38, 0.51) | 0.68 (0.54, 0.86) |
| 54-60 | 0.47 (0.41, 0.53) | 0.72 (0.59, 0.89) | 0.46 (0.36, 0.59) | 0.72 (0.46, 1.12) | 0.47 (0.41, 0.54) | 0.72 (0.57, 0.91) |
| 48-54 | 0.51 (0.45, 0.57) | 0.74 (0.61, 0.91) | 0.49 (0.39, 0.63) | 0.83 (0.54, 1.27) | 0.51 (0.45, 0.59) | 0.72 (0.57, 0.91) |
| 42-48 | 0.51 (0.45, 0.57) | 0.79 (0.64, 0.96) | 0.40 (0.31, 0.51) | 0.78 (0.51, 1.21) | 0.54 (0.47, 0.62) | 0.79 (0.63, 0.98) |
| 36-42 | 0.61 (0.54, 0.68) | 1.07 (0.89, 1.29) | 0.56 (0.44, 0.70) | 1.22 (0.82, 1.80) | 0.62 (0.55, 0.71) | 1.03 (0.84, 1.27) |
| 30-36 | 0.65 (0.58, 0.72) | 0.84 (0.69, 1.03) | 0.59 (0.47, 0.74) | 0.83 (0.54, 1.27) | 0.67 (0.59, 0.76) | 0.85 (0.68, 1.06) |
| 24-30 | 0.69 (0.62, 0.77) | 0.90 (0.74, 1.09) | 0.67 (0.54, 0.84) | 1.22 (0.82, 1.80) | 0.70 (0.61, 0.79) | 0.82 (0.65, 1.02) |
| 18-24 | 0.74 (0.67, 0.82) | 0.82 (0.67, 1.00) | 0.66 (0.53, 0.83) | 0.83 (0.54, 1.27) | 0.77 (0.68, 0.87) | 0.82 (0.65, 1.02) |
| 12-18 | 0.78 (0.70, 0.87) | 1.09 (0.91, 1.31) | 0.72 (0.58, 0.89) | 1.28 (0.87, 1.89) | 0.80 (0.71, 0.91) | 1.04 (0.84, 1.28) |
| 6-12 | 0.99 (0.90, 1.09) | 1.13 (0.94, 1.36) | 0.90 (0.74, 1.11) | 1.13 (0.76, 1.68) | 1.02 (0.91, 1.14) | 1.13 (0.92, 1.39) |
| 0-6 | 1.00 (1.00, 1.00) | 1.00 (1.00, 1.00) | 1.00 (1.00, 1.00) | 1.00 (1.00, 1.00) | 1.00 (1.00, 1.00) | 1.00 (1.00, 1.00) |
| Sick leave | | | | | | |
| 114-120 | 0.67 (0.65, 0.69) | 1.29 (1.23, 1.35) | 0.59 (0.55, 0.62) | 1.31 (1.20, 1.42) | 0.72 (0.69, 0.75) | 1.28 (1.21, 1.36) |
| 108-114 | 0.68 (0.66, 0.71) | 1.28 (1.23, 1.35) | 0.61 (0.57, 0.64) | 1.39 (1.28, 1.51) | 0.73 (0.70, 0.76) | 1.23 (1.16, 1.31) |
| 102-108 | 0.71 (0.68, 0.73) | 1.32 (1.26, 1.38) | 0.61 (0.57, 0.64) | 1.40 (1.29, 1.51) | 0.77 (0.74, 0.80) | 1.28 (1.21, 1.36) |
| 96-102 | 0.68 (0.66, 0.70) | 1.27 (1.22, 1.34) | 0.61 (0.57, 0.64) | 1.35 (1.25, 1.47) | 0.73 (0.69, 0.76) | 1.24 (1.17, 1.31) |
| 90-96 | 0.70 (0.67, 0.72) | 1.29 (1.23, 1.36) | 0.62 (0.59, 0.66) | 1.37 (1.26, 1.49) | 0.75 (0.71, 0.78) | 1.26 (1.19, 1.33) |
| 84-90 | 0.71 (0.68, 0.73) | 1.26 (1.20, 1.32) | 0.62 (0.59, 0.66) | 1.38 (1.27, 1.49) | 0.76 (0.73, 0.79) | 1.20 (1.14, 1.28) |
| 78-84 | 0.70 (0.68, 0.73) | 1.27 (1.21, 1.33) | 0.62 (0.59, 0.66) | 1.30 (1.19, 1.41) | 0.76 (0.72, 0.79) | 1.26 (1.19, 1.33) |
| 72-78 | 0.69 (0.67, 0.72) | 1.24 (1.18, 1.30) | 0.59 (0.56, 0.63) | 1.29 (1.19, 1.41) | 0.76 (0.73, 0.79) | 1.21 (1.14, 1.28) |
| 66-72 | 0.71 (0.69, 0.74) | 1.31 (1.25, 1.38) | 0.62 (0.58, 0.66) | 1.37 (1.26, 1.48) | 0.77 (0.74, 0.80) | 1.29 (1.22, 1.37) |
| 60-66 | 0.70 (0.68, 0.73) | 1.24 (1.19, 1.30) | 0.63 (0.60, 0.67) | 1.31 (1.21, 1.42) | 0.75 (0.71, 0.78) | 1.21 (1.14, 1.28) |
| 54-60 | 0.72 (0.69, 0.74) | 1.29 (1.23, 1.35) | 0.62 (0.58, 0.65) | 1.34 (1.24, 1.46) | 0.78 (0.75, 0.82) | 1.26 (1.19, 1.34) |
| 48-54 | 0.72 (0.70, 0.75) | 1.21 (1.16, 1.27) | 0.63 (0.59, 0.66) | 1.24 (1.14, 1.35) | 0.78 (0.75, 0.82) | 1.20 (1.13, 1.27) |
| 42-48 | 0.71 (0.69, 0.74) | 1.22 (1.16, 1.28) | 0.61 (0.57, 0.64) | 1.19 (1.10, 1.30) | 0.78 (0.75, 0.81) | 1.23 (1.16, 1.31) |
| 36-42 | 0.71 (0.68, 0.73) | 1.15 (1.09, 1.20) | 0.61 (0.58, 0.65) | 1.15 (1.05, 1.25) | 0.76 (0.73, 0.80) | 1.15 (1.08, 1.21) |
| 30-36 | 0.71 (0.69, 0.73) | 1.21 (1.15, 1.26) | 0.64 (0.60, 0.68) | 1.18 (1.08, 1.28) | 0.75 (0.72, 0.79) | 1.22 (1.15, 1.29) |
| 24-30 | 0.71 (0.68, 0.73) | 1.09 (1.04, 1.14) | 0.63 (0.59, 0.67) | 1.08 (0.99, 1.18) | 0.76 (0.73, 0.79) | 1.09 (1.03, 1.16) |
| 18-24 | 0.72 (0.70, 0.75) | 1.15 (1.10, 1.21) | 0.65 (0.61, 0.68) | 1.18 (1.08, 1.28) | 0.77 (0.74, 0.81) | 1.14 (1.07, 1.21) |
| 12-18 | 0.75 (0.73, 0.78) | 1.04 (0.99, 1.10) | 0.69 (0.65, 0.73) | 1.07 (0.98, 1.16) | 0.79 (0.76, 0.83) | 1.03 (0.97, 1.10) |
| 6-12 | 0.83 (0.80, 0.85) | 1.05 (1.00, 1.10) | 0.78 (0.74, 0.83) | 1.08 (1.00, 1.18) | 0.85 (0.82, 0.89) | 1.03 (0.97, 1.10) |
| 0-6 | 1.00 (1.00, 1.00) | 1.00 (1.00, 1.00) | 1.00 (1.00, 1.00) | 1.00 (1.00, 1.00) | 1.00 (1.00, 1.00) | 1.00 (1.00, 1.00) |

**Supplementary Table 3**

Conditional odds ratios and 95% CIs for primary THR associated with pharmacological and non-pharmacological care strategies within each 6-month period adjusted for geographical region, ethnicity, neighbourhood deprivation, comorbidity, BMI, smoking status, drinking status, and hip injury.

| Months prior to index date | Oral NSAIDs | | | | Topical NSAIDs | | |
| --- | --- | --- | --- | --- | --- | --- | --- |
|  | Overall | Male | Female | | Overall | Male | Female |
|  | OR (95% CI) | OR (95% CI) | OR (95% CI) | | OR (95% CI) | OR (95% CI) | OR (95% CI) |
| 114-120 | 1.51 (1.47, 1.55) | 1.52 (1.45, 1.61) | 1.50 (1.45, 1.55) | | 1.09 (1.03, 1.16) | 1.15 (1.02, 1.30) | 1.07 (1.00, 1.15) |
| 108-114 | 1.59 (1.55, 1.64) | 1.61 (1.53, 1.70) | 1.59 (1.53, 1.64) | | 1.12 (1.06, 1.19) | 1.11 (0.98, 1.25) | 1.13 (1.05, 1.20) |
| 102-108 | 1.60 (1.55, 1.64) | 1.62 (1.54, 1.71) | 1.59 (1.54, 1.64) | | 1.16 (1.09, 1.22) | 1.30 (1.16, 1.46) | 1.11 (1.04, 1.19) |
| 96-102 | 1.64 (1.59, 1.68) | 1.63 (1.55, 1.71) | 1.64 (1.59, 1.70) | | 1.17 (1.11, 1.24) | 1.20 (1.08, 1.34) | 1.16 (1.09, 1.24) |
| 90-96 | 1.69 (1.64, 1.74) | 1.73 (1.64, 1.82) | 1.68 (1.62, 1.73) | | 1.12 (1.07, 1.19) | 1.17 (1.05, 1.30) | 1.11 (1.04, 1.18) |
| 84-90 | 1.75 (1.70, 1.80) | 1.75 (1.66, 1.84) | 1.75 (1.69, 1.81) | | 1.23 (1.16, 1.29) | 1.17 (1.06, 1.30) | 1.25 (1.17, 1.33) |
| 78-84 | 1.73 (1.68, 1.78) | 1.79 (1.70, 1.89) | 1.71 (1.65, 1.76) | | 1.24 (1.18, 1.31) | 1.29 (1.17, 1.42) | 1.23 (1.16, 1.31) |
| 72-78 | 1.83 (1.78, 1.89) | 1.89 (1.79, 1.99) | 1.81 (1.75, 1.87) | | 1.27 (1.21, 1.33) | 1.30 (1.18, 1.43) | 1.26 (1.19, 1.34) |
| 66-72 | 1.82 (1.77, 1.87) | 1.89 (1.79, 1.99) | 1.79 (1.73, 1.85) | | 1.28 (1.22, 1.34) | 1.27 (1.16, 1.40) | 1.28 (1.21, 1.35) |
| 60-66 | 1.89 (1.84, 1.95) | 1.95 (1.85, 2.06) | 1.87 (1.80, 1.94) | | 1.34 (1.28, 1.40) | 1.35 (1.23, 1.48) | 1.34 (1.27, 1.42) |
| 54-60 | 1.93 (1.88, 1.99) | 2.03 (1.92, 2.14) | 1.89 (1.83, 1.96) | | 1.32 (1.26, 1.38) | 1.38 (1.26, 1.51) | 1.30 (1.23, 1.37) |
| 48-54 | 2.02 (1.96, 2.08) | 2.08 (1.97, 2.20) | 1.99 (1.92, 2.06) | | 1.42 (1.35, 1.48) | 1.39 (1.27, 1.51) | 1.43 (1.36, 1.51) |
| 42-48 | 2.09 (2.03, 2.16) | 2.21 (2.10, 2.34) | 2.04 (1.97, 2.12) | | 1.41 (1.35, 1.47) | 1.48 (1.36, 1.62) | 1.39 (1.32, 1.46) |
| 36-42 | 2.18 (2.12, 2.25) | 2.39 (2.26, 2.52) | 2.10 (2.03, 2.18) | | 1.58 (1.51, 1.65) | 1.69 (1.55, 1.84) | 1.55 (1.47, 1.63) |
| 30-36 | 2.35 (2.28, 2.42) | 2.50 (2.36, 2.64) | 2.29 (2.21, 2.38) | | 1.53 (1.46, 1.59) | 1.67 (1.54, 1.82) | 1.48 (1.41, 1.55) |
| 24-30 | 2.53 (2.45, 2.61) | 2.84 (2.68, 3.01) | 2.41 (2.32, 2.50) | | 1.63 (1.57, 1.70) | 1.75 (1.62, 1.89) | 1.59 (1.51, 1.67) |
| 18-24 | 2.73 (2.64, 2.81) | 3.03 (2.86, 3.21) | 2.61 (2.51, 2.71) | | 1.70 (1.64, 1.77) | 1.85 (1.71, 2.00) | 1.65 (1.58, 1.73) |
| 12-18 | 3.21 (3.11, 3.31) | 3.57 (3.36, 3.79) | 3.06 (2.95, 3.18) | | 1.91 (1.84, 1.99) | 2.10 (1.94, 2.26) | 1.85 (1.77, 1.94) |
| 6-12 | 3.99 (3.86, 4.12) | 4.63 (4.35, 4.93) | 3.74 (3.60, 3.89) | | 2.15 (2.07, 2.24) | 2.45 (2.27, 2.64) | 2.05 (1.96, 2.15) |
| 0-6 | 4.46 (4.32, 4.62) | 5.13 (4.81, 5.46) | 4.20 (4.03, 4.37) | | 2.10 (2.02, 2.19) | 2.31 (2.15, 2.49) | 2.02 (1.93, 2.12) |
|  | Paracetamol | | | | Weak opioids | | |
| 114-120 | 1.06 (1.02, 1.11) | 1.09 (1.00, 1.19) | 1.05 (1.00, 1.11) | | 1.39 (1.33, 1.45) | 1.32 (1.21, 1.43) | 1.42 (1.35, 1.49) |
| 108-114 | 1.06 (1.02, 1.11) | 1.05 (0.96, 1.14) | 1.07 (1.02, 1.12) | | 1.43 (1.37, 1.49) | 1.34 (1.23, 1.46) | 1.47 (1.40, 1.54) |
| 102-108 | 1.11 (1.06, 1.15) | 1.11 (1.02, 1.20) | 1.11 (1.05, 1.16) | | 1.46 (1.40, 1.53) | 1.38 (1.27, 1.51) | 1.50 (1.43, 1.57) |
| 96-102 | 1.14 (1.09, 1.18) | 1.16 (1.07, 1.25) | 1.13 (1.08, 1.18) | | 1.51 (1.45, 1.58) | 1.46 (1.34, 1.59) | 1.53 (1.46, 1.61) |
| 90-96 | 1.18 (1.13, 1.22) | 1.21 (1.12, 1.31) | 1.16 (1.11, 1.22) | | 1.52 (1.46, 1.59) | 1.51 (1.39, 1.65) | 1.52 (1.45, 1.60) |
| 84-90 | 1.24 (1.19, 1.29) | 1.25 (1.16, 1.35) | 1.23 (1.18, 1.29) | | 1.55 (1.48, 1.62) | 1.46 (1.34, 1.59) | 1.58 (1.51, 1.66) |
| 78-84 | 1.27 (1.22, 1.31) | 1.27 (1.18, 1.37) | 1.26 (1.21, 1.32) | | 1.53 (1.47, 1.60) | 1.50 (1.38, 1.64) | 1.55 (1.47, 1.63) |
| 72-78 | 1.30 (1.25, 1.35) | 1.34 (1.25, 1.44) | 1.28 (1.23, 1.34) | | 1.55 (1.49, 1.62) | 1.66 (1.52, 1.81) | 1.52 (1.45, 1.60) |
| 66-72 | 1.34 (1.29, 1.38) | 1.41 (1.32, 1.52) | 1.31 (1.26, 1.37) | | 1.56 (1.49, 1.63) | 1.56 (1.43, 1.70) | 1.56 (1.48, 1.64) |
| 60-66 | 1.40 (1.36, 1.45) | 1.41 (1.32, 1.51) | 1.40 (1.35, 1.46) | | 1.62 (1.55, 1.70) | 1.80 (1.65, 1.96) | 1.57 (1.49, 1.65) |
| 54-60 | 1.43 (1.39, 1.48) | 1.49 (1.39, 1.59) | 1.42 (1.36, 1.48) | | 1.62 (1.55, 1.69) | 1.69 (1.55, 1.85) | 1.60 (1.52, 1.68) |
| 48-54 | 1.49 (1.44, 1.55) | 1.55 (1.45, 1.65) | 1.48 (1.42, 1.54) | | 1.72 (1.64, 1.79) | 1.87 (1.72, 2.05) | 1.67 (1.59, 1.76) |
| 42-48 | 1.58 (1.52, 1.63) | 1.66 (1.55, 1.77) | 1.55 (1.49, 1.61) | | 1.70 (1.63, 1.78) | 1.89 (1.73, 2.05) | 1.64 (1.56, 1.73) |
| 36-42 | 1.64 (1.59, 1.70) | 1.75 (1.64, 1.86) | 1.61 (1.55, 1.67) | | 1.79 (1.71, 1.87) | 2.04 (1.88, 2.23) | 1.71 (1.62, 1.80) |
| 30-36 | 1.72 (1.67, 1.78) | 1.79 (1.68, 1.91) | 1.70 (1.64, 1.77) | | 1.86 (1.78, 1.94) | 2.09 (1.92, 2.27) | 1.79 (1.70, 1.88) |
| 24-30 | 1.87 (1.81, 1.93) | 1.93 (1.82, 2.06) | 1.85 (1.78, 1.92) | | 2.02 (1.94, 2.11) | 2.33 (2.14, 2.54) | 1.93 (1.84, 2.03) |
| 18-24 | 2.02 (1.96, 2.09) | 2.11 (1.98, 2.24) | 1.99 (1.92, 2.07) | | 2.12 (2.03, 2.21) | 2.38 (2.19, 2.58) | 2.04 (1.94, 2.14) |
| 12-18 | 2.31 (2.24, 2.39) | 2.49 (2.34, 2.65) | 2.25 (2.16, 2.34) | | 2.49 (2.39, 2.60) | 2.83 (2.61, 3.07) | 2.40 (2.28, 2.51) |
| 6-12 | 2.73 (2.64, 2.82) | 3.03 (2.85, 3.22) | 2.62 (2.53, 2.72) | | 3.19 (3.07, 3.32) | 3.85 (3.55, 4.16) | 2.99 (2.86, 3.13) |
| 0-6 | 3.21 (3.11, 3.31) | 3.65 (3.43, 3.88) | 3.05 (2.94, 3.17) | | 4.00 (3.84, 4.16) | 4.97 (4.59, 5.37) | 3.69 (3.52, 3.86) |
|  | Strong opioids | | | | Antidepressants | | |
| 114-120 | 1.35 (1.26, 1.44) | 1.36 (1.20, 1.55) | 1.35 (1.26, 1.44) | | 1.10 (1.06, 1.13) | 0.96 (0.90, 1.03) | 1.14 (1.10, 1.18) |
| 108-114 | 1.39 (1.31, 1.48) | 1.36 (1.20, 1.53) | 1.39 (1.31, 1.48) | | 1.11 (1.07, 1.14) | 0.98 (0.92, 1.05) | 1.14 (1.11, 1.19) |
| 102-108 | 1.42 (1.34, 1.51) | 1.44 (1.28, 1.61) | 1.42 (1.34, 1.51) | | 1.12 (1.08, 1.15) | 0.98 (0.92, 1.05) | 1.16 (1.12, 1.20) |
| 96-102 | 1.47 (1.39, 1.56) | 1.49 (1.33, 1.67) | 1.47 (1.39, 1.56) | | 1.12 (1.09, 1.15) | 1.02 (0.95, 1.09) | 1.15 (1.11, 1.19) |
| 90-96 | 1.48 (1.40, 1.57) | 1.44 (1.29, 1.61) | 1.48 (1.40, 1.57) | | 1.14 (1.11, 1.18) | 1.03 (0.96, 1.10) | 1.18 (1.14, 1.22) |
| 84-90 | 1.51 (1.42, 1.59) | 1.51 (1.35, 1.68) | 1.51 (1.42, 1.59) | | 1.15 (1.11, 1.18) | 1.05 (0.98, 1.12) | 1.17 (1.13, 1.21) |
| 78-84 | 1.54 (1.46, 1.63) | 1.60 (1.44, 1.79) | 1.54 (1.46, 1.63) | | 1.16 (1.13, 1.20) | 1.06 (0.99, 1.13) | 1.19 (1.15, 1.23) |
| 72-78 | 1.61 (1.52, 1.69) | 1.54 (1.39, 1.71) | 1.61 (1.52, 1.69) | | 1.17 (1.14, 1.21) | 1.06 (0.99, 1.13) | 1.20 (1.16, 1.25) |
| 66-72 | 1.62 (1.54, 1.70) | 1.63 (1.47, 1.81) | 1.62 (1.54, 1.70) | | 1.18 (1.15, 1.22) | 1.07 (1.00, 1.14) | 1.22 (1.18, 1.26) |
| 60-66 | 1.70 (1.62, 1.79) | 1.63 (1.47, 1.80) | 1.70 (1.62, 1.79) | | 1.19 (1.16, 1.23) | 1.09 (1.02, 1.16) | 1.22 (1.18, 1.26) |
| 54-60 | 1.73 (1.65, 1.82) | 1.63 (1.48, 1.80) | 1.73 (1.65, 1.82) | | 1.22 (1.19, 1.26) | 1.11 (1.05, 1.19) | 1.26 (1.21, 1.30) |
| 48-54 | 1.80 (1.72, 1.90) | 1.72 (1.56, 1.89) | 1.80 (1.72, 1.90) | | 1.24 (1.20, 1.28) | 1.11 (1.04, 1.18) | 1.28 (1.24, 1.32) |
| 42-48 | 1.86 (1.77, 1.95) | 1.80 (1.64, 1.98) | 1.86 (1.77, 1.95) | | 1.24 (1.20, 1.28) | 1.14 (1.07, 1.21) | 1.27 (1.23, 1.31) |
| 36-42 | 2.01 (1.91, 2.11) | 2.05 (1.86, 2.26) | 2.01 (1.91, 2.11) | | 1.28 (1.24, 1.31) | 1.19 (1.12, 1.27) | 1.30 (1.26, 1.35) |
| 30-36 | 2.06 (1.97, 2.16) | 2.30 (2.09, 2.53) | 2.06 (1.97, 2.16) | | 1.30 (1.26, 1.34) | 1.22 (1.15, 1.30) | 1.32 (1.28, 1.37) |
| 24-30 | 2.20 (2.09, 2.30) | 2.32 (2.11, 2.55) | 2.20 (2.09, 2.30) | | 1.34 (1.30, 1.37) | 1.26 (1.19, 1.34) | 1.36 (1.32, 1.40) |
| 18-24 | 2.39 (2.29, 2.51) | 2.58 (2.35, 2.84) | 2.39 (2.29, 2.51) | | 1.39 (1.35, 1.43) | 1.30 (1.22, 1.38) | 1.41 (1.37, 1.46) |
| 12-18 | 2.84 (2.72, 2.98) | 3.20 (2.92, 3.51) | 2.84 (2.72, 2.98) | | 1.48 (1.44, 1.52) | 1.40 (1.32, 1.49) | 1.50 (1.45, 1.55) |
| 6-12 | 3.64 (3.48, 3.81) | 4.17 (3.81, 4.56) | 3.64 (3.48, 3.81) | | 1.58 (1.54, 1.63) | 1.51 (1.43, 1.61) | 1.60 (1.55, 1.66) |
| 0-6 | 4.98 (4.77, 5.21) | 5.66 (5.19, 6.17) | 4.98 (4.77, 5.21) | | 1.62 (1.57, 1.66) | 1.60 (1.51, 1.70) | 1.62 (1.57, 1.68) |
|  | Antiepileptics | | | | Bisphosphonates | | |
| 114-120 | 1.05 (0.98, 1.13) | 0.94 (0.83, 1.06) | 1.10 (1.01, 1.20) | | 0.85 (0.79, 0.92) | 0.61 (0.47, 0.80) | 0.88 (0.81, 0.95) |
| 108-114 | 1.05 (0.99, 1.13) | 0.95 (0.84, 1.07) | 1.11 (1.02, 1.20) | | 0.88 (0.81, 0.94) | 0.54 (0.41, 0.70) | 0.91 (0.85, 0.99) |
| 102-108 | 1.08 (1.02, 1.16) | 0.96 (0.85, 1.08) | 1.14 (1.06, 1.24) | | 0.88 (0.82, 0.94) | 0.59 (0.46, 0.75) | 0.91 (0.84, 0.98) |
| 96-102 | 1.05 (0.98, 1.12) | 0.94 (0.84, 1.06) | 1.10 (1.02, 1.19) | | 0.91 (0.85, 0.97) | 0.65 (0.51, 0.82) | 0.93 (0.87, 1.00) |
| 90-96 | 1.05 (0.99, 1.12) | 0.93 (0.83, 1.04) | 1.11 (1.03, 1.20) | | 0.94 (0.88, 1.00) | 0.75 (0.59, 0.93) | 0.96 (0.89, 1.02) |
| 84-90 | 1.08 (1.02, 1.15) | 0.94 (0.85, 1.05) | 1.14 (1.06, 1.23) | | 0.94 (0.88, 1.00) | 0.74 (0.59, 0.92) | 0.96 (0.90, 1.03) |
| 78-84 | 1.13 (1.07, 1.20) | 1.03 (0.93, 1.15) | 1.18 (1.10, 1.27) | | 0.96 (0.90, 1.02) | 0.76 (0.61, 0.94) | 0.98 (0.92, 1.04) |
| 72-78 | 1.16 (1.09, 1.23) | 1.05 (0.95, 1.17) | 1.21 (1.13, 1.30) | | 0.96 (0.90, 1.02) | 0.71 (0.58, 0.88) | 0.98 (0.92, 1.05) |
| 66-72 | 1.18 (1.12, 1.25) | 1.09 (0.98, 1.21) | 1.22 (1.14, 1.31) | | 0.98 (0.92, 1.04) | 0.71 (0.57, 0.87) | 1.01 (0.95, 1.07) |
| 60-66 | 1.24 (1.18, 1.32) | 1.19 (1.08, 1.32) | 1.26 (1.18, 1.35) | | 1.01 (0.96, 1.07) | 0.83 (0.68, 1.01) | 1.03 (0.97, 1.09) |
| 54-60 | 1.27 (1.21, 1.35) | 1.25 (1.13, 1.38) | 1.28 (1.20, 1.37) | | 1.03 (0.98, 1.10) | 0.88 (0.73, 1.07) | 1.05 (0.99, 1.12) |
| 48-54 | 1.29 (1.23, 1.37) | 1.21 (1.10, 1.34) | 1.33 (1.25, 1.42) | | 1.05sick (0.99, 1.11) | 0.85 (0.70, 1.02) | 1.07 (1.01, 1.14) |
| 42-48 | 1.37 (1.30, 1.45) | 1.26 (1.14, 1.39) | 1.42 (1.33, 1.51) | | 1.09 (1.03, 1.15) | 0.95 (0.79, 1.13) | 1.10 (1.04, 1.17) |
| 36-42 | 1.38 (1.31, 1.45) | 1.24 (1.13, 1.37) | 1.45 (1.36, 1.54) | | 1.12 (1.06, 1.18) | 0.97 (0.81, 1.16) | 1.13 (1.07, 1.20) |
| 30-36 | 1.47 (1.39, 1.54) | 1.42 (1.29, 1.56) | 1.49 (1.40, 1.58) | | 1.15 (1.09, 1.22) | 1.11 (0.93, 1.32) | 1.16 (1.09, 1.23) |
| 24-30 | 1.50 (1.42, 1.57) | 1.41 (1.29, 1.54) | 1.54 (1.45, 1.63) | | 1.22 (1.16, 1.29) | 1.13 (0.95, 1.35) | 1.23 (1.16, 1.30) |
| 18-24 | 1.60 (1.52, 1.68) | 1.53 (1.40, 1.67) | 1.63 (1.54, 1.73) | | 1.23 (1.17, 1.30) | 1.26 (1.06, 1.50) | 1.23 (1.16, 1.30) |
| 12-18 | 1.68 (1.61, 1.77) | 1.62 (1.48, 1.77) | 1.71 (1.62, 1.81) | | 1.28 (1.21, 1.35) | 1.36 (1.15, 1.60) | 1.27 (1.20, 1.34) |
| 6-12 | 1.84 (1.76, 1.92) | 1.80 (1.65, 1.96) | 1.86 (1.76, 1.96) | | 1.33 (1.26, 1.40) | 1.49 (1.26, 1.75) | 1.31 (1.24, 1.39) |
| 0-6 | 1.95 (1.87, 2.04) | 1.89 (1.74, 2.06) | 1.98 (1.88, 2.09) | | 1.41 (1.34, 1.49) | 1.57 (1.34, 1.84) | 1.40 (1.32, 1.48) |
|  | All corticosteroids | | | | Systemic steroids | | |
| 114-120 | 1.01 (0.98, 1.03) | 0.95 (0.91, 0.99) | 1.04 (1.00, 1.07) | | 1.03 (0.97, 1.09) | 0.96 (0.86, 1.07) | 1.05 (0.98, 1.13) |
| 108-114 | 1.06 (1.03, 1.09) | 0.99 (0.95, 1.04) | 1.09 (1.06, 1.13) | | 1.08 (1.02, 1.14) | 0.98 (0.89, 1.09) | 1.11 (1.04, 1.19) |
| 102-108 | 1.05 (1.02, 1.07) | 0.99 (0.95, 1.03) | 1.07 (1.04, 1.11) | | 1.09 (1.03, 1.15) | 0.95 (0.86, 1.06) | 1.15 (1.08, 1.23) |
| 96-102 | 1.08 (1.05, 1.11) | 1.02 (0.98, 1.07) | 1.11 (1.08, 1.15) | | 1.15 (1.09, 1.22) | 1.01 (0.91, 1.12) | 1.22 (1.14, 1.30) |
| 90-96 | 1.06 (1.03, 1.09) | 1.02 (0.97, 1.06) | 1.08 (1.05, 1.11) | | 1.10 (1.04, 1.16) | 0.97 (0.88, 1.07) | 1.15 (1.08, 1.23) |
| 84-90 | 1.12 (1.10, 1.15) | 1.09 (1.05, 1.14) | 1.14 (1.11, 1.17) | | 1.14 (1.08, 1.20) | 1.04 (0.94, 1.15) | 1.18 (1.11, 1.26) |
| 78-84 | 1.08 (1.05, 1.10) | 1.05 (1.01, 1.09) | 1.09 (1.06, 1.13) | | 1.11 (1.05, 1.17) | 1.05 (0.95, 1.16) | 1.14 (1.07, 1.21) |
| 72-78 | 1.15 (1.12, 1.18) | 1.09 (1.05, 1.14) | 1.18 (1.14, 1.21) | | 1.19 (1.13, 1.25) | 1.05 (0.96, 1.16) | 1.26 (1.18, 1.34) |
| 66-72 | 1.10 (1.08, 1.13) | 1.07 (1.03, 1.11) | 1.12 (1.09, 1.16) | | 1.17 (1.11, 1.23) | 1.06 (0.97, 1.17) | 1.21 (1.14, 1.29) |
| 60-66 | 1.20 (1.17, 1.23) | 1.18 (1.13, 1.23) | 1.21 (1.17, 1.24) | | 1.26 (1.20, 1.33) | 1.17 (1.06, 1.28) | 1.30 (1.22, 1.38) |
| 54-60 | 1.16 (1.13, 1.19) | 1.13 (1.08, 1.18) | 1.17 (1.14, 1.21) | | 1.23 (1.17, 1.29) | 1.10 (1.00, 1.21) | 1.29 (1.22, 1.36) |
| 48-54 | 1.22 (1.19, 1.25) | 1.20 (1.15, 1.25) | 1.23 (1.19, 1.27) | | 1.32 (1.25, 1.38) | 1.24 (1.13, 1.35) | 1.35 (1.28, 1.43) |
| 42-48 | 1.17 (1.14, 1.20) | 1.15 (1.10, 1.20) | 1.18 (1.14, 1.21) | | 1.29 (1.23, 1.36) | 1.22 (1.11, 1.33) | 1.32 (1.25, 1.40) |
| 36-42 | 1.24 (1.21, 1.27) | 1.21 (1.16, 1.26) | 1.25 (1.21, 1.29) | | 1.42 (1.35, 1.49) | 1.28 (1.17, 1.40) | 1.48 (1.39, 1.56) |
| 30-36 | 1.23 (1.20, 1.26) | 1.24 (1.19, 1.30) | 1.22 (1.19, 1.26) | | 1.45 (1.38, 1.52) | 1.37 (1.25, 1.49) | 1.48 (1.40, 1.57) |
| 24-30 | 1.29 (1.26, 1.33) | 1.30 (1.24, 1.35) | 1.29 (1.25, 1.33) | | 1.54 (1.47, 1.62) | 1.49 (1.36, 1.62) | 1.57 (1.49, 1.66) |
| 18-24 | 1.28 (1.25, 1.31) | 1.27 (1.22, 1.33) | 1.29 (1.25, 1.33) | | 1.62 (1.55, 1.69) | 1.48 (1.36, 1.61) | 1.69 (1.60, 1.78) |
| 12-18 | 1.37 (1.33, 1.40) | 1.40 (1.34, 1.46) | 1.35 (1.31, 1.39) | | 1.77 (1.70, 1.86) | 1.72 (1.58, 1.87) | 1.80 (1.70, 1.89) |
| 6-12 | 1.37 (1.33, 1.40) | 1.39 (1.33, 1.45) | 1.35 (1.32, 1.39) | | 1.87 (1.79, 1.95) | 1.84 (1.70, 1.99) | 1.88 (1.79, 1.98) |
| 0-6 | 1.38 (1.35, 1.41) | 1.47 (1.40, 1.53) | 1.34 (1.30, 1.38) | | 1.58 (1.50, 1.65) | 1.62 (1.49, 1.76) | 1.56 (1.48, 1.65) |
|  | GP visits | | | | Referrals for physiotherapy | | |
| 114-120 | 1.07 (0.99, 1.15) | 1.11 (0.97, 1.26) | 1.04 (0.95, 1.14) | | 1.41 (1.30, 1.53) | 1.53 (1.30, 1.80) | 1.36 (1.24, 1.50) |
| 108-114 | 1.07 (0.99, 1.15) | 0.97 (0.85, 1.11) | 1.11 (1.01, 1.22) | | 1.39 (1.28, 1.51) | 1.46 (1.24, 1.71) | 1.36 (1.24, 1.50) |
| 102-108 | 1.15 (1.06, 1.24) | 1.11 (0.96, 1.27) | 1.15 (1.05, 1.26) | | 1.32 (1.22, 1.43) | 1.49 (1.28, 1.74) | 1.27 (1.16, 1.40) |
| 96-102 | 1.02 (0.95, 1.11) | 0.90 (0.78, 1.03) | 1.08 (0.99, 1.19) | | 1.33 (1.23, 1.44) | 1.38 (1.18, 1.62) | 1.32 (1.20, 1.45) |
| 90-96 | 1.19 (1.10, 1.29) | 1.23 (1.07, 1.41) | 1.17 (1.07, 1.29) | | 1.37 (1.27, 1.47) | 1.44 (1.24, 1.67) | 1.34 (1.23, 1.46) |
| 84-90 | 1.14 (1.05, 1.23) | 1.14 (0.99, 1.31) | 1.13 (1.03, 1.24) | | 1.37 (1.27, 1.48) | 1.30 (1.12, 1.51) | 1.41 (1.28, 1.54) |
| 78-84 | 1.18 (1.09, 1.28) | 1.30 (1.12, 1.50) | 1.13 (1.02, 1.25) | | 1.39 (1.29, 1.50) | 1.45 (1.24, 1.68) | 1.37 (1.25, 1.50) |
| 72-78 | 1.15 (1.06, 1.25) | 1.11 (0.96, 1.29) | 1.17 (1.06, 1.30) | | 1.45 (1.34, 1.56) | 1.20 (1.04, 1.39) | 1.55 (1.42, 1.70) |
| 66-72 | 1.18 (1.09, 1.29) | 1.29 (1.10, 1.50) | 1.13 (1.02, 1.26) | | 1.42 (1.32, 1.53) | 1.76 (1.52, 2.04) | 1.32 (1.21, 1.43) |
| 60-66 | 1.16 (1.06, 1.26) | 1.22 (1.05, 1.42) | 1.13 (1.02, 1.25) | | 1.59 (1.47, 1.71) | 1.47 (1.28, 1.70) | 1.63 (1.50, 1.79) |
| 54-60 | 1.19 (1.09, 1.29) | 1.34 (1.15, 1.55) | 1.13 (1.02, 1.25) | | 1.47 (1.37, 1.58) | 1.46 (1.27, 1.68) | 1.47 (1.35, 1.61) |
| 48-54 | 1.22 (1.12, 1.33) | 1.22 (1.04, 1.42) | 1.23 (1.10, 1.37) | | 1.57 (1.46, 1.69) | 1.57 (1.36, 1.81) | 1.57 (1.44, 1.71) |
| 42-48 | 1.39 (1.27, 1.51) | 1.55 (1.33, 1.81) | 1.30 (1.17, 1.45) | | 1.62 (1.51, 1.74) | 1.66 (1.44, 1.91) | 1.60 (1.48, 1.74) |
| 36-42 | 1.25 (1.14, 1.36) | 1.23 (1.05, 1.43) | 1.27 (1.13, 1.42) | | 1.74 (1.62, 1.86) | 1.77 (1.55, 2.02) | 1.73 (1.59, 1.88) |
| 30-36 | 1.33 (1.22, 1.45) | 1.34 (1.14, 1.57) | 1.32 (1.18, 1.47) | | 1.65 (1.55, 1.77) | 1.90 (1.66, 2.17) | 1.58 (1.46, 1.70) |
| 24-30 | 1.34 (1.22, 1.47) | 1.38 (1.18, 1.63) | 1.32 (1.18, 1.47) | | 1.99 (1.86, 2.13) | 2.31 (2.02, 2.65) | 1.89 (1.74, 2.04) |
| 18-24 | 1.44 (1.31, 1.58) | 1.45 (1.24, 1.70) | 1.44 (1.29, 1.62) | | 2.18 (2.05, 2.33) | 2.46 (2.15, 2.81) | 2.12 (1.96, 2.28) |
| 12-18 | 1.55 (1.41, 1.70) | 1.69 (1.43, 2.00) | 1.48 (1.32, 1.66) | | 2.63 (2.47, 2.80) | 2.92 (2.58, 3.30) | 2.53 (2.35, 2.72) |
| 6-12 | 1.65 (1.50, 1.81) | 1.81 (1.53, 2.14) | 1.57 (1.40, 1.76) | | 3.29 (3.10, 3.50) | 4.06 (3.60, 4.57) | 3.05 (2.84, 3.27) |
| 0-6 | 1.88 (1.71, 2.08) | 2.10 (1.77, 2.50) | 1.80 (1.60, 2.03) | | 2.49 (2.33, 2.65) | 3.00 (2.65, 3.40) | 2.32 (2.15, 2.50) |
|  | Referrals for imaging | | | | Referrals to pain clinic | | |
| 114-120 | 1.63 (1.40, 1.91) | 2.19 (1.54, 3.12) | 1.51 (1.27, 1.80) | | 1.78 (1.42, 2.24) | 1.72 (1.05, 2.83) | 1.80 (1.39, 2.33) |
| 108-114 | 2.06 (1.76, 2.41) | 2.40 (1.70, 3.40) | 1.97 (1.65, 2.36) | | 1.80 (1.45, 2.23) | 2.10 (1.36, 3.22) | 1.70 (1.32, 2.19) |
| 102-108 | 1.76 (1.53, 2.03) | 2.55 (1.82, 3.57) | 1.65 (1.41, 1.94) | | 1.74 (1.41, 2.15) | 1.74 (1.15, 2.65) | 1.74 (1.36, 2.23) |
| 96-102 | 2.23 (1.92, 2.59) | 2.67 (1.89, 3.76) | 2.22 (1.87, 2.64) | | 1.73 (1.41, 2.14) | 1.40 (0.93, 2.11) | 1.87 (1.46, 2.39) |
| 90-96 | 1.95 (1.70, 2.24) | 2.24 (1.69, 2.97) | 1.86 (1.59, 2.19) | | 2.02 (1.64, 2.49) | 2.96 (1.86, 4.71) | 1.82 (1.44, 2.29) |
| 84-90 | 2.40 (2.09, 2.74) | 3.20 (2.31, 4.45) | 2.25 (1.93, 2.62) | | 2.33 (1.88, 2.89) | 2.45 (1.61, 3.73) | 2.29 (1.79, 2.94) |
| 78-84 | 2.11 (1.85, 2.40) | 2.83 (2.08, 3.86) | 1.98 (1.72, 2.29) | | 2.12 (1.74, 2.58) | 2.34 (1.57, 3.49) | 2.05 (1.63, 2.57) |
| 72-78 | 2.58 (2.26, 2.95) | 3.25 (2.44, 4.33) | 2.43 (2.09, 2.83) | | 1.72 (1.44, 2.06) | 1.71 (1.22, 2.39) | 1.72 (1.39, 2.13) |
| 66-72 | 2.79 (2.45, 3.18) | 3.51 (2.66, 4.62) | 2.59 (2.23, 3.02) | | 2.26 (1.87, 2.73) | 2.61 (1.78, 3.84) | 2.16 (1.74, 2.68) |
| 60-66 | 2.75 (2.43, 3.12) | 3.32 (2.49, 4.41) | 2.61 (2.27, 3.01) | | 2.21 (1.85, 2.66) | 2.24 (1.55, 3.25) | 2.20 (1.79, 2.72) |
| 54-60 | 2.45 (2.18, 2.76) | 3.03 (2.36, 3.90) | 2.30 (2.01, 2.63) | | 2.37 (1.98, 2.84) | 2.75 (1.88, 4.03) | 2.27 (1.85, 2.78) |
| 48-54 | 3.02 (2.69, 3.39) | 4.43 (3.39, 5.80) | 2.72 (2.39, 3.11) | | 2.38 (2.01, 2.83) | 2.17 (1.57, 3.01) | 2.47 (2.02, 3.02) |
| 42-48 | 3.21 (2.87, 3.60) | 5.11 (3.96, 6.58) | 2.82 (2.48, 3.20) | | 2.40 (2.02, 2.85) | 2.41 (1.66, 3.51) | 2.40 (1.97, 2.92) |
| 36-42 | 3.70 (3.31, 4.13) | 6.69 (5.16, 8.67) | 3.13 (2.76, 3.54) | | 2.08 (1.79, 2.42) | 2.15 (1.58, 2.92) | 2.06 (1.74, 2.45) |
| 30-36 | 4.06 (3.64, 4.52) | 6.16 (4.84, 7.84) | 3.58 (3.16, 4.05) | | 2.83 (2.41, 3.33) | 3.22 (2.27, 4.58) | 2.73 (2.28, 3.28) |
| 24-30 | 4.60 (4.14, 5.10) | 7.02 (5.55, 8.87) | 4.08 (3.63, 4.60) | | 2.88 (2.46, 3.38) | 2.49 (1.86, 3.34) | 3.06 (2.53, 3.69) |
| 18-24 | 5.17 (4.69, 5.70) | 7.53 (6.00, 9.44) | 4.65 (4.17, 5.20) | | 3.18 (2.71, 3.73) | 3.56 (2.53, 4.99) | 3.08 (2.57, 3.69) |
| 12-18 | 7.19 (6.54, 7.90) | 11.04 (8.95, 13.62) | 6.29 (5.65, 7.00) | | 2.63 (2.28, 3.04) | 2.61 (1.95, 3.48) | 2.64 (2.23, 3.11) |
| 6-12 | 11.05 (10.15, 12.04) | 15.33 (12.82, 18.32) | 9.91 (8.98, 10.93) | | 3.24 (2.82, 3.71) | 3.74 (2.80, 5.00) | 3.10 (2.65, 3.62) |
| 0-6 | 14.69 (13.51, 15.98) | 20.95 (17.58, 24.97) | 12.92 (11.72, 14.24) | | 3.69 (3.19, 4.27) | 4.41 (3.25, 5.98) | 3.49 (2.96, 4.12) |
|  | Sick leave | | | |  | | |
| 114-120 | 1.14 (1.07, 1.21) | 1.13 (1.02, 1.25) | 1.15 (1.07, 1.23) |  | |  |  |
| 108-114 | 1.19 (1.12, 1.27) | 1.19 (1.07, 1.32) | 1.20 (1.12, 1.29) |  | |  |  |
| 102-108 | 1.20 (1.14, 1.28) | 1.13 (1.02, 1.25) | 1.24 (1.16, 1.33) |  | |  |  |
| 96-102 | 1.23 (1.16, 1.31) | 1.23 (1.11, 1.37) | 1.23 (1.14, 1.32) |  | |  |  |
| 90-96 | 1.23 (1.15, 1.30) | 1.28 (1.15, 1.43) | 1.21 (1.12, 1.30) |  | |  |  |
| 84-90 | 1.30 (1.23, 1.38) | 1.21 (1.09, 1.35) | 1.35 (1.25, 1.45) |  | |  |  |
| 78-84 | 1.29 (1.22, 1.37) | 1.29 (1.16, 1.44) | 1.30 (1.21, 1.39) |  | |  |  |
| 72-78 | 1.23 (1.16, 1.31) | 1.13 (1.02, 1.26) | 1.29 (1.19, 1.38) |  | |  |  |
| 66-72 | 1.21 (1.14, 1.28) | 1.16 (1.04, 1.29) | 1.24 (1.15, 1.33) |  | |  |  |
| 60-66 | 1.22 (1.15, 1.30) | 1.15 (1.04, 1.28) | 1.25 (1.16, 1.35) |  | |  |  |
| 54-60 | 1.23 (1.15, 1.30) | 1.21 (1.09, 1.35) | 1.24 (1.16, 1.33) |  | |  |  |
| 48-54 | 1.32 (1.24, 1.40) | 1.33 (1.20, 1.49) | 1.32 (1.22, 1.42) |  | |  |  |
| 42-48 | 1.31 (1.23, 1.39) | 1.43 (1.28, 1.60) | 1.26 (1.17, 1.36) |  | |  |  |
| 36-42 | 1.42 (1.34, 1.52) | 1.52 (1.36, 1.70) | 1.39 (1.29, 1.50) |  | |  |  |
| 30-36 | 1.29 (1.21, 1.37) | 1.35 (1.21, 1.51) | 1.26 (1.17, 1.36) |  | |  |  |
| 24-30 | 1.52 (1.43, 1.62) | 1.48 (1.33, 1.66) | 1.54 (1.42, 1.66) |  | |  |  |
| 18-24 | 1.43 (1.34, 1.52) | 1.41 (1.27, 1.57) | 1.44 (1.33, 1.55) |  | |  |  |
| 12-18 | 1.67 (1.56, 1.78) | 1.68 (1.50, 1.88) | 1.66 (1.54, 1.80) |  | |  |  |
| 6-12 | 1.85 (1.73, 1.97) | 2.12 (1.90, 2.38) | 1.74 (1.61, 1.87) |  | |  |  |
| 0-6 | 2.47 (2.32, 2.62) | 3.10 (2.77, 3.47) | 2.21 (2.05, 2.39) |  | |  |  |

**Supplementary Table 4**

Prevalence rate ratio and 95% CIs of antidepressants and antiepileptics in each 6-month interval with most recent 6-month interval adjusted for sex, age, geographical region, ethnicity, neighbourhood deprivation, comorbidity, BMI, smoking status, drinking status, hip injury, depression/anxiety/stress and epilepsy.

| Months prior to index date | Antidepressants | | | | | |
| --- | --- | --- | --- | --- | --- | --- |
|  | Overall | | Male | | Female | |
|  | Cases; n=240,299 | Controls; n=240,299 | Cases; n=90,773 | Controls; n=90,773 | Cases; n=149,526 | Controls; n=149,526 |
| 114-120 | 0.63 (0.62, 0.64) | 0.84 (0.83, 0.86) | 0.54 (0.52, 0.56) | 0.82 (0.79, 0.86) | 0.66 (0.65, 0.67) | 0.85 (0.83, 0.87) |
| 108-114 | 0.66 (0.65, 0.67) | 0.88 (0.86, 0.89) | 0.57 (0.55, 0.59) | 0.85 (0.82, 0.89) | 0.69 (0.67, 0.70) | 0.88 (0.86, 0.90) |
| 102-108 | 0.67 (0.66, 0.68) | 0.89 (0.88, 0.91) | 0.58 (0.56, 0.60) | 0.89 (0.85, 0.93) | 0.70 (0.69, 0.72) | 0.90 (0.88, 0.92) |
| 96-102 | 0.69 (0.68, 0.70) | 0.91 (0.89, 0.93) | 0.60 (0.58, 0.62) | 0.89 (0.86, 0.93) | 0.72 (0.71, 0.73) | 0.91 (0.90, 0.93) |
| 90-96 | 0.71 (0.70, 0.72) | 0.93 (0.91, 0.94) | 0.62 (0.60, 0.64) | 0.92 (0.89, 0.96) | 0.74 (0.72, 0.75) | 0.93 (0.91, 0.95) |
| 84-90 | 0.72 (0.71, 0.74) | 0.94 (0.92, 0.95) | 0.64 (0.62, 0.67) | 0.93 (0.89, 0.97) | 0.75 (0.74, 0.76) | 0.94 (0.92, 0.96) |
| 78-84 | 0.74 (0.73, 0.75) | 0.96 (0.94, 0.97) | 0.66 (0.64, 0.68) | 0.96 (0.92, 1.00) | 0.76 (0.75, 0.78) | 0.96 (0.94, 0.98) |
| 72-78 | 0.75 (0.74, 0.76) | 0.96 (0.95, 0.98) | 0.67 (0.65, 0.69) | 0.96 (0.92, 1.00) | 0.78 (0.77, 0.79) | 0.96 (0.94, 0.98) |
| 66-72 | 0.77 (0.75, 0.78) | 0.97 (0.95, 0.99) | 0.69 (0.67, 0.71) | 0.97 (0.93, 1.01) | 0.79 (0.78, 0.80) | 0.97 (0.95, 0.99) |
| 60-66 | 0.78 (0.77, 0.79) | 0.99 (0.97, 1.00) | 0.71 (0.69, 0.74) | 1.00 (0.96, 1.04) | 0.80 (0.79, 0.81) | 0.98 (0.96, 1.00) |
| 54-60 | 0.80 (0.79, 0.81) | 0.98 (0.97, 1.00) | 0.73 (0.71, 0.76) | 1.00 (0.96, 1.04) | 0.82 (0.80, 0.83) | 0.98 (0.96, 1.00) |
| 48-54 | 0.81 (0.80, 0.83) | 0.99 (0.98, 1.01) | 0.75 (0.72, 0.77) | 1.01 (0.97, 1.05) | 0.83 (0.82, 0.85) | 0.99 (0.97, 1.01) |
| 42-48 | 0.83 (0.81, 0.84) | 1.01 (0.99, 1.03) | 0.77 (0.74, 0.79) | 1.02 (0.98, 1.07) | 0.84 (0.83, 0.86) | 1.00 (0.98, 1.02) |
| 36-42 | 0.84 (0.83, 0.85) | 1.01 (0.99, 1.02) | 0.78 (0.76, 0.81) | 1.01 (0.97, 1.05) | 0.86 (0.84, 0.87) | 1.00 (0.98, 1.02) |
| 30-36 | 0.85 (0.84, 0.86) | 1.01 (0.99, 1.03) | 0.79 (0.76, 0.81) | 1.01 (0.97, 1.05) | 0.87 (0.86, 0.89) | 1.01 (0.99, 1.03) |
| 24-30 | 0.88 (0.87, 0.89) | 1.02 (1.00, 1.03) | 0.83 (0.80, 0.85) | 1.01 (0.97, 1.05) | 0.90 (0.88, 0.91) | 1.02 (1.00, 1.04) |
| 18-24 | 0.90 (0.89, 0.91) | 1.01 (1.00, 1.03) | 0.85 (0.82, 0.87) | 1.01 (0.97, 1.05) | 0.92 (0.90, 0.93) | 1.01 (0.99, 1.04) |
| 12-18 | 0.93 (0.92, 0.95) | 1.00 (0.98, 1.02) | 0.89 (0.86, 0.92) | 1.00 (0.96, 1.04) | 0.95 (0.93, 0.96) | 1.00 (0.98, 1.02) |
| 6-12 | 0.98 (0.96, 0.99) | 1.00 (0.99, 1.02) | 0.96 (0.93, 0.99) | 1.01 (0.97, 1.05) | 0.98 (0.97, 1.00) | 1.00 (0.98, 1.02) |
| 0-6 | 1.00 (1.00, 1.00) | 1.00 (1.00, 1.00) | 1.00 (1.00, 1.00) | 1.00 (1.00, 1.00) | 1.00 (1.00, 1.00) | 1.00 (1.00, 1.00) |
|  | Antiepileptics | | | | | |
| 114-120 | 0.29 (0.28, 0.31) | 0.52 (0.50, 0.54) | 0.31 (0.29, 0.33) | 0.57 (0.53, 0.61) | 0.29 (0.28, 0.30) | 0.50 (0.47, 0.52) |
| 108-114 | 0.31 (0.30, 0.32) | 0.55 (0.53, 0.58) | 0.32 (0.30, 0.35) | 0.60 (0.56, 0.65) | 0.31 (0.29, 0.32) | 0.53 (0.51, 0.56) |
| 102-108 | 0.33 (0.32, 0.34) | 0.58 (0.56, 0.61) | 0.34 (0.32, 0.36) | 0.62 (0.58, 0.67) | 0.33 (0.31, 0.34) | 0.56 (0.53, 0.59) |
| 96-102 | 0.35 (0.34, 0.36) | 0.62 (0.59, 0.64) | 0.36 (0.34, 0.38) | 0.67 (0.62, 0.72) | 0.35 (0.33, 0.36) | 0.60 (0.57, 0.63) |
| 90-96 | 0.37 (0.35, 0.38) | 0.63 (0.60, 0.66) | 0.37 (0.35, 0.40) | 0.69 (0.64, 0.74) | 0.36 (0.35, 0.38) | 0.60 (0.57, 0.63) |
| 84-90 | 0.39 (0.37, 0.40) | 0.66 (0.63, 0.69) | 0.39 (0.37, 0.42) | 0.72 (0.67, 0.77) | 0.38 (0.37, 0.40) | 0.64 (0.61, 0.67) |
| 78-84 | 0.42 (0.40, 0.43) | 0.69 (0.66, 0.72) | 0.42 (0.40, 0.45) | 0.73 (0.68, 0.78) | 0.41 (0.40, 0.43) | 0.67 (0.64, 0.71) |
| 72-78 | 0.44 (0.43, 0.45) | 0.73 (0.70, 0.75) | 0.44 (0.42, 0.47) | 0.78 (0.73, 0.83) | 0.44 (0.42, 0.45) | 0.70 (0.67, 0.74) |
| 66-72 | 0.46 (0.45, 0.48) | 0.74 (0.72, 0.77) | 0.47 (0.44, 0.50) | 0.78 (0.73, 0.84) | 0.46 (0.44, 0.48) | 0.73 (0.69, 0.76) |
| 60-66 | 0.49 (0.48, 0.51) | 0.77 (0.74, 0.80) | 0.51 (0.48, 0.53) | 0.80 (0.74, 0.85) | 0.49 (0.47, 0.51) | 0.76 (0.72, 0.79) |
| 54-60 | 0.52 (0.51, 0.54) | 0.79 (0.76, 0.82) | 0.53 (0.50, 0.56) | 0.81 (0.76, 0.87) | 0.52 (0.50, 0.54) | 0.79 (0.75, 0.82) |
| 48-54 | 0.56 (0.54, 0.57) | 0.82 (0.79, 0.85) | 0.56 (0.53, 0.59) | 0.85 (0.79, 0.91) | 0.55 (0.53, 0.57) | 0.80 (0.77, 0.84) |
| 42-48 | 0.59 (0.57, 0.61) | 0.84 (0.81, 0.87) | 0.59 (0.56, 0.62) | 0.88 (0.82, 0.94) | 0.59 (0.57, 0.61) | 0.82 (0.79, 0.86) |
| 36-42 | 0.62 (0.60, 0.64) | 0.87 (0.84, 0.90) | 0.61 (0.58, 0.64) | 0.91 (0.85, 0.97) | 0.62 (0.60, 0.65) | 0.85 (0.82, 0.89) |
| 30-36 | 0.66 (0.65, 0.68) | 0.88 (0.85, 0.91) | 0.65 (0.62, 0.69) | 0.89 (0.84, 0.95) | 0.67 (0.65, 0.69) | 0.88 (0.84, 0.92) |
| 24-30 | 0.71 (0.69, 0.73) | 0.92 (0.88, 0.95) | 0.70 (0.67, 0.74) | 0.94 (0.88, 1.00) | 0.71 (0.69, 0.74) | 0.91 (0.87, 0.95) |
| 18-24 | 0.76 (0.74, 0.78) | 0.93 (0.90, 0.97) | 0.74 (0.71, 0.78) | 0.94 (0.88, 1.01) | 0.77 (0.74, 0.79) | 0.93 (0.89, 0.97) |
| 12-18 | 0.83 (0.81, 0.85) | 0.96 (0.92, 0.99) | 0.81 (0.77, 0.85) | 0.97 (0.91, 1.04) | 0.84 (0.81, 0.86) | 0.95 (0.91, 0.99) |
| 6-12 | 0.93 (0.91, 0.96) | 1.00 (0.96, 1.03) | 0.92 (0.87, 0.96) | 0.99 (0.93, 1.06) | 0.94 (0.91, 0.97) | 1.00 (0.96, 1.04) |
| 0-6 | 1.00 (1.00, 1.00) | 1.00 (1.00, 1.00) | 1.00 (1.00, 1.00) | 1.00 (1.00, 1.00) | 1.00 (1.00, 1.00) | 1.00 (1.00, 1.00) |

**Supplementary Table 5**

Conditional odds ratios and 95% CIs for primary THR associated with antidepressants and antiepileptics within each 6-month period adjusted for geographical region, ethnicity, neighbourhood deprivation, comorbidity, BMI, smoking status, drinking status, hip injury, depression/anxiety/stress and epilepsy.

| Months prior to index date | Antidepressants | | | Antiepileptics | | |
| --- | --- | --- | --- | --- | --- | --- |
|  | Overall | Male | Female | Overall | Male | Female |
|  | OR (95% CI) | OR (95% CI) | OR (95% CI) | OR (95% CI) | OR (95% CI) | OR (95% CI) |
| 114-120 | 1.10 (1.06, 1.13) | 0.96 (0.90, 1.03) | 1.14 (1.10, 1.18) | 1.05 (0.98, 1.13) | 0.94 (0.83, 1.06) | 1.10 (1.01, 1.20) |
| 108-114 | 1.11 (1.07, 1.14) | 0.98 (0.92, 1.05) | 1.14 (1.11, 1.19) | 1.05 (0.99, 1.13) | 0.95 (0.84, 1.07) | 1.11 (1.02, 1.20) |
| 102-108 | 1.12 (1.08, 1.15) | 0.98 (0.92, 1.05) | 1.16 (1.12, 1.20) | 1.08 (1.02, 1.16) | 0.96 (0.85, 1.08) | 1.14 (1.06, 1.24) |
| 96-102 | 1.12 (1.09, 1.15) | 1.02 (0.95, 1.09) | 1.15 (1.11, 1.19) | 1.05 (0.98, 1.12) | 0.94 (0.84, 1.06) | 1.10 (1.02, 1.19) |
| 90-96 | 1.14 (1.11, 1.18) | 1.03 (0.96, 1.10) | 1.18 (1.14, 1.22) | 1.05 (0.99, 1.12) | 0.93 (0.83, 1.04) | 1.11 (1.03, 1.20) |
| 84-90 | 1.15 (1.11, 1.18) | 1.05 (0.98, 1.12) | 1.17 (1.13, 1.21) | 1.08 (1.02, 1.15) | 0.94 (0.85, 1.05) | 1.14 (1.06, 1.23) |
| 78-84 | 1.16 (1.13, 1.20) | 1.06 (0.99, 1.13) | 1.19 (1.15, 1.23) | 1.13 (1.07, 1.20) | 1.03 (0.93, 1.15) | 1.18 (1.10, 1.27) |
| 72-78 | 1.17 (1.14, 1.21) | 1.06 (0.99, 1.13) | 1.20 (1.16, 1.25) | 1.16 (1.09, 1.23) | 1.05 (0.95, 1.17) | 1.21 (1.13, 1.30) |
| 66-72 | 1.18 (1.15, 1.22) | 1.07 (1.00, 1.14) | 1.22 (1.18, 1.26) | 1.18 (1.12, 1.25) | 1.09 (0.98, 1.21) | 1.22 (1.14, 1.31) |
| 60-66 | 1.19 (1.16, 1.23) | 1.09 (1.02, 1.16) | 1.22 (1.18, 1.26) | 1.24 (1.18, 1.32) | 1.19 (1.08, 1.32) | 1.26 (1.18, 1.35) |
| 54-60 | 1.22 (1.19, 1.26) | 1.11 (1.05, 1.19) | 1.26 (1.21, 1.30) | 1.27 (1.21, 1.35) | 1.25 (1.13, 1.38) | 1.28 (1.20, 1.37) |
| 48-54 | 1.24 (1.20, 1.28) | 1.11 (1.04, 1.18) | 1.28 (1.24, 1.32) | 1.29 (1.23, 1.37) | 1.21 (1.10, 1.34) | 1.33 (1.25, 1.42) |
| 42-48 | 1.24 (1.20, 1.28) | 1.14 (1.07, 1.21) | 1.27 (1.23, 1.31) | 1.37 (1.30, 1.45) | 1.26 (1.14, 1.39) | 1.42 (1.33, 1.51) |
| 36-42 | 1.28 (1.24, 1.31) | 1.19 (1.12, 1.27) | 1.30 (1.26, 1.35) | 1.38 (1.31, 1.45) | 1.24 (1.13, 1.37) | 1.45 (1.36, 1.54) |
| 30-36 | 1.30 (1.26, 1.34) | 1.22 (1.15, 1.30) | 1.32 (1.28, 1.37) | 1.47 (1.39, 1.54) | 1.42 (1.29, 1.56) | 1.49 (1.40, 1.58) |
| 24-30 | 1.34 (1.30, 1.37) | 1.26 (1.19, 1.34) | 1.36 (1.32, 1.40) | 1.50 (1.42, 1.57) | 1.41 (1.29, 1.54) | 1.54 (1.45, 1.63) |
| 18-24 | 1.39 (1.35, 1.43) | 1.30 (1.22, 1.38) | 1.41 (1.37, 1.46) | 1.60 (1.52, 1.68) | 1.53 (1.40, 1.67) | 1.63 (1.54, 1.73) |
| 12-18 | 1.48 (1.44, 1.52) | 1.40 (1.32, 1.49) | 1.50 (1.45, 1.55) | 1.68 (1.61, 1.77) | 1.62 (1.48, 1.77) | 1.71 (1.62, 1.81) |
| 6-12 | 1.58 (1.54, 1.63) | 1.51 (1.43, 1.61) | 1.60 (1.55, 1.66) | 1.84 (1.76, 1.92) | 1.80 (1.65, 1.96) | 1.86 (1.76, 1.96) |
| 0-6 | 1.62 (1.57, 1.66) | 1.60 (1.51, 1.70) | 1.62 (1.57, 1.68) | 1.95 (1.87, 2.04) | 1.89 (1.74, 2.06) | 1.98 (1.88, 2.09) |

**Supplementary Table 6**

Prevalence and 95% CIs of sick leave within 10 years prior to index date in case and control groups, overall and by gender stratified by age.

| Age group :30-44 | | | | | | |
| --- | --- | --- | --- | --- | --- | --- |
| Months prior to index date | Overall | | Male | | Female | |
|  | Cases;  n=13061 | Controls; n=13061 | Cases;  n=5312 | Controls; n=5312 | Cases;  n=7749 | Controls; n=7749 |
| 114-120 | 2.97 (2.68, 3.26) | 1.61 (1.39, 1.82) | 2.52 (2.10, 2.94) | 1.49 (1.16, 1.81) | 3.28 (2.88, 3.67) | 1.69 (1.40, 1.98) |
| 108-114 | 2.91 (2.62, 3.20) | 1.91 (1.67, 2.14) | 2.41 (2.00, 2.82) | 1.83 (1.47, 2.19) | 3.25 (2.86, 3.65) | 1.96 (1.65, 2.27) |
| 102-108 | 3.02 (2.73, 3.32) | 1.97 (1.73, 2.21) | 2.41 (2.00, 2.82) | 1.69 (1.35, 2.04) | 3.45 (3.04, 3.85) | 2.16 (1.83, 2.48) |
| 96-102 | 2.74 (2.46, 3.02) | 1.90 (1.66, 2.13) | 2.30 (1.89, 2.70) | 1.56 (1.23, 1.90) | 3.05 (2.66, 3.43) | 2.13 (1.81, 2.45) |
| 90-96 | 3.09 (2.80, 3.39) | 1.83 (1.60, 2.06) | 2.43 (2.01, 2.84) | 1.52 (1.20, 1.85) | 3.55 (3.14, 3.96) | 2.04 (1.72, 2.35) |
| 84-90 | 2.87 (2.58, 3.16) | 2.07 (1.82, 2.31) | 2.18 (1.79, 2.58) | 1.71 (1.36, 2.06) | 3.34 (2.94, 3.74) | 2.31 (1.98, 2.64) |
| 78-84 | 2.98 (2.69, 3.27) | 1.87 (1.64, 2.10) | 2.52 (2.10, 2.94) | 1.32 (1.01, 1.62) | 3.29 (2.89, 3.69) | 2.25 (1.92, 2.58) |
| 72-78 | 3.22 (2.91, 3.52) | 2.02 (1.78, 2.26) | 2.48 (2.07, 2.90) | 1.75 (1.40, 2.10) | 3.72 (3.30, 4.14) | 2.21 (1.88, 2.53) |
| 66-72 | 3.14 (2.84, 3.44) | 2.01 (1.77, 2.25) | 2.45 (2.03, 2.86) | 1.79 (1.43, 2.14) | 3.61 (3.20, 4.03) | 2.17 (1.84, 2.49) |
| 60-66 | 2.96 (2.67, 3.25) | 1.94 (1.70, 2.17) | 2.65 (2.22, 3.09) | 1.84 (1.48, 2.21) | 3.17 (2.78, 3.56) | 2.00 (1.69, 2.31) |
| 54-60 | 3.08 (2.78, 3.37) | 2.12 (1.87, 2.37) | 2.43 (2.01, 2.84) | 1.83 (1.47, 2.19) | 3.52 (3.11, 3.93) | 2.32 (1.99, 2.66) |
| 48-54 | 3.25 (2.94, 3.55) | 2.04 (1.80, 2.29) | 2.65 (2.22, 3.09) | 1.60 (1.26, 1.94) | 3.65 (3.23, 4.07) | 2.35 (2.01, 2.69) |
| 42-48 | 3.32 (3.02, 3.63) | 2.01 (1.77, 2.25) | 2.75 (2.31, 3.19) | 1.54 (1.21, 1.88) | 3.72 (3.30, 4.14) | 2.34 (2.00, 2.67) |
| 36-42 | 3.48 (3.16, 3.79) | 2.14 (1.90, 2.39) | 2.90 (2.45, 3.35) | 1.52 (1.20, 1.85) | 3.87 (3.44, 4.30) | 2.57 (2.22, 2.92) |
| 30-36 | 3.44 (3.13, 3.75) | 2.24 (1.99, 2.50) | 3.14 (2.67, 3.61) | 1.68 (1.33, 2.02) | 3.64 (3.22, 4.06) | 2.63 (2.28, 2.99) |
| 24-30 | 3.44 (3.13, 3.75) | 2.32 (2.06, 2.58) | 2.50 (2.08, 2.92) | 1.81 (1.45, 2.17) | 4.08 (3.64, 4.52) | 2.67 (2.31, 3.03) |
| 18-24 | 3.94 (3.61, 4.28) | 2.28 (2.03, 2.54) | 3.77 (3.25, 4.28) | 1.81 (1.45, 2.17) | 4.07 (3.63, 4.50) | 2.61 (2.25, 2.96) |
| 12-18 | 4.04 (3.70, 4.38) | 2.12 (1.87, 2.37) | 3.65 (3.15, 4.16) | 1.75 (1.40, 2.10) | 4.31 (3.86, 4.76) | 2.37 (2.04, 2.71) |
| 6-12 | 4.59 (4.23, 4.95) | 2.12 (1.87, 2.37) | 4.03 (3.50, 4.56) | 1.84 (1.48, 2.21) | 4.98 (4.50, 5.47) | 2.31 (1.98, 2.64) |
| 0-6 | 5.61 (5.22, 6.01) | 2.21 (1.96, 2.46) | 5.03 (4.44, 5.61) | 1.77 (1.42, 2.12) | 6.01 (5.48, 6.54) | 2.52 (2.17, 2.87) |
| Age group :45-54 | | | | | | |
| Months prior to index date | Overall | | Male | | Female | |
|  | Cases;  n=27670 | Controls; n=27670 | Cases;  n=11628 | Controls; n=11628 | Cases;  n=16042 | Controls; n=16042 |
| 114-120 | 3.71 (3.49, 3.93) | 2.53 (2.34, 2.71) | 2.97 (2.66, 3.28) | 2.03 (1.77, 2.29) | 4.25 (3.93, 4.56) | 2.89 (2.63, 3.15) |
| 108-114 | 3.56 (3.34, 3.78) | 2.56 (2.37, 2.74) | 2.75 (2.45, 3.05) | 2.11 (1.85, 2.37) | 4.15 (3.84, 4.45) | 2.88 (2.62, 3.14) |
| 102-108 | 4.05 (3.82, 4.28) | 2.83 (2.63, 3.02) | 3.16 (2.84, 3.47) | 2.27 (2.00, 2.54) | 4.69 (4.37, 5.02) | 3.23 (2.96, 3.50) |
| 96-102 | 4.05 (3.82, 4.29) | 2.74 (2.54, 2.93) | 3.08 (2.76, 3.39) | 2.16 (1.89, 2.42) | 4.76 (4.43, 5.09) | 3.15 (2.88, 3.42) |
| 90-96 | 3.96 (3.73, 4.19) | 2.70 (2.51, 2.89) | 2.98 (2.67, 3.29) | 1.98 (1.72, 2.23) | 4.66 (4.34, 4.99) | 3.22 (2.95, 3.50) |
| 84-90 | 3.98 (3.75, 4.21) | 2.76 (2.56, 2.95) | 2.90 (2.59, 3.20) | 2.08 (1.82, 2.34) | 4.77 (4.44, 5.10) | 3.25 (2.97, 3.52) |
| 78-84 | 4.12 (3.88, 4.35) | 2.95 (2.75, 3.15) | 3.01 (2.70, 3.32) | 2.11 (1.85, 2.37) | 4.92 (4.58, 5.25) | 3.56 (3.27, 3.85) |
| 72-78 | 4.21 (3.98, 4.45) | 2.95 (2.75, 3.14) | 2.94 (2.63, 3.25) | 2.27 (2.00, 2.54) | 5.14 (4.79, 5.48) | 3.43 (3.15, 3.72) |
| 66-72 | 4.39 (4.15, 4.64) | 3.26 (3.05, 3.47) | 3.03 (2.72, 3.34) | 2.33 (2.06, 2.60) | 5.39 (5.04, 5.74) | 3.94 (3.64, 4.24) |
| 60-66 | 4.64 (4.39, 4.88) | 3.34 (3.13, 3.55) | 3.47 (3.14, 3.81) | 2.46 (2.18, 2.74) | 5.48 (5.13, 5.83) | 3.98 (3.68, 4.29) |
| 54-60 | 4.62 (4.37, 4.86) | 3.41 (3.19, 3.62) | 3.38 (3.05, 3.71) | 2.31 (2.04, 2.59) | 5.51 (5.16, 5.86) | 4.20 (3.89, 4.51) |
| 48-54 | 4.93 (4.68, 5.19) | 3.37 (3.16, 3.58) | 3.66 (3.32, 4.01) | 2.39 (2.11, 2.67) | 5.85 (5.49, 6.22) | 4.08 (3.78, 4.39) |
| 42-48 | 4.91 (4.65, 5.16) | 3.33 (3.12, 3.54) | 3.48 (3.15, 3.82) | 2.30 (2.02, 2.57) | 5.94 (5.57, 6.31) | 4.08 (3.78, 4.39) |
| 36-42 | 5.08 (4.83, 5.34) | 3.18 (2.97, 3.39) | 3.54 (3.21, 3.88) | 2.32 (2.05, 2.60) | 6.20 (5.83, 6.58) | 3.80 (3.51, 4.10) |
| 30-36 | 5.16 (4.90, 5.42) | 3.32 (3.11, 3.53) | 3.89 (3.54, 4.24) | 2.38 (2.11, 2.66) | 6.08 (5.71, 6.45) | 4.00 (3.70, 4.31) |
| 24-30 | 5.54 (5.27, 5.81) | 3.34 (3.12, 3.55) | 4.21 (3.84, 4.57) | 2.24 (1.97, 2.50) | 6.51 (6.13, 6.90) | 4.13 (3.82, 4.44) |
| 18-24 | 5.74 (5.47, 6.02) | 3.58 (3.36, 3.80) | 4.28 (3.91, 4.65) | 2.66 (2.37, 2.95) | 6.80 (6.41, 7.19) | 4.25 (3.93, 4.56) |
| 12-18 | 6.15 (5.86, 6.43) | 3.44 (3.23, 3.66) | 4.80 (4.41, 5.19) | 2.28 (2.01, 2.55) | 7.13 (6.73, 7.52) | 4.29 (3.98, 4.60) |
| 6-12 | 6.95 (6.65, 7.25) | 3.45 (3.23, 3.66) | 5.37 (4.96, 5.78) | 2.43 (2.15, 2.71) | 8.10 (7.68, 8.53) | 4.18 (3.87, 4.49) |
| 0-6 | 8.40 (8.08, 8.73) | 3.52 (3.30, 3.74) | 7.19 (6.72, 7.66) | 2.43 (2.15, 2.70) | 9.28 (8.83, 9.73) | 4.31 (4.00, 4.63) |
| Age group :55-64 | | | | | | |
| Months prior to index date | Overall | | Male | | Female | |
|  | Cases;  n=51323 | Controls; n=51323 | Cases;  n=21410 | Controls; n=21410 | Cases;  n=29913 | Controls; n=29913 |
| 114-120 | 4.12 (3.95, 4.30) | 3.45 (3.29, 3.61) | 3.18 (2.94, 3.41) | 2.57 (2.36, 2.78) | 4.80 (4.56, 5.05) | 4.08 (3.86, 4.31) |
| 108-114 | 4.30 (4.12, 4.47) | 3.42 (3.26, 3.57) | 3.39 (3.14, 3.63) | 2.77 (2.55, 2.99) | 4.95 (4.70, 5.19) | 3.88 (3.66, 4.10) |
| 102-108 | 4.37 (4.20, 4.55) | 3.52 (3.36, 3.68) | 3.34 (3.10, 3.58) | 2.65 (2.44, 2.87) | 5.11 (4.86, 5.36) | 4.14 (3.91, 4.36) |
| 96-102 | 4.34 (4.17, 4.52) | 3.52 (3.36, 3.68) | 3.52 (3.27, 3.76) | 2.79 (2.57, 3.01) | 4.93 (4.69, 5.18) | 4.05 (3.82, 4.27) |
| 90-96 | 4.61 (4.43, 4.79) | 3.72 (3.55, 3.88) | 3.85 (3.59, 4.11) | 3.00 (2.77, 3.23) | 5.15 (4.90, 5.40) | 4.23 (4.00, 4.46) |
| 84-90 | 4.71 (4.53, 4.89) | 3.67 (3.51, 3.84) | 3.71 (3.46, 3.97) | 3.14 (2.91, 3.38) | 5.43 (5.17, 5.68) | 4.06 (3.83, 4.28) |
| 78-84 | 4.72 (4.53, 4.90) | 3.86 (3.69, 4.03) | 3.79 (3.53, 4.04) | 2.89 (2.67, 3.12) | 5.38 (5.13, 5.64) | 4.55 (4.32, 4.79) |
| 72-78 | 4.77 (4.58, 4.95) | 3.78 (3.62, 3.95) | 3.78 (3.52, 4.03) | 2.88 (2.66, 3.11) | 5.48 (5.22, 5.73) | 4.43 (4.20, 4.66) |
| 66-72 | 5.10 (4.91, 5.29) | 4.05 (3.88, 4.22) | 4.31 (4.04, 4.58) | 3.17 (2.94, 3.41) | 5.67 (5.41, 5.93) | 4.68 (4.44, 4.92) |
| 60-66 | 4.94 (4.76, 5.13) | 3.86 (3.69, 4.02) | 4.11 (3.84, 4.37) | 3.08 (2.85, 3.31) | 5.54 (5.28, 5.80) | 4.41 (4.18, 4.65) |
| 54-60 | 5.32 (5.12, 5.51) | 4.12 (3.95, 4.30) | 4.31 (4.04, 4.58) | 3.32 (3.08, 3.56) | 6.04 (5.77, 6.31) | 4.70 (4.46, 4.94) |
| 48-54 | 5.27 (5.08, 5.46) | 3.82 (3.65, 3.98) | 4.26 (3.99, 4.54) | 3.07 (2.84, 3.30) | 5.99 (5.72, 6.26) | 4.35 (4.11, 4.58) |
| 42-48 | 5.32 (5.12, 5.51) | 4.11 (3.94, 4.28) | 4.41 (4.14, 4.69) | 3.20 (2.97, 3.44) | 5.96 (5.70, 6.23) | 4.76 (4.52, 5.00) |
| 36-42 | 5.33 (5.14, 5.53) | 3.90 (3.73, 4.06) | 4.48 (4.20, 4.76) | 3.21 (2.97, 3.44) | 5.94 (5.67, 6.21) | 4.39 (4.16, 4.62) |
| 30-36 | 5.51 (5.31, 5.71) | 4.15 (3.98, 4.32) | 4.68 (4.40, 4.96) | 3.33 (3.09, 3.57) | 6.11 (5.84, 6.38) | 4.74 (4.50, 4.98) |
| 24-30 | 5.61 (5.41, 5.81) | 3.70 (3.54, 3.87) | 5.10 (4.80, 5.39) | 3.13 (2.90, 3.37) | 5.98 (5.71, 6.25) | 4.11 (3.89, 4.34) |
| 18-24 | 5.76 (5.56, 5.97) | 4.07 (3.90, 4.24) | 5.17 (4.87, 5.46) | 3.50 (3.25, 3.74) | 6.19 (5.92, 6.46) | 4.47 (4.24, 4.71) |
| 12-18 | 6.05 (5.84, 6.25) | 3.64 (3.48, 3.81) | 5.65 (5.34, 5.96) | 3.30 (3.06, 3.54) | 6.33 (6.06, 6.61) | 3.89 (3.67, 4.11) |
| 6-12 | 6.71 (6.49, 6.92) | 3.78 (3.62, 3.95) | 6.63 (6.29, 6.96) | 3.45 (3.20, 3.69) | 6.76 (6.48, 7.05) | 4.03 (3.80, 4.25) |
| 0-6 | 8.58 (8.34, 8.83) | 3.50 (3.34, 3.66) | 9.01 (8.62, 9.39) | 3.19 (2.96, 3.43) | 8.28 (7.97, 8.60) | 3.72 (3.51, 3.94) |
| Age group :65-74 | | | | | | |
| Months prior to index date | Overall | | Male | | Female | |
|  | Cases;  n=75544 | Controls; n=75544 | Cases;  n=28509 | Controls; n=28509 | Cases;  n=47035 | Controls; n=47035 |
| 114-120 | 2.81 (2.69, 2.93) | 2.53 (2.42, 2.64) | 2.96 (2.76, 3.16) | 2.60 (2.41, 2.78) | 2.72 (2.57, 2.87) | 2.49 (2.34, 2.63) |
| 108-114 | 2.83 (2.71, 2.95) | 2.43 (2.32, 2.53) | 3.05 (2.85, 3.25) | 2.60 (2.42, 2.79) | 2.70 (2.55, 2.84) | 2.32 (2.18, 2.45) |
| 102-108 | 2.81 (2.69, 2.93) | 2.27 (2.16, 2.37) | 3.08 (2.88, 3.28) | 2.44 (2.27, 2.62) | 2.64 (2.50, 2.79) | 2.16 (2.03, 2.29) |
| 96-102 | 2.49 (2.38, 2.60) | 2.09 (1.99, 2.19) | 2.72 (2.53, 2.91) | 2.34 (2.16, 2.52) | 2.35 (2.21, 2.48) | 1.94 (1.81, 2.06) |
| 90-96 | 2.46 (2.35, 2.57) | 2.06 (1.96, 2.16) | 2.73 (2.54, 2.91) | 2.27 (2.09, 2.44) | 2.30 (2.17, 2.44) | 1.94 (1.81, 2.06) |
| 84-90 | 2.43 (2.32, 2.54) | 1.91 (1.81, 2.01) | 2.73 (2.54, 2.92) | 2.04 (1.88, 2.21) | 2.25 (2.11, 2.38) | 1.83 (1.71, 1.95) |
| 78-84 | 2.28 (2.18, 2.39) | 1.76 (1.66, 1.85) | 2.60 (2.42, 2.79) | 1.99 (1.83, 2.15) | 2.09 (1.96, 2.22) | 1.62 (1.50, 1.73) |
| 72-78 | 2.07 (1.97, 2.18) | 1.61 (1.52, 1.70) | 2.31 (2.13, 2.48) | 1.88 (1.72, 2.04) | 1.93 (1.81, 2.06) | 1.45 (1.34, 1.55) |
| 66-72 | 2.04 (1.94, 2.14) | 1.63 (1.54, 1.72) | 2.34 (2.17, 2.52) | 1.92 (1.76, 2.08) | 1.85 (1.73, 1.97) | 1.45 (1.34, 1.56) |
| 60-66 | 1.92 (1.82, 2.02) | 1.44 (1.36, 1.53) | 2.27 (2.10, 2.44) | 1.69 (1.54, 1.84) | 1.71 (1.59, 1.83) | 1.29 (1.19, 1.39) |
| 54-60 | 1.83 (1.74, 1.93) | 1.36 (1.28, 1.45) | 2.10 (1.93, 2.26) | 1.60 (1.46, 1.75) | 1.68 (1.56, 1.79) | 1.22 (1.12, 1.32) |
| 48-54 | 1.77 (1.68, 1.86) | 1.23 (1.15, 1.31) | 2.03 (1.87, 2.20) | 1.41 (1.28, 1.55) | 1.61 (1.50, 1.72) | 1.12 (1.02, 1.21) |
| 42-48 | 1.55 (1.46, 1.64) | 1.09 (1.01, 1.16) | 1.84 (1.69, 2.00) | 1.16 (1.03, 1.28) | 1.37 (1.26, 1.47) | 1.04 (0.95, 1.13) |
| 36-42 | 1.46 (1.37, 1.54) | 0.93 (0.87, 1.00) | 1.80 (1.64, 1.95) | 0.99 (0.87, 1.10) | 1.25 (1.15, 1.35) | 0.90 (0.82, 0.99) |
| 30-36 | 1.33 (1.25, 1.41) | 0.95 (0.88, 1.02) | 1.66 (1.51, 1.81) | 1.05 (0.93, 1.17) | 1.13 (1.04, 1.23) | 0.89 (0.81, 0.98) |
| 24-30 | 1.18 (1.10, 1.26) | 0.73 (0.67, 0.79) | 1.40 (1.26, 1.53) | 0.84 (0.73, 0.94) | 1.05 (0.96, 1.14) | 0.66 (0.59, 0.74) |
| 18-24 | 1.11 (1.03, 1.18) | 0.72 (0.66, 0.78) | 1.27 (1.14, 1.40) | 0.79 (0.69, 0.90) | 1.01 (0.92, 1.10) | 0.67 (0.60, 0.74) |
| 12-18 | 1.07 (1.00, 1.14) | 0.61 (0.56, 0.67) | 1.18 (1.05, 1.30) | 0.60 (0.51, 0.69) | 1.01 (0.92, 1.10) | 0.62 (0.55, 0.69) |
| 6-12 | 1.10 (1.03, 1.18) | 0.60 (0.54, 0.65) | 1.26 (1.13, 1.39) | 0.56 (0.48, 0.65) | 1.01 (0.92, 1.10) | 0.62 (0.55, 0.69) |
| 0-6 | 1.20 (1.12, 1.28) | 0.44 (0.40, 0.49) | 1.44 (1.30, 1.58) | 0.41 (0.34, 0.49) | 1.05 (0.96, 1.14) | 0.46 (0.40, 0.52) |
| Age group :75-84 | | | | | | |
| Months prior to index date | Overall | | Male | | Female | |
|  | Cases;  n=55650 | Controls; n=55650 | Cases;  n=19114 | Controls; n=19114 | Cases;  n=36536 | Controls; n=36536 |
| 114-120 | 0.31 (0.26, 0.36) | 0.22 (0.18, 0.26) | 0.34 (0.26, 0.42) | 0.20 (0.14, 0.27) | 0.29 (0.24, 0.35) | 0.23 (0.18, 0.28) |
| 108-114 | 0.24 (0.20, 0.29) | 0.20 (0.16, 0.24) | 0.31 (0.23, 0.39) | 0.20 (0.14, 0.26) | 0.21 (0.16, 0.26) | 0.20 (0.16, 0.25) |
| 102-108 | 0.26 (0.22, 0.30) | 0.19 (0.16, 0.23) | 0.24 (0.17, 0.31) | 0.19 (0.13, 0.25) | 0.27 (0.22, 0.32) | 0.20 (0.15, 0.24) |
| 96-102 | 0.19 (0.15, 0.22) | 0.15 (0.12, 0.19) | 0.21 (0.14, 0.27) | 0.15 (0.09, 0.20) | 0.18 (0.13, 0.22) | 0.16 (0.12, 0.20) |
| 90-96 | 0.18 (0.15, 0.22) | 0.19 (0.15, 0.22) | 0.17 (0.11, 0.23) | 0.17 (0.11, 0.23) | 0.19 (0.15, 0.24) | 0.19 (0.15, 0.24) |
| 84-90 | 0.19 (0.15, 0.23) | 0.15 (0.12, 0.19) | 0.18 (0.12, 0.24) | 0.16 (0.11, 0.22) | 0.20 (0.15, 0.24) | 0.15 (0.11, 0.19) |
| 78-84 | 0.17 (0.13, 0.20) | 0.14 (0.11, 0.17) | 0.14 (0.09, 0.19) | 0.10 (0.05, 0.14) | 0.18 (0.13, 0.22) | 0.16 (0.12, 0.20) |
| 72-78 | 0.16 (0.13, 0.19) | 0.12 (0.09, 0.15) | 0.13 (0.08, 0.18) | 0.08 (0.04, 0.12) | 0.18 (0.13, 0.22) | 0.14 (0.10, 0.17) |
| 66-72 | 0.19 (0.16, 0.23) | 0.13 (0.10, 0.16) | 0.15 (0.09, 0.20) | 0.13 (0.08, 0.18) | 0.22 (0.17, 0.27) | 0.13 (0.10, 0.17) |
| 60-66 | 0.15 (0.12, 0.19) | 0.10 (0.08, 0.13) | 0.14 (0.08, 0.19) | 0.08 (0.04, 0.12) | 0.16 (0.12, 0.21) | 0.11 (0.08, 0.15) |
| 54-60 | 0.16 (0.13, 0.19) | 0.12 (0.09, 0.15) | 0.10 (0.05, 0.14) | 0.13 (0.08, 0.18) | 0.19 (0.15, 0.24) | 0.12 (0.08, 0.16) |
| 48-54 | 0.14 (0.11, 0.18) | 0.09 (0.06, 0.11) | 0.13 (0.08, 0.18) | 0.10 (0.06, 0.15) | 0.15 (0.11, 0.19) | 0.08 (0.05, 0.11) |
| 42-48 | 0.15 (0.12, 0.18) | 0.12 (0.09, 0.14) | 0.10 (0.05, 0.14) | 0.13 (0.08, 0.18) | 0.17 (0.13, 0.21) | 0.11 (0.08, 0.14) |
| 36-42 | 0.12 (0.09, 0.15) | 0.08 (0.06, 0.11) | 0.09 (0.05, 0.14) | 0.09 (0.05, 0.14) | 0.13 (0.10, 0.17) | 0.08 (0.05, 0.11) |
| 30-36 | 0.11 (0.08, 0.14) | 0.09 (0.07, 0.12) | 0.12 (0.07, 0.16) | 0.06 (0.02, 0.09) | 0.11 (0.08, 0.14) | 0.11 (0.08, 0.14) |
| 24-30 | 0.11 (0.08, 0.14) | 0.06 (0.04, 0.08) | 0.07 (0.03, 0.11) | 0.06 (0.02, 0.09) | 0.13 (0.09, 0.17) | 0.06 (0.04, 0.09) |
| 18-24 | 0.12 (0.09, 0.15) | 0.08 (0.06, 0.10) | 0.12 (0.07, 0.16) | 0.06 (0.02, 0.09) | 0.13 (0.09, 0.17) | 0.09 (0.06, 0.12) |
| 12-18 | 0.14 (0.11, 0.17) | 0.07 (0.05, 0.10) | 0.14 (0.09, 0.19) | 0.10 (0.05, 0.14) | 0.13 (0.10, 0.17) | 0.06 (0.04, 0.09) |
| 6-12 | 0.14 (0.11, 0.17) | 0.05 (0.03, 0.07) | 0.14 (0.08, 0.19) | 0.04 (0.01, 0.06) | 0.15 (0.11, 0.18) | 0.06 (0.03, 0.08) |
| 0-6 | 0.14 (0.11, 0.18) | 0.05 (0.03, 0.07) | 0.15 (0.10, 0.21) | 0.04 (0.01, 0.06) | 0.14 (0.10, 0.18) | 0.06 (0.04, 0.09) |

**Supplementary Table 7**

Prevalence rate ratio and 95% CIs of sick leave stratified by age in each 6-month interval with most recent 6-month interval adjusted for sex, age, geographical region, ethnicity, neighbourhood deprivation, comorbidity, BMI, smoking status, drinking status, and hip injury.

| Months prior to index date | Age group: 30-44 | | Age group: 45-54 | | Age group: 55-64 | |
| --- | --- | --- | --- | --- | --- | --- |
|  | Cases;  n=13061 | Controls; n=13061 | Cases;  n=27670 | Controls; n=27670 | Cases;  n=51323 | Controls; n=51323 |
| 114-120 | 0.55 (0.43, 0.70) | 0.70 (0.47, 1.06) | 0.62 (0.55, 0.71) | 0.78 (0.64, 0.95) | 0.46 (0.44, 0.49) | 0.92 (0.86, 0.99) |
| 108-114 | 0.67 (0.53, 0.85) | 0.93 (0.64, 1.35) | 0.62 (0.54, 0.70) | 0.79 (0.65, 0.96) | 0.49 (0.46, 0.51) | 0.93 (0.86, 0.99) |
| 102-108 | 0.80 (0.65, 1.00) | 0.76 (0.51, 1.13) | 0.70 (0.62, 0.78) | 0.89 (0.74, 1.08) | 0.51 (0.48, 0.53) | 0.96 (0.90, 1.03) |
| 96-102 | 0.77 (0.62, 0.95) | 0.87 (0.59, 1.27) | 0.69 (0.61, 0.77) | 0.83 (0.68, 1.00) | 0.50 (0.48, 0.53) | 0.98 (0.91, 1.05) |
| 90-96 | 0.77 (0.62, 0.96) | 0.74 (0.50, 1.10) | 0.72 (0.63, 0.81) | 0.79 (0.65, 0.96) | 0.54 (0.51, 0.57) | 1.02 (0.95, 1.09) |
| 84-90 | 0.81 (0.66, 1.01) | 0.91 (0.62, 1.32) | 0.70 (0.62, 0.79) | 0.83 (0.69, 1.01) | 0.55 (0.53, 0.58) | 1.01 (0.94, 1.08) |
| 78-84 | 0.81 (0.66, 1.01) | 0.69 (0.46, 1.03) | 0.78 (0.69, 0.88) | 0.83 (0.69, 1.00) | 0.56 (0.53, 0.59) | 1.07 (1.00, 1.15) |
| 72-78 | 0.84 (0.68, 1.04) | 0.81 (0.55, 1.20) | 0.77 (0.68, 0.86) | 0.88 (0.73, 1.06) | 0.56 (0.54, 0.59) | 1.07 (1.00, 1.14) |
| 66-72 | 0.84 (0.68, 1.04) | 0.93 (0.64, 1.34) | 0.88 (0.79, 0.98) | 0.93 (0.77, 1.11) | 0.60 (0.57, 0.63) | 1.16 (1.08, 1.24) |
| 60-66 | 0.75 (0.60, 0.94) | 1.11 (0.78, 1.59) | 0.91 (0.82, 1.02) | 0.97 (0.81, 1.16) | 0.59 (0.56, 0.62) | 1.09 (1.02, 1.16) |
| 54-60 | 0.88 (0.71, 1.08) | 0.94 (0.65, 1.37) | 0.96 (0.86, 1.07) | 0.93 (0.77, 1.11) | 0.63 (0.60, 0.66) | 1.17 (1.10, 1.25) |
| 48-54 | 0.96 (0.79, 1.18) | 1.04 (0.72, 1.49) | 0.93 (0.83, 1.04) | 0.97 (0.81, 1.17) | 0.63 (0.60, 0.66) | 1.08 (1.01, 1.16) |
| 42-48 | 0.90 (0.73, 1.11) | 0.96 (0.67, 1.39) | 0.94 (0.84, 1.05) | 0.86 (0.71, 1.04) | 0.64 (0.61, 0.67) | 1.17 (1.10, 1.25) |
| 36-42 | 0.98 (0.80, 1.20) | 0.81 (0.55, 1.20) | 0.84 (0.75, 0.95) | 0.95 (0.79, 1.15) | 0.64 (0.61, 0.67) | 1.13 (1.06, 1.21) |
| 30-36 | 1.04 (0.85, 1.27) | 0.93 (0.64, 1.34) | 0.91 (0.82, 1.02) | 0.94 (0.79, 1.14) | 0.67 (0.63, 0.70) | 1.19 (1.12, 1.28) |
| 24-30 | 1.07 (0.88, 1.31) | 1.02 (0.71, 1.46) | 0.94 (0.85, 1.05) | 0.92 (0.76, 1.10) | 0.67 (0.64, 0.70) | 1.06 (0.99, 1.14) |
| 18-24 | 0.99 (0.81, 1.21) | 0.94 (0.65, 1.37) | 0.96 (0.86, 1.07) | 1.06 (0.88, 1.26) | 0.69 (0.65, 0.72) | 1.18 (1.10, 1.26) |
| 12-18 | 0.91 (0.74, 1.12) | 1.06 (0.74, 1.51) | 0.98 (0.88, 1.10) | 0.89 (0.74, 1.07) | 0.72 (0.68, 0.75) | 1.03 (0.97, 1.11) |
| 6-12 | 0.87 (0.71, 1.08) | 1.09 (0.76, 1.56) | 0.96 (0.86, 1.07) | 0.98 (0.82, 1.18) | 0.79 (0.75, 0.83) | 1.07 (1.00, 1.14) |
| 0-6 | 1.00 (1.00, 1.00) | 1.00 (1.00, 1.00) | 1.00 (1.00, 1.00) | 1.00 (1.00, 1.00) | 1.00 (1.00, 1.00) | 1.00 (1.00, 1.00) |
| Months prior to index date | Age group: 65-74 | | Age group: 75-84 | |  |  |
|  | Cases;  n=27670 | Controls; n=27670 | Cases;  n=55650 | Controls; n=55650 |  |  |
| 114-120 | 2.23 (2.06, 2.42) | 5.16 (4.57, 5.82) | 2.05 (1.56, 2.70) | 3.96 (2.55, 6.14) |  |  |
| 108-114 | 2.28 (2.10, 2.47) | 4.95 (4.39, 5.60) | 1.65 (1.24, 2.20) | 3.88 (2.50, 6.02) |  |  |
| 102-108 | 2.24 (2.06, 2.43) | 4.82 (4.27, 5.45) | 1.77 (1.34, 2.35) | 3.72 (2.39, 5.78) |  |  |
| 96-102 | 2.06 (1.89, 2.23) | 4.49 (3.97, 5.07) | 1.31 (0.97, 1.76) | 2.80 (1.77, 4.42) |  |  |
| 90-96 | 2.02 (1.86, 2.19) | 4.37 (3.86, 4.94) | 1.25 (0.93, 1.70) | 3.64 (2.34, 5.66) |  |  |
| 84-90 | 2.02 (1.86, 2.19) | 4.13 (3.65, 4.67) | 1.31 (0.97, 1.76) | 2.92 (1.85, 4.60) |  |  |
| 78-84 | 1.92 (1.77, 2.09) | 3.77 (3.32, 4.27) | 1.16 (0.85, 1.58) | 2.80 (1.77, 4.42) |  |  |
| 72-78 | 1.74 (1.60, 1.90) | 3.51 (3.09, 3.98) | 1.11 (0.81, 1.51) | 2.16 (1.34, 3.47) |  |  |
| 66-72 | 1.72 (1.58, 1.88) | 3.47 (3.06, 3.94) | 1.36 (1.01, 1.83) | 2.64 (1.67, 4.18) |  |  |
| 60-66 | 1.61 (1.48, 1.76) | 3.13 (2.75, 3.55) | 1.03 (0.75, 1.41) | 2.16 (1.34, 3.47) |  |  |
| 54-60 | 1.54 (1.42, 1.69) | 2.98 (2.62, 3.39) | 1.09 (0.80, 1.49) | 2.52 (1.59, 4.00) |  |  |
| 48-54 | 1.48 (1.35, 1.61) | 2.72 (2.39, 3.09) | 0.99 (0.72, 1.36) | 1.88 (1.16, 3.05) |  |  |
| 42-48 | 1.30 (1.18, 1.42) | 2.37 (2.07, 2.70) | 1.01 (0.74, 1.39) | 2.56 (1.61, 4.06) |  |  |
| 36-42 | 1.24 (1.13, 1.36) | 2.07 (1.81, 2.37) | 0.75 (0.53, 1.06) | 1.64 (1.00, 2.70) |  |  |
| 30-36 | 1.13 (1.03, 1.24) | 2.10 (1.84, 2.41) | 0.75 (0.53, 1.06) | 1.80 (1.10, 2.93) |  |  |
| 24-30 | 0.97 (0.88, 1.07) | 1.65 (1.43, 1.90) | 0.75 (0.53, 1.06) | 1.28 (0.76, 2.16) |  |  |
| 18-24 | 0.93 (0.85, 1.03) | 1.55 (1.34, 1.79) | 0.88 (0.63, 1.22) | 1.80 (1.10, 2.93) |  |  |
| 12-18 | 0.89 (0.81, 0.99) | 1.35 (1.16, 1.56) | 0.95 (0.68, 1.31) | 1.56 (0.94, 2.58) |  |  |
| 6-12 | 0.93 (0.84, 1.02) | 1.29 (1.11, 1.49) | 0.95 (0.68, 1.31) | 1.04 (0.60, 1.80) |  |  |
| 0-6 | 1.00 (1.00, 1.00) | 1.00 (1.00, 1.00) | 1.00 (1.00, 1.00) | 1.00 (1.00, 1.00) |  |  |

**Supplementary Table 8**

Conditional odds ratios and 95% CIs for primary THR associated with sick leave stratified by age within each 6-month period adjusted for geographical region, ethnicity, neighbourhood deprivation, comorbidity, BMI, smoking status, drinking status, and hip injury.

| Months prior to index date | Age group | | | | |
| --- | --- | --- | --- | --- | --- |
|  | 30-44 | 45-54 | 55-64 | 65-74 | 75-84 |
|  | OR (95% CI) | OR (95% CI) | OR (95% CI) | OR (95% CI) | OR (95% CI) |
| 114-120 | 1.87 (1.28, 2.74) | 1.24 (1.04, 1.47) | 1.12 (1.02, 1.24) | 1.04 (0.96, 1.14) | 1.48 (1.03, 2.10) |
| 108-114 | 1.41 (0.99, 2.02) | 1.13 (0.95, 1.34) | 1.21 (1.09, 1.33) | 1.18 (1.08, 1.29) | 1.27 (0.87, 1.85) |
| 102-108 | 1.33 (0.95, 1.84) | 1.26 (1.07, 1.49) | 1.19 (1.08, 1.31) | 1.17 (1.06, 1.28) | 1.62 (1.12, 2.34) |
| 96-102 | 1.63 (1.16, 2.30) | 1.50 (1.27, 1.77) | 1.21 (1.10, 1.34) | 1.15 (1.05, 1.27) | 1.11 (0.69, 1.81) |
| 90-96 | 1.74 (1.24, 2.44) | 1.41 (1.20, 1.66) | 1.23 (1.12, 1.35) | 1.11 (1.01, 1.22) | 1.03 (0.63, 1.68) |
| 84-90 | 1.29 (0.92, 1.82) | 1.43 (1.22, 1.68) | 1.28 (1.16, 1.40) | 1.27 (1.15, 1.40) | 1.33 (0.83, 2.14) |
| 78-84 | 1.74 (1.21, 2.49) | 1.38 (1.19, 1.61) | 1.24 (1.13, 1.36) | 1.35 (1.22, 1.50) | 0.95 (0.64, 1.43) |
| 72-78 | 1.38 (1.01, 1.87) | 1.32 (1.13, 1.54) | 1.20 (1.10, 1.32) | 1.20 (1.08, 1.33) | 2.02 (1.13, 3.61) |
| 66-72 | 1.53 (1.09, 2.15) | 1.33 (1.15, 1.54) | 1.14 (1.04, 1.24) | 1.24 (1.12, 1.38) | 1.33 (0.82, 2.18) |
| 60-66 | 1.48 (1.04, 2.10) | 1.18 (1.02, 1.37) | 1.17 (1.07, 1.28) | 1.22 (1.09, 1.37) | 1.34 (0.78, 2.30) |
| 54-60 | 1.10 (0.81, 1.50) | 1.31 (1.14, 1.52) | 1.20 (1.10, 1.31) | 1.26 (1.13, 1.42) | 0.94 (0.57, 1.54) |
| 48-54 | 1.27 (0.94, 1.71) | 1.34 (1.17, 1.55) | 1.34 (1.23, 1.47) | 1.30 (1.16, 1.47) | 1.44 (0.82, 2.51) |
| 42-48 | 1.39 (1.39, 1.39) | 1.41 (1.22, 1.62) | 1.27 (1.17, 1.39) | 1.36 (1.20, 1.54) | 0.99 (0.61, 1.63) |
| 36-42 | 1.50 (1.12, 2.02) | 1.49 (1.29, 1.72) | 1.38 (1.27, 1.51) | 1.56 (1.37, 1.78) | 1.36 (0.58, 3.18) |
| 30-36 | 1.22 (0.90, 1.64) | 1.51 (1.31, 1.73) | 1.23 (1.13, 1.34) | 1.36 (1.19, 1.56) | 0.77 (0.41, 1.45) |
| 24-30 | 1.36 (1.02, 1.80) | 1.58 (1.38, 1.81) | 1.50 (1.37, 1.63) | 1.54 (1.33, 1.78) | - |
| 18-24 | 1.51 (1.12, 2.03) | 1.64 (1.43, 1.88) | 1.32 (1.21, 1.43) | 1.52 (1.30, 1.78) | 1.36 (0.75, 2.45) |
| 12-18 | 1.71 (1.27, 2.29) | 1.76 (1.53, 2.02) | 1.68 (1.53, 1.83) | 1.62 (1.38, 1.91) | 2.16 (1.16, 4.02) |
| 6-12 | 2.29 (1.70, 3.09) | 1.99 (1.75, 2.28) | 1.74 (1.60, 1.90) | 1.91 (1.62, 2.25) | - |
| 0-6 | 2.14 (1.66, 2.75) | 2.49 (2.19, 2.84) | 2.45 (2.26, 2.67) | 2.62 (2.20, 3.12) | 5.53 (5.53, 5.53) |
