# Supplementary material for: Clinical care patterns in the 10-years prior to primary total hip replacement: a matched population-based case-control analysis in England
Source: BMC Med. 2026 Jun 24;24:480. doi: 10.1186/s12916-026-05011-7 (PMC13560152; doi:10.1186/s12916-026-05011-7)
Supplement: Supplementary file 4 [file 12916_2026_5011_MOESM5_ESM.docx]

**Supplementary Figures 1 to 6 – Prevalence Stratified by Age**

Supplementary Figure 1. Prevalence and 95% CIs of each care strategy within 10 years prior to index date in case and control groups, overall and by gender among individuals aged 30-44 years.

Grey, blue and red line indicates prevalence for overall, men and women, respectively. Triangle line and dot line represents prevalence of control and case group, respectively.

Supplementary Figure 2. Prevalence and 95% CIs of each care strategy within 10 years prior to index date in case and control groups, overall and by gender among individuals aged 45-54 years.

Grey, blue and red line indicates prevalence for overall, men and women, respectively. Triangle line and dot line represents prevalence of control and case group, respectively.

Supplementary Figure 3. Prevalence and 95% CIs of each care strategy within 10 years prior to index date in case and control groups, overall and by gender among individuals aged 55-64 years.

Grey, blue and red line indicates prevalence for overall, men and women, respectively. Triangle line and dot line represents prevalence of control and case group, respectively.

Supplementary Figure 4. Prevalence and 95% CIs of each care strategy within 10 years prior to index date in case and control groups, overall and by gender among individuals aged 65-74 years.

Grey, blue and red line indicates prevalence for overall, men and women, respectively. Triangle line and dot line represents prevalence of control and case group, respectively.

Supplementary Figure 5. Prevalence and 95% CIs of each care strategy within 10 years prior to index date in case and control groups, overall and by gender among individuals aged 75-84 years.

Grey, blue and red line indicates prevalence for overall, men and women, respectively. Triangle line and dot line represents prevalence of control and case group, respectively.

Supplementary Figure 6. Prevalence and 95% CIs of each care strategy within 10 years prior to index date in case and control groups, overall and by gender among individuals aged 85 years and over.

Grey, blue and red line indicates prevalence for overall, men and women, respectively. Triangle line and dot line represents prevalence of control and case group, respectively. No value in the window indicates model did not converge within that window.

**Supplementary Figures 7 to 12 – Prevalence Stratified by Ethnicity**

Supplementary Figure 7. Prevalence and 95% CIs of each care strategy within 10 years prior to index date in case and control groups, overall and by gender among White ethnic group.

Grey, blue and red line indicates prevalence for overall, men and women, respectively. Triangle line and dot line represents prevalence of control and case group, respectively.

Supplementary Figure 8. Prevalence and 95% CIs of each care strategy within 10 years prior to index date in case and control groups, overall and by gender among Mixed/Multiple ethnic group

Grey, blue and red line indicates prevalence for overall, men and women, respectively. Triangle line and dot line represents prevalence of control and case group, respectively. No value in the window indicates model did not converge within that window.

Supplementary Figure 9. Prevalence and 95% CIs of each care strategy within 10 years prior to index date in case and control groups, overall and by gender among South Asian ethnic group

Grey, blue and red line indicates prevalence for overall, men and women, respectively. Triangle line and dot line represents prevalence of control and case group, respectively. No value in the window indicates model did not converge within that window.

Supplementary Figure 10. Prevalence and 95% CIs of each care strategy within 10 years prior to index date in case and control groups, overall and by gender among Other Asian ethnic group

Grey, blue and red line indicates prevalence for overall, men and women, respectively. Triangle line and dot line represents prevalence of control and case group, respectively. No value in the window indicates model did not converge within that window.

Supplementary Figure 11. Prevalence and 95% CIs of each care strategy within 10 years prior to index date in case and control groups, overall and by gender among Black ethnic group

Grey, blue and red line indicates prevalence for overall, men and women, respectively. Triangle line and dot line represents prevalence of control and case group, respectively. No value in the window indicates model did not converge within that window.

Supplementary Figure 12. Prevalence and 95% CIs of each care strategy within 10 years prior to index date in case and control groups, overall and by gender among Other/Unknown ethnic group

Grey, blue and red line indicates prevalence for overall, men and women, respectively. Triangle line and dot line represents prevalence of control and case group, respectively. No value in the window indicates model did not converge within that window.

**Supplementary** **Figures 13 to 21 – Prevalence Stratified by Region**

Supplementary Figure 13. Prevalence and 95% CIs of each care strategy within 10 years prior to index date in case and control groups, overall and by gender in the North East region

Grey, blue and red line indicates prevalence for overall, men and women, respectively. Triangle line and dot line represents prevalence of control and case group, respectively.

Supplementary Figure 14. Prevalence and 95% CIs of each care strategy within 10 years prior to index date in case and control groups, overall and by gender in the North West region

Grey, blue and red line indicates prevalence for overall, men and women, respectively. Triangle line and dot line represents prevalence of control and case group, respectively.

Supplementary Figure 15. Prevalence and 95% CIs of each care strategy within 10 years prior to index date in case and control groups, overall and by gender in the Yorkshire and The Humber region

Grey, blue and red line indicates prevalence for overall, men and women, respectively. Triangle line and dot line represents prevalence of control and case group, respectively.

Supplementary Figure 16. Prevalence and 95% CIs of each care strategy within 10 years prior to index date in case and control groups, overall and by gender in the East Midlands region

Grey, blue and red line indicates prevalence for overall, men and women, respectively. Triangle line and dot line represents prevalence of control and case group, respectively.

Supplementary Figure 17. Prevalence and 95% CIs of each care strategy within 10 years prior to index date in case and control groups, overall and by gender in the West Midlands region

Grey, blue and red line indicates prevalence for overall, men and women, respectively. Triangle line and dot line represents prevalence of control and case group, respectively.

Supplementary Figure 18. Prevalence and 95% CIs of each care strategy within 10 years prior to index date in case and control groups, overall and by gender in the East of England region

Grey, blue and red line indicates prevalence for overall, men and women, respectively. Triangle line and dot line represents prevalence of control and case group, respectively.

Supplementary Figure 19. Prevalence and 95% CIs of each care strategy within 10 years prior to index date in case and control groups, overall and by gender in the London region

Grey, blue and red line indicates prevalence for overall, men and women, respectively. Triangle line and dot line represents prevalence of control and case group, respectively.

Supplementary Figure 20. Prevalence and 95% CIs of each care strategy within 10 years prior to index date in case and control groups, overall and by gender in the South East region

Grey, blue and red line indicates prevalence for overall, men and women, respectively. Triangle line and dot line represents prevalence of control and case group, respectively.

Supplementary Figure 21. Prevalence and 95% CIs of each care strategy within 10 years prior to index date in case and control groups, overall and by gender in the South West region

Grey, blue and red line indicates prevalence for overall, men and women, respectively. Triangle line and dot line represents prevalence of control and case group, respectively.

**Supplementary Figures 22 to 31 – Prevalence Stratified by IMD**

Supplementary Figure 22. Prevalence and 95% CIs of each care strategy within 10 years prior to index date in case and control groups, overall and by gender in IMD1

Grey, blue and red line indicates prevalence for overall, men and women, respectively. Triangle line and dot line represents prevalence of control and case group, respectively.

Supplementary Figure 23. Prevalence and 95% CIs of each care strategy within 10 years prior to index date in case and control groups, overall and by gender in IMD2

Grey, blue and red line indicates prevalence for overall, men and women, respectively. Triangle line and dot line represents prevalence of control and case group, respectively.

Supplementary Figure 24. Prevalence and 95% CIs of each care strategy within 10 years prior to index date in case and control groups, overall and by gender in IMD3

Grey, blue and red line indicates prevalence for overall, men and women, respectively. Triangle line and dot line represents prevalence of control and case group, respectively.

Supplementary Figure 25. Prevalence and 95% CIs of each care strategy within 10 years prior to index date in case and control groups, overall and by gender in IMD4

Grey, blue and red line indicates prevalence for overall, men and women, respectively. Triangle line and dot line represents prevalence of control and case group, respectively.

Supplementary Figure 26. Prevalence and 95% CIs of each care strategy within 10 years prior to index date in case and control groups, overall and by gender in IMD5

Grey, blue and red line indicates prevalence for overall, men and women, respectively. Triangle line and dot line represents prevalence of control and case group, respectively.

Supplementary Figure 27. Prevalence and 95% CIs of each care strategy within 10 years prior to index date in case and control groups, overall and by gender in IMD6

Grey, blue and red line indicates prevalence for overall, men and women, respectively. Triangle line and dot line represents prevalence of control and case group, respectively.

Supplementary Figure 28. Prevalence and 95% CIs of each care strategy within 10 years prior to index date in case and control groups, overall and by gender in IMD7

Grey, blue and red line indicates prevalence for overall, men and women, respectively. Triangle line and dot line represents prevalence of control and case group, respectively.

Supplementary Figure 29. Prevalence and 95% CIs of each care strategy within 10 years prior to index date in case and control groups, overall and by gender in IMD8

Grey, blue and red line indicates prevalence for overall, men and women, respectively. Triangle line and dot line represents prevalence of control and case group, respectively.

Supplementary Figure 30. Prevalence and 95% CIs of each care strategy within 10 years prior to index date in case and control groups, overall and by gender in IMD9

Grey, blue and red line indicates prevalence for overall, men and women, respectively. Triangle line and dot line represents prevalence of control and case group, respectively.

Supplementary Figure 31. Prevalence and 95% CIs of each care strategy within 10 years prior to index date in case and control groups, overall and by gender in IMD10

Grey, blue and red line indicates prevalence for overall, men and women, respectively. Triangle line and dot line represents prevalence of control and case group, respectively.

**Supplementary Figures 32 to 37 – PRR Stratified by Age**

Supplementary Figure 32. Prevalence rate ratio and 95% CIs of each care strategy in each 6-month interval with most recent 6-month interval among individuals aged 30-44 years adjusted for sex, geographical region, ethnicity, neighbourhood deprivation, comorbidity, BMI, smoking status, drinking status, and hip injury

Triangle line and dot line represents prevalence of control and case group, respectively.

Supplementary Figure 33. Prevalence rate ratio and 95% CIs of each care strategy in each 6-month interval with most recent 6-month interval among individuals aged 45-54 years adjusted for sex, geographical region, ethnicity, neighbourhood deprivation, comorbidity, BMI, smoking status, drinking status, and hip injury

Triangle line and dot line represents prevalence of control and case group, respectively.

Supplementary Figure 34. Prevalence rate ratio and 95% CIs of each care strategy in each 6-month interval with most recent 6-month interval among individuals aged 55-64 years adjusted for sex, geographical region, ethnicity, neighbourhood deprivation, comorbidity, BMI, smoking status, drinking status, and hip injury

Triangle line and dot line represents prevalence of control and case group, respectively.

Supplementary Figure 35. Prevalence rate ratio and 95% CIs of each care strategy in each 6-month interval with most recent 6-month interval among individuals aged 65-74 years adjusted for sex, geographical region, ethnicity, neighbourhood deprivation, comorbidity, BMI, smoking status, drinking status, and hip injury

Triangle line and dot line represents prevalence of control and case group, respectively.

Supplementary Figure 36. Prevalence rate ratio and 95% CIs of each care strategy in each 6-month interval with most recent 6-month interval among individuals aged 75-84 years adjusted for sex, geographical region, ethnicity, neighbourhood deprivation, comorbidity, BMI, smoking status, drinking status, and hip injury

Triangle line and dot line represents prevalence of control and case group, respectively.

Supplementary Figure 37. Prevalence rate ratio and 95% CIs of each care strategy in each 6-month interval with most recent 6-month interval among individuals aged 85+ years adjusted for sex, geographical region, ethnicity, neighbourhood deprivation, comorbidity, BMI, smoking status, drinking status, and hip injury

Triangle line and dot line represents prevalence of control and case group, respectively.

**Supplementary Figures 38 to 43 – PRR Stratified by Ethnicity**

Supplementary Figure 38. Prevalence rate ratio and 95% CIs of each care strategy in each 6-month interval with most recent 6-month interval among White ethnic group adjusted for sex, age, geographical region, neighbourhood deprivation, comorbidity, BMI, smoking status, drinking status, and hip injury

Triangle line and dot line represents prevalence of control and case group, respectively.

Supplementary Figure 39. Prevalence rate ratio and 95% CIs of each care strategy in each 6-month interval with most recent 6-month interval among Mixed/Multiple ethnic group adjusted for sex, age, geographical region, neighbourhood deprivation, comorbidity, BMI, smoking status, drinking status, and hip injury

Triangle line and dot line represents prevalence of control and case group, respectively. Models for GP visits, referrals for imaging and referrals to pain clinic did not converge.

Supplementary Figure 40. Prevalence rate ratio and 95% CIs of each care strategy in each 6-month interval with most recent 6-month interval among South Asian ethnic group adjusted for sex, age, geographical region, neighbourhood deprivation, comorbidity, BMI, smoking status, drinking status, and hip injury

Triangle line and dot line represents prevalence of control and case group, respectively. Models for GP visits, referrals for imaging and referrals to pain clinic did not converge.

Supplementary Figure 41. Prevalence rate ratio and 95% CIs of each care strategy in each 6-month interval with most recent 6-month interval among Other Asian ethnic group adjusted for sex, age, geographical region, neighbourhood deprivation, comorbidity, BMI, smoking status, drinking status, and hip injury

Triangle line and dot line represents prevalence of control and case group, respectively. Models for GP visits, referrals for imaging and referrals to pain clinic did not converge.

Supplementary Figure 42. Prevalence rate ratio and 95% CIs of each care strategy in each 6-month interval with most recent 6-month interval among Other Asian ethnic group adjusted for sex, age, geographical region, neighbourhood deprivation, comorbidity, BMI, smoking status, drinking status, and hip injury

Triangle line and dot line represents prevalence of control and case group, respectively. Models for GP visits, referrals for imaging and referrals to pain clinic did not converge.

Supplementary Figure 43. Prevalence rate ratio and 95% CIs of each care strategy in each 6-month interval with most recent 6-month interval among Other/Unknown ethnic group adjusted for sex, age, geographical region, neighbourhood deprivation, comorbidity, BMI, smoking status, drinking status, and hip injury

Triangle line and dot line represents prevalence of control and case group, respectively. Models for GP visits, referrals for imaging and referrals to pain clinic did not converge.

**Supplementary Figures 44 to 52 – PRR Stratified by Region**

Supplementary Figure 44. Prevalence rate ratio and 95% CIs of each care strategy in each 6-month interval with most recent 6-month interval in the North East adjusted for sex, age, ethnicity, neighbourhood deprivation, comorbidity, BMI, smoking status, drinking status, and hip injury

Triangle line and dot line represents prevalence of control and case group, respectively.

Supplementary Figure 45. Prevalence rate ratio and 95% CIs of each care strategy in each 6-month interval with most recent 6-month interval in the North West region adjusted for sex, age, ethnicity, neighbourhood deprivation, comorbidity, BMI, smoking status, drinking status, and hip injury

Triangle line and dot line represents prevalence of control and case group, respectively.

Supplementary Figure 46. Prevalence rate ratio and 95% CIs of each care strategy in each 6-month interval with most recent 6-month interval in the Yorkshire and The Humber region adjusted for sex, age, ethnicity, neighbourhood deprivation, comorbidity, BMI, smoking status, drinking status, and hip injury

Triangle line and dot line represents prevalence of control and case group, respectively.

Supplementary Figure 47. Prevalence rate ratio and 95% CIs of each care strategy in each 6-month interval with most recent 6-month interval in the East Midlands region adjusted for sex, age, ethnicity, neighbourhood deprivation, comorbidity, BMI, smoking status, drinking status, and hip injury

Triangle line and dot line represents prevalence of control and case group, respectively.

Supplementary Figure 48. Prevalence rate ratio and 95% CIs of each care strategy in each 6-month interval with most recent 6-month interval in the West Midlands region adjusted for sex, age, ethnicity, neighbourhood deprivation, comorbidity, BMI, smoking status, drinking status, and hip injury

Triangle line and dot line represents prevalence of control and case group, respectively.

Supplementary Figure 49. Prevalence rate ratio and 95% CIs of each care strategy in each 6-month interval with most recent 6-month interval in the East of England region adjusted for sex, age, ethnicity, neighbourhood deprivation, comorbidity, BMI, smoking status, drinking status, and hip injury

Triangle line and dot line represents prevalence of control and case group, respectively.

Supplementary Figure 50. Prevalence rate ratio and 95% CIs of each care strategy in each 6-month interval with most recent 6-month interval in the London region adjusted for sex, age, ethnicity, neighbourhood deprivation, comorbidity, BMI, smoking status, drinking status, and hip injury

Triangle line and dot line represents prevalence of control and case group, respectively.

Supplementary Figure 51. Prevalence rate ratio and 95% CIs of each care strategy in each 6-month interval with most recent 6-month interval in the South East region adjusted for sex, age, ethnicity, neighbourhood deprivation, comorbidity, BMI, smoking status, drinking status, and hip injury

Triangle line and dot line represents prevalence of control and case group, respectively.

Supplementary Figure 52. Prevalence rate ratio and 95% CIs of each care strategy in each 6-month interval with most recent 6-month interval in the South West region adjusted for sex, age, ethnicity, neighbourhood deprivation, comorbidity, BMI, smoking status, drinking status, and hip injury

Triangle line and dot line represents prevalence of control and case group, respectively.

**Supplementary Figures 53 to 62 – PRR Stratified by IMD**

Supplementary Figure 53. Prevalence rate ratio and 95% CIs of each care strategy in each 6-month interval with most recent 6-month interval in IMD1 adjusted for sex, age, geographical region, ethnicity, comorbidity, BMI, smoking status, drinking status, and hip injury

Triangle line and dot line represents prevalence of control and case group, respectively.

Supplementary Figure 54. Prevalence rate ratio and 95% CIs of each care strategy in each 6-month interval with most recent 6-month interval in IMD2 adjusted for sex, age, geographical region, ethnicity, comorbidity, BMI, smoking status, drinking status, and hip injury

Triangle line and dot line represents prevalence of control and case group, respectively.

Supplementary Figure 55. Prevalence rate ratio and 95% CIs of each care strategy in each 6-month interval with most recent 6-month interval in IMD3 adjusted for sex, age, geographical region, ethnicity, comorbidity, BMI, smoking status, drinking status, and hip injury

Triangle line and dot line represents prevalence of control and case group, respectively.

Supplementary Figure 56. Prevalence rate ratio and 95% CIs of each care strategy in each 6-month interval with most recent 6-month interval in IMD4 adjusted for sex, age, geographical region, ethnicity, comorbidity, BMI, smoking status, drinking status, and hip injury

Triangle line and dot line represents prevalence of control and case group, respectively.

Supplementary Figure 57. Prevalence rate ratio and 95% CIs of each care strategy in each 6-month interval with most recent 6-month interval in IMD5 adjusted for sex, age, geographical region, ethnicity, comorbidity, BMI, smoking status, drinking status, and hip injury

Triangle line and dot line represents prevalence of control and case group, respectively.

Supplementary Figure 58. Prevalence rate ratio and 95% CIs of each care strategy in each 6-month interval with most recent 6-month interval in IMD6 adjusted for sex, age, geographical region, ethnicity, comorbidity, BMI, smoking status, drinking status, and hip injury

Triangle line and dot line represents prevalence of control and case group, respectively.

Supplementary Figure 59. Prevalence rate ratio and 95% CIs of each care strategy in each 6-month interval with most recent 6-month interval in IMD7 adjusted for sex, age, geographical region, ethnicity, comorbidity, BMI, smoking status, drinking status, and hip injury

Triangle line and dot line represents prevalence of control and case group, respectively.

Supplementary Figure 60. Prevalence rate ratio and 95% CIs of each care strategy in each 6-month interval with most recent 6-month interval in IMD8 adjusted for sex, age, geographical region, ethnicity, comorbidity, BMI, smoking status, drinking status, and hip injury

Triangle line and dot line represents prevalence of control and case group, respectively.

Supplementary Figure 61. Prevalence rate ratio and 95% CIs of each care strategy in each 6-month interval with most recent 6-month interval in IMD9 adjusted for sex, age, geographical region, ethnicity, comorbidity, BMI, smoking status, drinking status, and hip injury

Triangle line and dot line represents prevalence of control and case group, respectively.

Supplementary Figure 62. Prevalence rate ratio and 95% CIs of each care strategy in each 6-month interval with most recent 6-month interval in IMD10 adjusted for sex, age, geographical region, ethnicity, comorbidity, BMI, smoking status, drinking status, and hip injury

Triangle line and dot line represents prevalence of control and case group, respectively.

**Supplementary Figures 63 to 68 – OR Stratified by Age**

Supplementary Figure 63. Conditional odds ratios and 95% CIs for primary THR associated with each care strategy within each 6-month period among individuals aged 30-44 years adjusted for geographical region, ethnicity, neighbourhood deprivation, comorbidity, BMI, smoking status, drinking status, and hip injury

No value in the window indicates model did not converge within that window.

Supplementary Figure 64. Conditional odds ratios and 95% CIs for primary THR associated with each care strategy within each 6-month period among individuals aged 45-54 years adjusted for geographical region, ethnicity, neighbourhood deprivation, comorbidity, BMI, smoking status, drinking status, and hip injury

No value in the window indicates model did not converge within that window.

Supplementary Figure 65. Conditional odds ratios and 95% CIs for primary THR associated with each care strategy within each 6-month period among individuals aged 55-64 years adjusted for geographical region, ethnicity, neighbourhood deprivation, comorbidity, BMI, smoking status, drinking status, and hip injury

No value in the window indicates model did not converge within that window.

Supplementary Figure 66. Conditional odds ratios and 95% CIs for primary THR associated with each care strategy within each 6-month period among individuals aged 65-74 years adjusted for geographical region, ethnicity, neighbourhood deprivation, comorbidity, BMI, smoking status, drinking status, and hip injury

No value in the window indicates model did not converge within that window.

Supplementary Figure 67. Conditional odds ratios and 95% CIs for primary THR associated with each care strategy within each 6-month period among individuals aged 75-84 years adjusted for geographical region, ethnicity, neighbourhood deprivation, comorbidity, BMI, smoking status, drinking status, and hip injury

No value in the window indicates model did not converge within that window.

Supplementary Figure 68. Conditional odds ratios and 95% CIs for primary THR associated with each care strategy within each 6-month period among individuals aged 85+ years adjusted for geographical region, ethnicity, neighbourhood deprivation, comorbidity, BMI, smoking status, drinking status, and hip injury

No value in the window indicates model did not converge within that window.

**Supplementary Figures 69 to 74 – OR Stratified by Ethnicity**

Supplementary Figure 69. Conditional odds ratios and 95% CIs for primary THR associated with each care strategy within each 6-month period among White ethnic group adjusted for geographical region, neighbourhood deprivation, comorbidity, BMI, smoking status, drinking status, and hip injury

No value in the window indicates model did not converge within that window.

Supplementary Figure 70. Conditional odds ratios and 95% CIs for primary THR associated with each care strategy within each 6-month period among Mixed/Multiple ethnic group (unadjusted)

No value in the window indicates model did not converge within that window.

Supplementary Figure 71. Conditional odds ratios and 95% CIs for primary THR associated with each care strategy within each 6-month period among South Asian ethnic group (unadjusted)

No value in the window indicates model did not converge within that window.

Supplementary Figure 72. Conditional odds ratios and 95% CIs for primary THR associated with each care strategy within each 6-month period among Other Asian ethnic group (unadjusted)

No value in the window indicates model did not converge within that window.

Supplementary Figure 73. Conditional odds ratios and 95% CIs for primary THR associated with each care strategy within each 6-month period among Black ethnic group (unadjusted)

No value in the window indicates model did not converge within that window.

Supplementary Figure 74. Conditional odds ratios and 95% CIs for primary THR associated with each care strategy within each 6-month period among Other/Unknown ethnic group (unadjusted)

No value in the window indicates model did not converge within that window.

**Supplementary Figures 75 to 83 – OR Stratified by Region**

Supplementary Figure 75. Conditional odds ratios and 95% CIs for primary THR associated with each care strategy within each 6-month period in the North East region adjusted for ethnicity, neighbourhood deprivation, comorbidity, BMI, smoking status, drinking status, and hip injury

No value in the window indicates model did not converge within that window.

Supplementary Figure 76. Conditional odds ratios and 95% CIs for primary THR associated with each care strategy within each 6-month period in the North West region adjusted for ethnicity, neighbourhood deprivation, comorbidity, BMI, smoking status, drinking status, and hip injury

No value in the window indicates model did not converge within that window.

Supplementary Figure 77. Conditional odds ratios and 95% CIs for primary THR associated with each care strategy within each 6-month period in the Yorkshire and The Humber region adjusted for ethnicity, neighbourhood deprivation, comorbidity, BMI, smoking status, drinking status, and hip injury

No value in the window indicates model did not converge within that window.

Supplementary Figure 78. Conditional odds ratios and 95% CIs for primary THR associated with each care strategy within each 6-month period in the East Midlands region adjusted for ethnicity, neighbourhood deprivation, comorbidity, BMI, smoking status, drinking status, and hip injury

No value in the window indicates model did not converge within that window.

Supplementary Figure 79. Conditional odds ratios and 95% CIs for primary THR associated with each care strategy within each 6-month period in the West Midlands region adjusted for ethnicity, neighbourhood deprivation, comorbidity, BMI, smoking status, drinking status, and hip injury

No value in the window indicates model did not converge within that window.

Supplementary Figure 80. Conditional odds ratios and 95% CIs for primary THR associated with each care strategy within each 6-month period in the East of England region adjusted for ethnicity, neighbourhood deprivation, comorbidity, BMI, smoking status, drinking status, and hip injury

No value in the window indicates model did not converge within that window.

Supplementary Figure 81. Conditional odds ratios and 95% CIs for primary THR associated with each care strategy within each 6-month period in the London region adjusted for ethnicity, neighbourhood deprivation, comorbidity, BMI, smoking status, drinking status, and hip injury

No value in the window indicates model did not converge within that window.

Supplementary Figure 82. Conditional odds ratios and 95% CIs for primary THR associated with each care strategy within each 6-month period in the South East adjusted for ethnicity, neighbourhood deprivation, comorbidity, BMI, smoking status, drinking status, and hip injury

No value in the window indicates model did not converge within that window.

Supplementary Figure 83. Conditional odds ratios and 95% CIs for primary THR associated with each care strategy within each 6-month period in the South West adjusted for ethnicity, neighbourhood deprivation, comorbidity, BMI, smoking status, drinking status, and hip injury

No value in the window indicates model did not converge within that window.

**Supplementary Figures 84 to 93 – OR Stratified by IMD**

Supplementary Figure 84. Conditional odds ratios and 95% CIs for primary THR associated with each care strategy within each 6-month period in IMD1 adjusted for geographical region, ethnicity, comorbidity, BMI, smoking status, drinking status, and hip injury

No value in the window indicates model did not converge within that window.

Supplementary Figure 85. Conditional odds ratios and 95% CIs for primary THR associated with each care strategy within each 6-month period in IMD2 adjusted for geographical region, ethnicity, comorbidity, BMI, smoking status, drinking status, and hip injury

No value in the window indicates model did not converge within that window.

Supplementary Figure 86. Conditional odds ratios and 95% CIs for primary THR associated with each care strategy within each 6-month period in IMD3 adjusted for geographical region, ethnicity, comorbidity, BMI, smoking status, drinking status, and hip injury

No value in the window indicates model did not converge within that window.

Supplementary Figure 87. Conditional odds ratios and 95% CIs for primary THR associated with each care strategy within each 6-month period in IMD4 adjusted for geographical region, ethnicity, comorbidity, BMI, smoking status, drinking status, and hip injury

No value in the window indicates model did not converge within that window.

Supplementary Figure 88. Conditional odds ratios and 95% CIs for primary THR associated with each care strategy within each 6-month period in IMD5 adjusted for geographical region, ethnicity, comorbidity, BMI, smoking status, drinking status, and hip injury

No value in the window indicates model did not converge within that window.

Supplementary Figure 89. Conditional odds ratios and 95% CIs for primary THR associated with each care strategy within each 6-month period in IMD6 adjusted for geographical region, ethnicity, comorbidity, BMI, smoking status, drinking status, and hip injury

No value in the window indicates model did not converge within that window.

Supplementary Figure 90. Conditional odds ratios and 95% CIs for primary THR associated with each care strategy within each 6-month period in IMD7 adjusted for geographical region, ethnicity, comorbidity, BMI, smoking status, drinking status, and hip injury

No value in the window indicates model did not converge within that window.

Supplementary Figure 91. Conditional odds ratios and 95% CIs for primary THR associated with each care strategy within each 6-month period in IMD8 adjusted for geographical region, ethnicity, comorbidity, BMI, smoking status, drinking status, and hip injury

No value in the window indicates model did not converge within that window.

Supplementary Figure 92. Conditional odds ratios and 95% CIs for primary THR associated with each care strategy within each 6-month period in IMD9 adjusted for geographical region, ethnicity, comorbidity, BMI, smoking status, drinking status, and hip injury

No value in the window indicates model did not converge within that window.

Supplementary Figure 93. Conditional odds ratios and 95% CIs for primary THR associated with each care strategy within each 6-month period in IMD10 adjusted for geographical region, ethnicity, comorbidity, BMI, smoking status, drinking status, and hip injury

No value in the window indicates model did not converge within that window.

**Supplementary Figures 94 to 98 – Sensitivity Analysis**

Supplementary Figure 94. Prevalence and 95% CIs of each care strategy within 10 years prior to index date in case and control groups, overall and by gender. Restricted to the pre-pandemic period 2007 to 2019.

Grey, blue and red line indicates prevalence for overall, men and women, respectively. Triangle line and dot line represents prevalence of control and case group, respectively.

Supplementary Figure 95. Prevalence rate ratios and 95% CIs of each care strategy in each 6-month interval against the most recent 6-month period adjusted for sex, age, geographical region, ethnicity, neighbourhood deprivation, comorbidity status, BMI, smoking status, drinking status, and hip injury. Restricted to the pre-pandemic period 2007 to 2019.

Grey, blue and red lines indicate the PRR for overall, men and women, respectively. Triangle and dot lines represent the PRR of the control and case group, respectively.

Supplementary Figure 96. Conditional odds ratios and 95% CIs for primary THR associated with each care strategy within each 6-month period adjusted for geographical region, ethnicity, comorbidity status, BMI, smoking status, drinking status, and hip injury. Restricted to the pre-pandemic period 2007 to 2019.

Grey, blue and red line indicate ORs for overall, men and women, respectively.

Supplementary Figure 97. Prevalence rate ratios and 95% CIs of antidepressants and antiepileptics in each 6-month interval against the most recent 6-month period adjusted for sex, age, geographical region, ethnicity, neighbourhood deprivation, comorbidity status, BMI, smoking status, drinking status, hip injury, depression/anxiety/stress and epilepsy.

Grey, blue and red lines indicate the PRR for overall, men and women, respectively. Triangle and dot lines represent the PRR of the control and case group, respectively.

Supplementary Figure 98. Conditional odds ratios and 95% CIs for primary THR associated with antidepressants and antiepileptics within each 6-month period adjusted for sex, age, geographical region, ethnicity, neighbourhood deprivation, comorbidity status, BMI, smoking status, drinking status, hip injury, depression/anxiety/stress and epilepsy.

Grey, blue and red line indicate ORs for overall, men and women, respectively.
